# Effectiveness of interventions to prevent falls for people with multiple sclerosis, Parkinson’s disease and stroke: an umbrella review

**DOI:** 10.1186/s12883-021-02402-6

**Published:** 2021-09-29

**Authors:** Nicola O’Malley, Amanda M. Clifford, Mairéad Conneely, Bláthín Casey, Susan Coote

**Affiliations:** 1 School of Allied Health, Faculty of Education and Health Sciences, University of Limerick, Limerick, Ireland; 2 Ageing Research Centre, Health Research Institute, University of Limerick, Limerick, Ireland; 3 Department of Physical Education and Sport Sciences, Faculty of Education and Health Sciences, University of Limerick, Limerick, Ireland; 4 Centre of Physical Activity for Health, Health Research Institute, University of Limerick, Limerick, Ireland; 5 Multiple Sclerosis Society of Ireland, Limerick, Ireland

**Keywords:** Parkinson’s disease, Stroke, Multiple sclerosis, Falls, Umbrella review

## Abstract

**Background:**

The implementation of condition-specific falls prevention interventions is proving challenging due to lack of critical mass and resources. Given the similarities in falls risk factors across stroke, Parkinson’s Disease (PD) and Multiple Sclerosis (MS), the development of an intervention designed for groups comprising of people with these three neurological conditions may provide a pragmatic solution to these challenges. The aims of this umbrella review were to investigate the effectiveness of falls prevention interventions in MS, PD and stroke, and to identify the commonalities and differences between effective interventions for each condition to inform the development of an intervention for mixed neurological groups.

**Methods:**

A systematic literature search was conducted using 15 electronic databases, grey literature searches and hand-screening of reference lists. Systematic reviews of studies investigating the effects of falls prevention interventions in MS, PD and stroke were included. Methodological quality of reviews was assessed using the A MeaSurement Tool to Assess Systematic Reviews 2. A matrix of evidence table was used to assess the degree of overlap. The Grading of Recommendations Assessments, Development and Evaluation framework was used to rate the quality of evidence. Findings were presented through narrative synthesis and a summary of evidence table.

**Results:**

Eighteen reviews were included; three investigating effectiveness of falls prevention interventions in MS, 11 in PD, three in stroke, and one in both PD and stroke. Exercise-based interventions were the most commonly investigated for all three conditions, but differences were identified in the content and delivery of these interventions. Low to moderate quality evidence was found for the effectiveness of exercise-based interventions at reducing falls in PD. Best available evidence suggests that exercise is effective at reducing falls in stroke but no evidence of effect was identified in MS.

**Conclusions:**

The findings suggest that exercise-based interventions are effective at reducing falls in PD, however, the evidence for MS and stroke is less conclusive. A strong theoretical rationale remains for the use of exercise-based interventions to address modifiable physiological falls risk factors for people with MS, PD and stroke, supporting the feasibility of a mixed-diagnosis intervention. Given the high overlap and low methodological quality of primary studies, the focus should be on the development of high-quality trials investigating the effectiveness of falls prevention interventions, rather than the publication of further systematic reviews.

**Supplementary Information:**

The online version contains supplementary material available at 10.1186/s12883-021-02402-6.

## Background

Up to 73% of stroke survivors fall in their first year post-stroke and over half of people with Multiple Sclerosis (MS) (56%) and Parkinson’s Disease (PD) (59%) experience a fall over a three- or six-month period, respectively [1–3]. Falls can result in physical injury for people with these neurological conditions, with research suggesting that between 11 and 17% of falls result in injury [4–6]. Notably, this figure was reported to be as high as 72% among stroke survivors [7]. Falls can have a number of psychosocial consequences including an increase in fear of falling and reduced self-efficacy [8], resulting in decreased independence and health-related quality of life [9, 10]. Falls also lead to an increase in acute healthcare utilisation, higher home-care needs and/or greater institutional care needs [5–7, 11]. Consequently, the development of effective evidence-based falls prevention interventions for people with MS, PD and stroke is a priority for research and service provision.

There are currently substantial shortcomings in the provision of services for people with neurological conditions in Ireland [12]. Despite the recent proliferation in the number of condition-specific falls prevention interventions being designed and evaluated, the implementation of these interventions in clinical practice is proving challenging for clinicians due to insufficient numbers of participants and resources to run group-based programmes [13]. Additionally, the stringent inclusion and exclusion criteria that are regularly associated with explanatory intervention trials reduce their transferability into clinical settings [14]. One potential solution to address these challenges is the development of an evidence-based pragmatic falls prevention programme that can be adapted for use among groups of individuals with different neurological conditions.

While there are differences in the pathophysiology and clinical presentation of stroke, PD and MS [15–18], people with these neurological conditions often present with a number of similar impairments and modifiable falls risk factors. Falls risk factors such as mobility impairments, decreased balance, strength deficits, cognitive dysfunction, depression and fear of falling, in addition to environmental and behavioural falls risk factors, are common across these conditions [19–28]. The mutual modifiable falls risk factors support a mixed-diagnosis intervention approach, as it is likely that the subsequent treatment approaches across the three conditions are similar. Moreover, differences in falls risk factors can exist in individuals with the same diagnosis, for example, not all people with PD present with freezing of gait [29]. Therefore, tailoring of a programme to a person’s unique presentation is necessary for all interventions, independent of diagnosis, and is recommended in international guidelines for falls prevention [30, 31]. Thus, it is anticipated that this model could also be used to develop a programme for people with MS, PD and stroke, and importantly be implemented in primary care. A mixed-diagnosis approach to the design and implementation of interventions could increase the number of eligible participants and services available. Consequently, the development of an intervention for groups with these mixed neurological conditions is timely to address implementation challenges in the community.

To date only one randomised controlled trial (RCT) has investigated the effect of a falls prevention intervention for people with MS, PD and stroke, reporting that an education programme did not reduce falls [32]. An umbrella review investigated the efficacy of condition-specific, exercise-based falls prevention interventions for people with neurological conditions [33]. The review found exercise was effective at reducing falls among people with PD, but insufficient evidence existed to determine their efficacy for people with stroke or MS [33]. The review focused on exercise only, however, a multimodal approach to falls prevention has been recommended to reduce falls risk [19]. Therefore, this umbrella review used a robust methodology to determine the effectiveness of all non-pharmacological and non-surgical falls prevention interventions for people with MS, PD and stroke, and compared and contrasted the effectiveness of interventions across these neurological conditions.

The objectives of this umbrella review were:

1. To summarise the totality of evidence regarding the effectiveness of non-pharmacological and non-surgical falls prevention interventions for people with MS, PD and stroke.
2. To identify commonalities and differences between interventions that are effective at reducing falls for people with MS, PD and stroke to inform the development of an evidence-based intervention that can be tailored for groups with mixed neurological conditions.

## Methods

An umbrella review was conducted to identify and synthesise the results of systematic reviews (with or without meta-analysis) of studies investigating the effectiveness of falls prevention interventions at improving falls outcomes among people with MS, PD and stroke. In lieu of specific guidance for umbrella reviews, this umbrella review was conducted with reference to the Joanna Briggs Institute (JBI) Reviewer’s Manual [34], the relevant sections of the Preferred Reporting Items for Systematic Reviews and Meta-Analysis (PRISMA) guidelines [35, 36], and the key aspects of methods and results of umbrella reviews outlined in the protocol for the Preferred Reporting Items for Overviews of Reviews (PRIOR) guidelines [37].

### Protocol and registration

In compliance with best-practice recommendations to increase transparency and minimise bias, an a priori protocol for this umbrella review was developed [38]. This protocol was registered with the International Prospective Register of Systematic Reviews, PROSPERO, on the 28th April 2020 (CRD42020175409) and was published in an open access repository [39].

### Search strategy

The authors developed a comprehensive search strategy to identify all pertinent research syntheses, both published and unpublished [40]. One reviewer (NO’M) completed searches of the following electronic databases: The Cochrane Database of Systematic Reviews, Joanna Briggs Institute Database of Systematic Reviews and Implementation Reports, Database of Abstracts of Reviews of Effects, PubMed, Embase, EBSCO (Academic Search Complete, AMED, Biomedical Reference Collection, CINAHL, Medline, PsycInfo, SPORTDiscus), Epistemonikos, PEDro and the PROSPERO register. All electronic databases were searched from date of inception to April 2020 (sample search string for the CINAHL is detailed in Appendix1). In addition, the grey literature searches for relevant unpublished systematic reviews encompassed a search of OpenGrey and MedNar. Finally, the reference lists of all included systematic reviews were hand-searched to identify other potentially relevant reviews.

### Inclusion and exclusion criteria

Quantitative systematic reviews (with or without meta-analysis), mixed-methods systematic reviews (quantitative elements only), or pooled analyses and research syntheses investigating the effectiveness of non-pharmacological falls prevention interventions for people with MS, PD and stroke were considered for inclusion in this umbrella review. Reviews published in the English language were included and authors of potentially relevant reviews published in a different language were contacted to ascertain if a copy of the review was available in English. No restriction was placed on year of publication of the review. In instances where a systematic review was an update of a previous review, the most recent version was included and the older version excluded. For the purposes of this umbrella review, a review was classified as an update of a previous version if there were changes pertaining to new data, new methods, or new analyses, but the research question, objectives and inclusion criteria remained similar [41]. In the case of new authors or a different research team updating an existing review, they had to clearly state that their review was an update and acknowledge the work of the authors on the previous edition [41].

The eligibility criteria based on population, intervention, comparator, outcome and study design are outlined below.

#### Population

We included reviews with adult participants (≥18 years) with PD, MS or stroke according to a confirmed diagnostic criterion and reviews with a combination of these conditions. There were no exclusion criteria based on gender, disease duration, disease subtype or functional ability. For the purposes of this umbrella review, there was no exclusions based on the presence of co-morbidities, however, when restrictions based on the presence of co-morbidities were a feature of included reviews, we extracted and recorded this.

#### Intervention

All non-pharmacological and non-surgical falls prevention interventions were included. Any intervention in which a primary or secondary aim was to reduce falls was considered a falls prevention intervention. Given the multifactorial nature of falls, and for inclusivity, there was no exclusion based on intervention content, intervention duration, intervention setting or mode of delivery of intervention.

#### Comparator

In instances where controlled trials were included in the systematic reviews all comparators were considered, including, but not limited to, usual care, enhanced care or waitlist control.

#### Outcomes

The primary outcomes of interest were any falls outcomes, measured as a primary or secondary outcome in the systematic reviews. For this umbrella review, the occurrence of a fall event had to be recorded in order to be classified as a falls outcome. This included, but was not limited to, total number of falls, falls rate, number of fallers, number of recurrent fallers or injurious falls. Of note, reviews in which falls were only measured as adverse outcomes were not included as the aim of the intervention was not to reduce falls. Additionally, reviews in which only laboratory-induced falls were recorded were excluded. Given that there is currently no consensus regarding what constitutes a fall, in addition to the variation in fall definitions present in the literature [42], a pre-determined definition for a fall event was not used in this umbrella review. Instead, all systematic reviews were considered for inclusion regardless of their definition of a fall, but these definitions were extracted and presented to help readers contextualise the results.

Secondary outcomes of interest were those relating to the effectiveness and implementability of interventions. Secondary outcomes were only extracted in instances where falls were measured as a primary outcome and where it was possible to extract this data for the populations and interventions of interest.

#### Study design

Systematic reviews of all study designs investigating falls prevention interventions were considered for inclusion.

Potentially relevant papers were screened for inclusion as a systematic review by two independent reviewers (NO’M and BC) using the JBI Critical Appraisal Checklist for Systematic Reviews and Research Syntheses [34]. Any disagreements between reviewers were resolved through discussion or through consulting a third reviewer until consensus was achieved. Any review that received a ‘No’ response to Items 2,3,4,5 or 8 were excluded [43, 44].

### Study selection

The citations yielded from the searches were exported to the master reference management library Rayyan, where duplicate papers were then removed. The titles and abstracts were screened by two independent reviewers (NO’M and BC) against the eligibility criteria. The authors of potentially relevant protocols and conference abstracts were contacted to determine the full text publication status. Following this, the full texts of potentially relevant reviews were obtained and screened for eligibility by two independent reviewers (NO’M and BC). Any discrepancies between reviewers were resolved through discussion or through consultation with a third reviewer until consensus was achieved.

### Dealing with overlap of primary studies

Overlap of primary studies within included systematic reviews is a challenge exclusive to umbrella reviews. Currently, there is a lack of guidance on how best to manage this occurrence [45]. To maximise comprehensiveness of this umbrella review, we employed a ‘full inclusion scenario’ where all Cochrane reviews and non-Cochrane reviews were included [46]. A list of the primary studies included in each systematic review was assembled and a matrix of evidence table was created to determine the amount of overlap between systematic reviews. To avoid double-counting outcome data the following were decided:

- Any systematic review that contained a relevant primary study that was not in any other systematic review was included so that data were not lost.
- Any systematic review that did not contain any unique primary study (i.e. a primary study not already present in an included review) was excluded to minimise duplication of data.
- In the presence of complete overlap between reviews, the highest quality review, as determined by the A MeaSurement Tool to Assess Systematic Reviews 2 (AMSTAR 2) was included in data synthesis and analysis.
- In cases where there was a complete overlap between reviews and they received the same AMSTAR 2 rating, then the most recently published review was included.
- In the presence of partial overlap, all reviews were included but the degree of overlap was noted and its implications on the findings of this umbrella review were discussed.

### Methodological quality assessment

Two independent reviewers (NO’M and BC) assessed the methodological quality of included reviews using the AMSTAR 2 [47]. In line with recommendations, the critical domains for the AMSTAR 2 were classified as Items 2,4,7,9,11,13 and 15 [47]. The overall score of the AMSTAR 2 was used to rate the quality of each included review as high, moderate, low or critically low [47] [47]..

It has been proposed that the use of the PRISMA reporting guidelines, in addition to a comprehensive, validated quality appraisal tool, facilitates the judgement of not only the methodological quality but also the general quality of reporting of included systematic reviews [48]. Therefore, the full texts of all systematic reviews included in this umbrella review were cross-checked against the PRISMA reporting guidelines checklist [35, 36].

### Data extraction

Data were extracted by one reviewer (NO’M) using the JBI standardised data extraction form for umbrella reviews [34]. The following were also considered key data to answer our research question and were extracted where available: the definition of a fall, the faller classification and the person delivering the intervention. This form was then checked by a second reviewer (MC) to ensure that the extracted data were accurate. Disagreements regarding data extraction were resolved through discussion or by consulting a third reviewer until consensus was achieved.

### Quality of evidence

The quality of evidence was assessed using the Grading of Recommendations Assessments, Development and Evaluation (GRADE) algorithm that has been developed for application to umbrella reviews [49]. The GRADE algorithm was applied to the included systematic reviews by two reviewers (NO’M and MC) to assess the quality of the evidence relating to the following outcomes:

1. Total number of falls – the number of falls recorded by participants throughout the study period
2. Falls rate – the number of falls per person per specific period of time, e.g. falls per person per year
3. Number of fallers - the proportion of participants classified as ‘fallers’ based on the criteria outlined by the researchers e.g. an individual who has one or more falls during the follow-up period

### Data synthesis and analysis

Given the heterogeneity in populations, outcomes and analyses, the findings of included reviews were primarily summarised using a narrative synthesis with the quantitative tabulation of results as appropriate. The primary analyses for this umbrella review were centred on type of neurological condition and type of intervention. Following this, cross-comparison of similarities and differences in the effect of different interventions between the three conditions were reported and discussed. The outcomes of each included systematic review were considered and discussed in the context of their methodological quality, as determined by the AMSTAR 2 and the GRADE algorithm.

### Discordance between reviews

Umbrella reviews provide an opportunity for researchers to address the issue of discordance between reviews and to identify its cause [48]. In instances of discordant reviews in our umbrella review, the algorithm developed by Jadad et al. (1997) was used to resolve and discuss issues of discordance [50].

### Deviations from protocol

To facilitate comparison of intervention effectiveness, we had planned to have a standardised approach to our results by converting the different estimates of effect that we extracted to one common effect measure. However, these analyses were not possible due to the small number of meta-analyses and the heterogeneity between studies in terms of outcomes assessed. We had anticipated that many of our included systematic reviews would include non-randomised trials and as a result had planned to examine the effects of synthesising reviews of varying quality, however, this was not the case as only three systematic reviews included non-randomised trials and so this analysis was not completed.

## Results

**Fig. 1:**
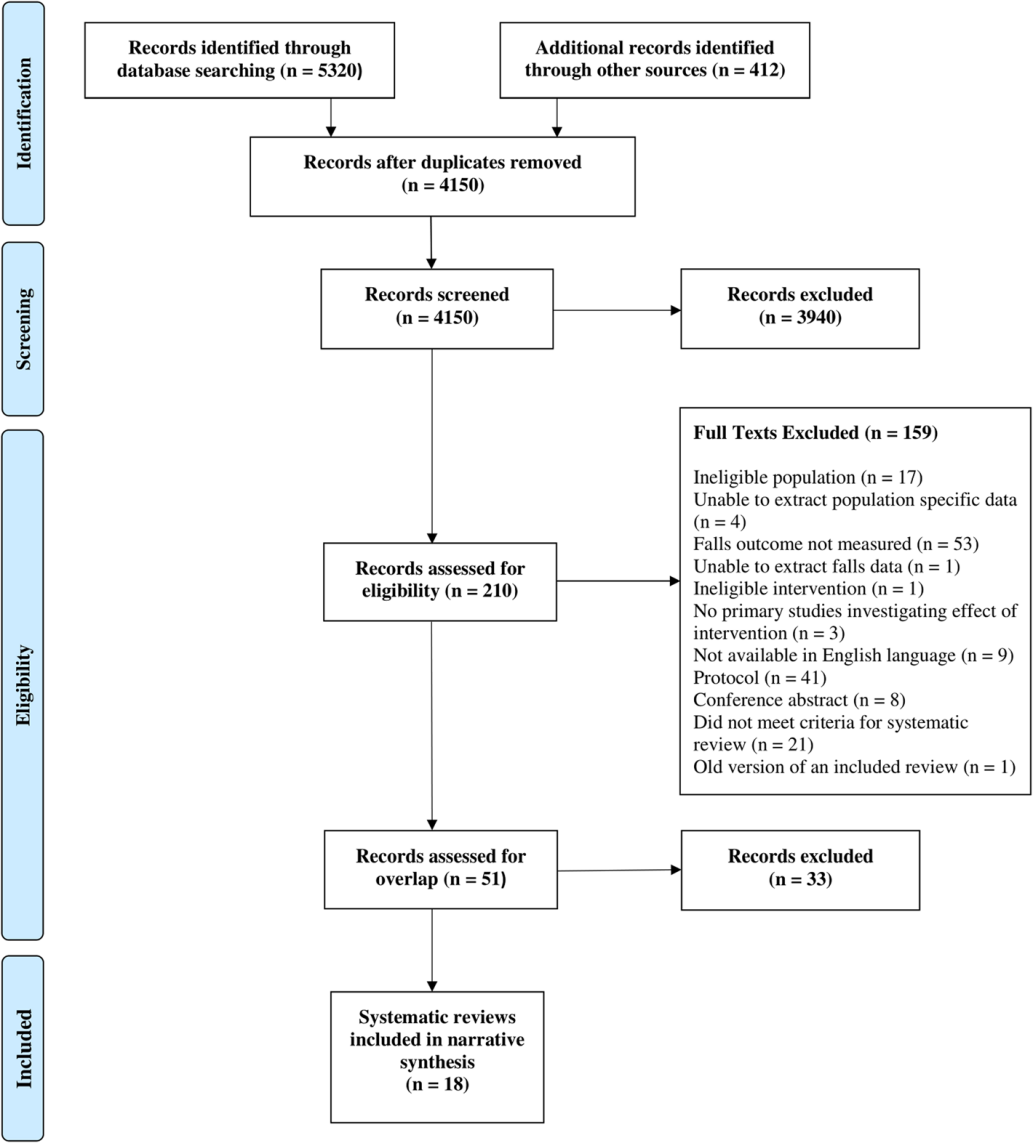
PRISMA flow diagram of review selection

Figure 1 illustrates the study selection process for this umbrella review. A total of 51 reviews were deemed to have met our inclusion criteria. The reasons for exclusion of reviews following full-text screening are reported in Additional file 1. A citation table was produced to establish the degree of overlap between these reviews (see Additional file 2), thus excluding 33 systematic reviews using the predefined criteria for dealing with overlap. Eighteen systematic reviews were included in the final synthesis; three reviews including people with MS [51–53], three reviews including people with stroke [54–56], 11 reviews including people with PD [57–67] and, finally, one review including primary studies with people with stroke and PD [68]. The matrix of evidence table outlining the final citation count and the degree of overlap for these 18 included reviews is presented in Table 1.

**Table 1:** Matrix of evidence table demonstrating degree of overlap and citation count of included systematic reviews

Relevant Primary Studies	SYSTEMATIC REVIEW CITATION																		Citation Count
	Hayes et al. (2019)	Sosnoff & Sung (2015)	Booth et al. (2014)	Denissen et al. (2019)	Pollock et al. (2014)	Batchelor et al. (2010)	Rutz et al. (2020)	Owen et al. (2019)	Rodrigues-Krause et al. (2019)	Winser et al. (2019)	Mak et al. (2017)	Ramazzina et al. (2017)	Song et al. (2017)	Shen et al. (2016)	Tomlinson et al. (2014)	Tomlinson et al. (2012a)	Monti et al. (2011)	Winser et al. (2018)	
Multiple Sclerosis																			
Carling et al. (2017)	▲																		1
Cattaneo et al. (2007)	▲	▲																	2
Cattaneo etal. (2016)	▲																		1
Coote et al. (2013)	▲	▲																	2
Esnouf et al. (2010)	▲	▲																	2
Gandolfi et al. (2015)	▲	▲																	2
Hoang et al. (2016)	▲																		1
Lennon (2013)	▲																		1
Prosperini et al. (2013)	▲	▲																	2
Sosnoff et al. (2014)	▲																		1
Sosnoff et al. (2015)	▲	▲																	2
Stephens et al. (2001)	▲																		1
Taylor et al. (2014)	▲																		1
Kramer et al. (2014)		▲																	1
Nilsagård et al. (2014)		▲																	1
Taylor & Griffin (2015)		▲																	1
Nilsagård et al. (2013)			▲																1
Stroke																			
Ada et al. (2013)				▲															1
Andrade et al. (2017)				▲															1
Batchelor et al. (2012)				▲	▲														2
Dean et al. (2010)				▲															1
Dean et al. (2012)				▲															1
Drummond et al. (2012)				▲															1
Green et al. (2002)				▲		▲													2
Harran et al. (2010)				▲															1
Holmgren et al. (2010)				▲															1
Lau et al. (2010)				▲															1
Mansfield et al. (2018)				▲															1
Marigold et al. (2005)				▲															1
Morone et al. (2016)				▲															1
Taylor-Pillae et al. (2014)				▲														▲	2
Barecca et al. (2004)						▲													1
Cheng et al. (2001)					▲	▲													2
Mead et al. (2007)					▲														1
Widén Holmqvist et al. (1998)						▲													1
Von Koch et al. (2000)						▲													1
Von Koch et al. (2001)						▲													1
Thorsén et al. (2005)						▲													1
Bernhardt et al. (2008)						▲													1
Rossi et al. (1990)						▲													1
Sato et al. (2003)						▲													1
Parkinson’s Disease																			
Li et al. (2012)											▲			▲	▲			▲	4
McGinley et al. (2012)															▲				1
Smania et al. (2010)														▲	▲				2
Ashburn et al. (2007)														▲		▲	▲		3
Goodwin et al. (2009)																▲			1
Marjama-Lyons et al. (2002)																▲			1
Meek et al. (2010)																▲			1
Nieuwboer et al. (2007)														▲		▲			2
Protas et al. (2005)																▲	▲		2
Purchas et al. (2007)																▲			1
Canning et al. (2012)														▲					1
Goodwin et al. (2011)														▲					1
Gao et al. (2014)											▲		▲	▲				▲	4
Morris et al. (2015)								▲			▲			▲					3
Shen & Mak (2015)											▲	▲							2
Shen & Mak (2014)											▲	▲							2
Canning et al. (2015)								▲			▲								2
Sparrow et al. (2016)											▲								1
Li et al. (2014)											▲								1
McKee & Hackney (2013)									▲										1
Farag et al. (2016)										▲									1
Volpe et al. (2014)												▲							1
Morris et al. (2017)								▲											1
Li et al. (2015)													▲						1
Loftus et al. (2014)													▲						1
Gladfelter et al. (2011)																		▲	1
Martin et al. (2015)							▲												1
Paul et al. (2014)												▲							1
Shen & Mak (2012)												▲							1
Canning et al. (2009)																	▲		1
Cakit et al. (2007)																	▲		1
Ledger et al. (2008)																	▲		1
Relevant Citations Count	13	9	1	14	3	10	1	3	1	1	8	5	3	8	3	7	5	4	

### Characteristics of included systematic reviews

Specific details regarding the characteristics of the 18 included reviews are presented in Additional file 3. The included systematic reviews were published between 2010 and 2020. Of those systematic reviews, nine conducted meta-analyses [51, 53–56, 63, 64, 66, 68], however, only six of those reviews conducted a meta-analysis on a falls outcome [51, 54, 56, 63, 64, 68]. The remaining 12 presented their findings on falls outcomes through a narrative synthesis. Falls data were extracted from RCTs only in 15 included reviews, a combination of RCTs and non-randomised studies of intervention (NRSIs) in two reviews, and NRSIs only in one systematic review. The included systematic reviews comprised of primary studies conducted between 1990 and 2018. 17, 24 and 32 primary studies informed the falls outcomes of systematic reviews including people with MS, people with stroke and people with PD, respectively. Despite the exclusion of 33 systematic reviews, some overlap of primary studies across included reviews remained, as demonstrated in Table 1.

### Methodological quality

**Fig. 2:**
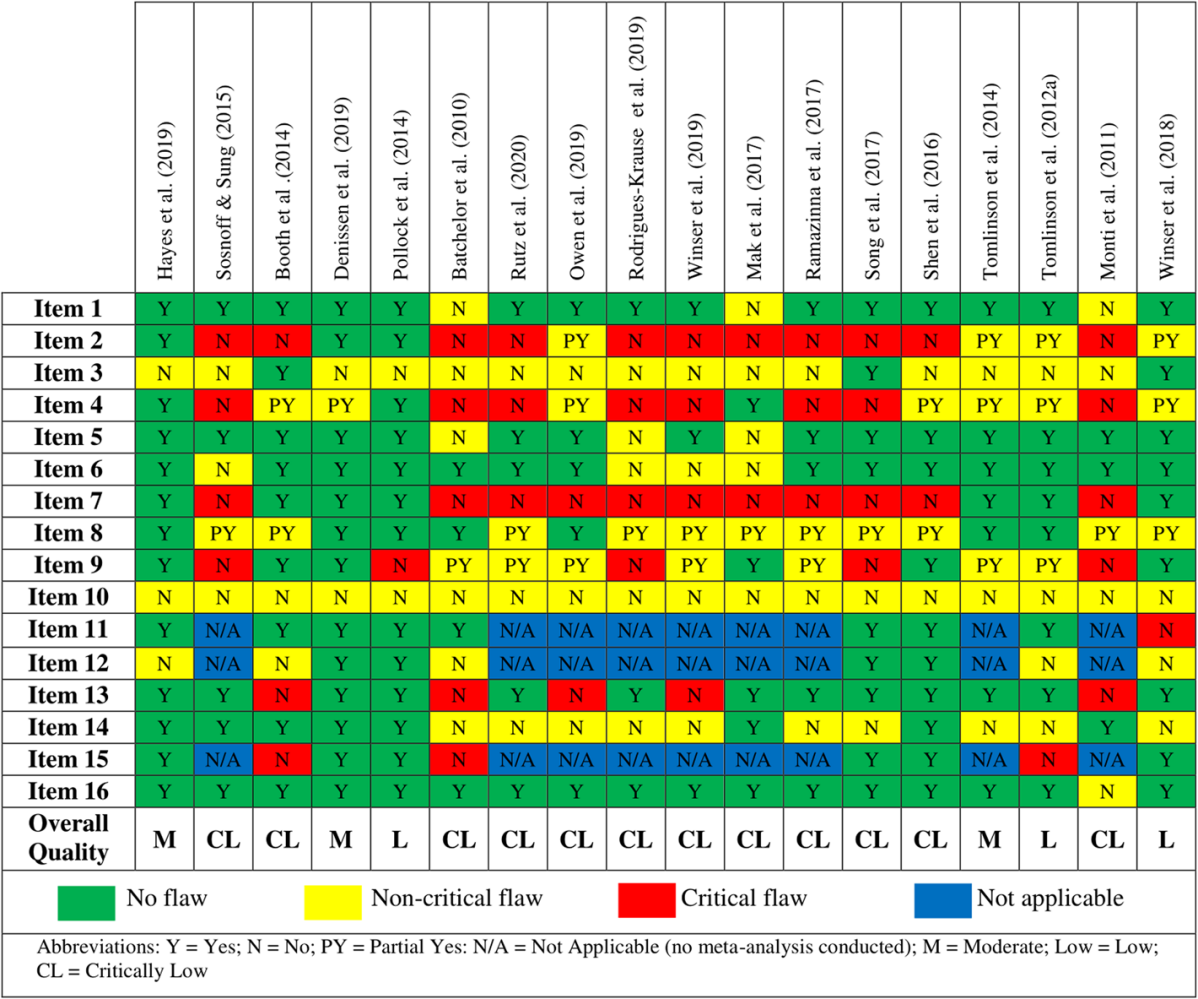
AMSTAR 2 ratings of included systematic reviews

The methodological quality of included systematic reviews is presented in Fig. 2. The quality of included reviews ranged from critically low to moderate, with no included review rated as high quality. The majority of reviews (*n* = 12) were rated as critically low [52, 53, 56–64, 67], with three systematic reviews rated as low [55, 66, 68] and three rated as moderate [51, 54, 65]. None of the included systematic reviews reported the sources of funding for included primary studies. The majority of those rated low or critically low were deemed to have critical flaws relating to a priori protocol development, a comprehensive search strategy, and the listing and justification for exclusions.

### Reporting quality

In general, the reporting across included reviews was relatively complete, however, as shown in Table 2, there were some reporting flaws identified. Firstly, nine of the included reviews did not identify the paper as a systematic review and/or meta-analysis in the title and 11 of the reviews did not register an a priori protocol. This umbrella review also found inadequate reporting of the search strategy, data items, summary measures, results of individual studies and funding.

**Table 2:** Results of Preferred Reporting Items for Systematic Reviews and Meta-Analyses (PRISMA) for included reviews

		Systematic Review Citation																	
Section/ Topic	Items	Hayes et al. (2019)	Sosnoff & Sung (2015)	Booth et al. (2014)	Denissen et al. (2019)	Pollock et al. (2014)	Batchelor et al. (2010)	Rutz et al. (2020)	Owen et al. (2019)	Rodrigues-Krause et al. (2019)	Winser et al. (2019)	Mak et al. (2017)	Ramazzina et al. (2017)	Song et al. (2017)	Shen et al. (2016)	Tomlinson et al. (2014)	Tomlinson et al. (2012a)	Monti et al. (2011)	Winser et al. (2018)
Title																			
	1. Title	N	N	Y	N	N	Y	Y	Y	N	Y	N	Y	Y	Y	N	N	N	Y
Abstract																			
	2. Structured summary	Y	N	Y	Y	Y	Y	Y	Y	Y	Y	N	N	Y	N	Y	Y	Y	Y
Introduction																			
	3. Rationale	Y	Y	Y	Y	Y	Y	Y	Y	Y	Y	Y	Y	Y	Y	Y	Y	Y	Y
	4. Objectives	Y	Y	Y	Y	Y	Y	Y	Y	Y	Y	Y	Y	Y	Y	Y	Y	Y	Y
Methods																			
	5. Protocol and registration	Y	N	N	Y	Y	N	N	Y	N	N	N	N	N	N	Y	Y	N	Y
	6. Eligibility criteria	Y	Y	Y	Y	Y	Y	Y	Y	Y	Y	Y	Y	Y	Y	Y	Y	Y	Y
	7. Information sources	Y	Y	Y	Y	Y	Y	Y	Y	Y	Y	Y	Y	Y	Y	Y	Y	Y	Y
	8. Search	Y	N	N	Y	Y	Y	N	Y	N	Y	N	N	N	N	Y	Y	N	Y
	9. Study selection	Y	Y	Y	Y	Y	Y	Y	Y	N	Y	N	Y	Y	Y	Y	Y	Y	Y
	10. Data collection process	Y	N	Y	Y	Y	Y	N	Y	N	N	N	Y	Y	Y	Y	Y	Y	Y
	11. Data items	Y	N	N	Y	Y	N	N	PY	N	Y	N	Y	Y	Y	Y	Y	Y	Y
	12. Risk of bias in individual studies	Y	Y	Y	Y	Y	Y	Y	Y	Y	Y	Y	Y	Y	Y	Y	Y	Y	Y
	13. Summary measures	Y	N	Y	Y	Y	Y	N	N	N	N	N	N	Y	Y	N	Y	N	Y
	14. Synthesis of results	Y	N	Y	Y	Y	Y	N	N	N	N	N	N	Y	Y	N	Y	N	Y
	15. Risk of bias across studies	Y	N	N	Y	Y	N	Y	N	N	N	N	N	Y	Y	N	N	N	Y
	16. Additional analysis	Y	N	N	Y	Y	N	N	N	N	N	N	N	N	Y	N	Y	N	N
Results																			
	17. Study selection	Y	Y	PY	Y	Y	Y	PY	Y	Y	Y	PY	PY	Y	Y	Y	Y	Y	Y
	18. Study characteristics	Y	PY	PY	Y	Y	Y	PY	Y	PY	Y	Y	PY	PY	Y	Y	Y	PY	Y
	19. Risk of bias within studies	Y	Y	Y	Y	Y	Y	PY	Y	Y	Y	PY	Y	Y	Y	Y	Y	PY	Y
	20. Results of individual studies	Y	N	Y	Y	Y	Y	N	N	N	Y	N	N	Y	Y	N	Y	N	Y
	21. Synthesis of results	Y	N	Y	Y	Y	Y	N	N	N	N	N	N	Y	Y	N	Y	N	Y
	22. Risk of bias across studies	Y	N	N	Y	Y	N	Y	N	N	N	N	N	Y	Y	N	N	N	Y
	23. Additional analysis	Y	N	N	Y	Y	N	N	N	N	N	N	N	N	Y	N	Y	N	N
Discussion																			
	24. Summary of evidence	Y	Y	Y	Y	Y	Y	Y	Y	Y	Y	Y	Y	Y	Y	Y	Y	Y	Y
	25. Limitations	Y	PY	Y	Y	Y	Y	Y	PY	PY	Y	PY	Y	Y	Y	Y	Y	PY	Y
	26. Conclusions	Y	Y	Y	Y	Y	Y	Y	Y	Y	Y	Y	Y	Y	Y	Y	Y	Y	Y
Funding																			
	27. Funding	PY	PY	PY	N	PY	PY	N	PY	PY	N	N	N	PY	Y	PY	PY	N	Y

Abbreviations: Y = Yes (a complete report); PY = Partial Yes (a partially compliant report); N = No (no report)

### Participant characteristics

The number of participants in included reviews ranged from 21 to 1358. The mean age of participants was lower in systematic reviews of people with MS (range 36–63 years in included primary studies) compared to systematic reviews of people with PD and stroke, where the mean age of participants was greater than 60 years in the vast majority of included primary studies. Disease severity and functional ability were not regularly reported in included reviews but, of note, when it was participants generally had a relatively low disease severity and high functional level. Specific details regarding the participants in each included systematic review are presented in Table 3 and Additional file 3.

**Table 3:** Summary of evidence for included systematic reviews

Review details	Aim	Participant information	Intervention details	Summary of findings of review	Certainty of evidence (GRADE)
Citation details: Hayes et al. (2017) Number of relevant primary studies: 13 AMSTAR 2 rating: Moderate Neurological condition: MS	To evaluate the effectiveness of interventions to reduce falls in people with MS, specifically to compare falls prevention interventions to controls and to compare different types of falls prevention interventions.	*N* = 839 (range: 12–177) Female participants range: 59–98% Mean age = 52 years (range: 36–62 years) Participants in the majority of RCTs included people with mild to moderate severity of MS.	Group-based exercise session or individualised HEP × 1 RCT One-to-one motor and sensory rehabilitation or motor rehabilitation × 1 RCT Balance treatment × 1 RCT Group-based exercise circuit class or one-to-one physiotherapy or yoga classes × 1 RCT FES × 1 RCT Supervised sensory integration balance training × 1 RCT Interactive exergames × 1 RCT Group-based exercise × 1 RCT Wii Fit Plus balance games × 1 RCT Progressive HEP × 1 RCT Exercise or education or combined exercise and education × 1 RCT Activity Through Movement × 1 RCT FES and core stability exercises × 1 RCT	Falls rate: There was no significant effect of exercise compared to control on falls rate (RaR 0.68, 95% CI 0.43 to 1.06).	Moderate
				There was no significant effect of FES compared to exercise on falls rate (RaR 0.91, 95% CI 0.78 to 1.06).	Low
				Number of fallers: There was no significant effect of exercise on the number of fallers post-intervention (RR 0.85, 95% CI 0.51 to 1.43).	Moderate
				There was no evidence of an effect of education-based interventions on number of fallers (RR 0.83, 95% CI 0.40 to 1.76).	Moderate
				There was no evidence of an effect of multicomponent interventions on number of fallers (RR 0.30, 95% CI 0.04 to 2.20).	Moderate
Citation details: Sosnoff & Sung (2015) Number of relevant primary studies: 9 AMSTAR 2 rating: Critically low Neurological condition: MS	To review the effects of falls prevention interventions on falls incidence among people with MS and determine characteristics of these programmes that might optimise the reduction of falls.	*N* = 504 (range: 28–111) Percentage of female participants: N/R EDSS median range: 3.0–6.0 (N/R ×3 studies: 1x RCT, 2x NRSIs) Mean age range: 46–63 years	Exercise-based ×7 studies: Motor-sensory rehabilitation or motor rehabilitation × 1 RCT Group physiotherapy or 1-to-1 physiotherapy or yoga × 1 NRSI Wii balance board system training × 1 RCT Exergame training on an unstable platform or single-task exercises on the unstable surface × 1 NRSI Balance exercise targeting core stability, dual tasking and sensory strategies × 1 NRSI Home-based exercise or education or exercise and education × 1 RCT Sensory integration rehabilitation × 1 RCT Technology-based × 2 studies: FES for 12 weeks and exercise with FES for 12 weeks × 1 RCT FES × 1 RCT	Total number of falls: 3x studies reported a significant reduction in the number of falls in the exercise-based intervention groups (1x RCT, 2x NRSIs).	Low
				2x RCTs reported a reduction in total number of falls for groups receiving FES.	Very low
				Number of fallers: 2x RCTs reported that the number of fallers was lower following exercise-based intervention, 1x NRSI reported no difference between groups.	Very low
				Number of recurrent fallers: 1x RCT reported a significantly lower number of recurrent fallers in the exercise-based intervention groups compared to the control group.	Not assessed
				Mean number of falls: 1x NRSI reported that the exercise-based intervention group had a lower mean number of falls than the control group.	Not assessed
Citation details: Booth et al. (2014) Number of relevant primary studies: 1 AMSTAR 2 rating: Critically low Neurological condition: MS	To evaluate whether virtual reality interventions, including interactive gaming systems, are effective at improving balance in adults with impaired balance.	*N* = 80 Percentage of female participants: N/R Age: N/R	Nintendo WiiFit balance exercise programme ×1 RCT	Total number of falls: 1x RCT identified that those in the intervention group experienced less falls (*n* = 10) compared with the control group (*n* = 14) during the study.	Critically low
Citation details: Denissen et al. (2019) Number of relevant primary studies: 14 AMSTAR 2 rating: Moderate Neurological condition: Stroke	To evaluate the effectiveness of interventions aimed at preventing falls in people after stroke.	*N* = 1358 (range: 34–170, median: 91) Mean percentage of female participants: 40% (range: 29–65%) Mean age range: 57 (+/−11) - 79 (+/−8) years	Exercise-based interventions ×8 RCTs: Treadmill training without body weight support plus overground walking × 1 RCT Treadmill walking with harness plus conventional stroke rehabilitation × 1 RCT WEBB programme plus HEP plus advice to increase walking × 1 RCT Physiotherapy treatment × 1 RCT Whole-body vibration × 1 RCT External perturbation training × 1 RCT Exercise programme challenging dynamic balance and emphasising agility and multisensory approach in between × 1 RCT Tai Chi or SilverSneakers × 1 RCT Environment/assistive technology ×3 RCTs: Predischarge home assessment visit × 1 RCT Prescription of single lens distance glasses × 1 RCT Walking training using the I-Walker plus exercises on hand recovery, tone control and improvement of global ability × 1 RCT Other interventions/ procedures × 1 RCT: Active repeated tDCS plus physical rehabilitation × 1 RCT Multifactorial intervention × 1 RCT: Multifactorial, individually-tailored falls prevention programme plus usual care after discharge × 1 RCT Multiple intervention × 1 RCT: HIFE programme plus individualised HEP × 1 RCT	Falls rate: There was a significant reduction in falls rate for the exercise group (RaR 0.72, 95% CI 0.54 to 0.94).	Moderate
				There was no significant reduction in falls rate when comparing a home visit to a predischarge assessment in the hospital setting (RaR 0.85, 95% CI 0.43 to 1.69).	Low
				There was no significant reduction in falls rate when single lens distance vision glasses replaced multifocal glasses (RaR 1.08, 95% CI 0.52 to 2.25).	Low
				There was no significant reduction in falls rate for the I-walker group compared to the control group (RaR 0.56, 95% CI 0.19 to 1.66).	Low
				Number of fallers: When pooled, there was no significant effect of exercise on number of fallers (RR 1.03, 95% CI 0.90 to 1.19).	Moderate
				There was no significant difference in number of fallers between the home visit or hospital assessment groups (RR 1.48, 95% CI 0.71 to 3.09).	Low
				There was no significant reduction in number of fallers when single lens distance vision glasses replaced multifocal glasses (RR 0.74, 95% CI 0.47 to 1.18).	Low
				There was no significant reduction in number of fallers for the I-walker group compared to the control group (RR 0.44, 95% CI 0.16 to 1.22).	Low
				There was a significant reduction in the number of fallers in the active tDCS group compared to the control group (RR 0.30, 95% CI 0.14 to 0.63).	Low
Citation details: Pollock et al. (2014) Number of relevant primary studies: 3 AMSTAR 2 rating: Low Neurological condition: Stroke	To determine the effect of interventions that alter the starting posture on ability to STS independently and to determine the effect of rehabilitation interventions on ability to STS independently.	*N* = 276 (range: 54–156) Percentage of female participants: N/R Mean age range (intervention group): 60 (+/−7) - 72 (+/−10.4) years Mean time since stroke range (intervention group): 21 (+/−8) – 171 days	Repetitive STS training × 1 RCT Falls prevention programme × 1 RCT Endurance and resistance exercises × 1 RCT	Number of fallers: There was no evidence of an effect of intervention on the number of fallers compared to control (OR 0.81, 95% CI 0.35 to 1.87).	Moderate
Citation details: Batchelor et al. (2010) Number of relevant primary studies: 10 (based on 7 RCTs) AMSTAR 2 rating: Critically low Neurological condition: Stroke	To systematically evaluate the effects of any interventions on falls in people after stroke.	*N* = 723 (range: 39–258) Percentage of female participants: N/R ×6 RCTs, 46% × 1 RCT Age = N/R ×5 RCTs, range: 18–90 years × 1 RCT, mean: 74.7 years × 1 RCT Time since stroke range: < 24 h to > 2 years post-stroke	Group STS practice plus usual care × 1 RCT Very early mobilisation plus usual care × 1 RCT Standing symmetry training and STS training plus usual care × 1 RCT Community physiotherapy sessions × 1 RCT Fresnel prisms applied to affected hemi-field plus usual rehabilitation × 1 RCT Sunlight exposure outdoors × 1 RCT Home rehabilitation × 1 RCT	Falls rate: These was no significant effect of exercise on falls rate compared to usual care (RaR 1.22, 95% CI 0.76 to 1.98).	Very low
				The application of fresnel prisms to the affected hemi-field had no significant effect on falls rate.	Very low
				Increased sunlight exposure had no significant effect on falls rate.	Low
				Home rehabilitation with multi-disciplinary outreach service had no significant effect on falls rate.	Very low
				Number of fallers: There was no significant effect of exercise on number of fallers compared to usual care (RR 0.77, 95% CI 0.24 to 2.43).	Low
Citation details: Rutz et al. (2020) Number of relevant primary studies: 1 AMSTAR 2 rating: Critically low Neurological condition: PD	To investigate the evidence for physical interventions for freezing of gait and gait impairments in PD and establish recommendations for clinical practice.	*N* = 21 Percentage of female participants: N/R Age: N/R H&Y range: 2–3 All participants had freezing of gait	HEP with rhythmic auditory cueing and functional walking exercises × 1 RCT	Total number of falls: Intervention did not significantly reduce falls.	Very low
Citation details: Owen et al. (2019) Number of relevant primary studies: 3 AMSTAR 2 rating: Critically low Neurological condition: PD	To identify and review falls self-management interventions for people with PD and, where possible, assess their efficacy for improving patient and caregiver outcomes, quality of life and psychological outcomes.	*N* = 574 (range: 133–231) Percentage of female participants: N/R Mean age range: 67.9 (+/− 9.6) - 71.4 (+/− 8.1) years Majority participants H&Y stage ≤2 (indicating reduced falls risk) × 2 RCTs Range of participants that had fallen in year preceding intervention: 55–78%	Physiotherapy plus education ×3 RCTs	Falls rate: Physiotherapy plus falls self-management education significantly reduced falls rate.	Moderate
				Number of fallers: Physiotherapy plus falls self-management education did not have a significant effect on number of fallers.	Low
				Number of recurrent fallers: No significant effect of intervention on number of recurrent fallers.	Not assessed
Citation details: Rodrigues-Krause et al. (2019) Number of relevant primary studies: 1 AMSTAR 2 rating: Critically low Neurological condition: PD	To review dance as a form of intervention to promote functional and metabolic health in older adults.	*N* = 33 Percentage of female participants: 39% Intervention group mean age = 68.4 +/− 7.7 years Control group mean age = 74.4 +/− 6.5 years	Tango dance × 1 NRSI	Total number of falls: Tango group had reduced number of falls compared to education group.	Very low
				Number of fallers: There was no significant effect of Tango on number of fallers compared to education.	Very low
Citation details: Winser et al. (2019) Number of relevant primary studies: 1 AMSTAR 2 rating: Critically low Neurological condition: PD	To identify evidence evaluating the cost-effectiveness of physiotherapy treatment techniques for people with neurological disorders.	*N* = 231 Percentage of female participants: 41.6% Mean age: 70.7 years	Group based exercise classes with home visits from physical therapist and provision of standard fall prevention booklet × 1 RCT	Mean number of falls: Lower mean number of falls in intervention group (4.106 falls) than control group (7.053 falls).	Not assessed
Citation details: Mak et al. (2017) Number of relevant primary studies: 8 (based on 6 RCTs) AMSTAR 2 rating: Critically low Neurological condition: PD	To investigate the long-term effects of exercise and physical therapy in people with PD.	*N* = 790 (range: 23–195) Percentage of female participants: N/R Age range: 40–89 years H&Y stage range: 1–4	Balance training ×4 RCTs: Progressive strengthening, balance, cueing for FOG and fall prevention advice × 1 RCTs Mobility and balance training with movement strategies and fall prevention advice or progressive resistance training and fall prevention advice × 1 RCT Technology-assisted balance and mobility training × 1 RCT Balance and mobility training × 1 RCT Complementary exercises × 2 RCTs: Tai Chi × 1 RCT Tai Chi or progressive strength training × 1 RCT	Falls rate: Balance training significantly reduced falls rate.	Low
				Tai Chi significantly reduced falls rate.	Low
Citation details: Ramazzina et al. (2017) Number of relevant primary studies: 5 (based on 4 RCTs) AMSTAR 2 rating: Critically low Neurological condition: PD	To assess the effectiveness of resistance training on muscle strength improvement.	*N* = 154 (range: 29–51) Percentage of female participants: N/R Age: N/R H&Y range: 1.5–3	Strength training × 4 RCTs: Using pneumatic resistance equipment × 1 RCT Using dynamometers and leg-press machines, in addition to rowing exercises, repetitive step on a 6-in. curb, and weighted walking × 1 RCT Using dynamometers and leg-press machines, in addition to rowing exercises, repetitive step on a 6-in. curb, and weighted walking plus home training × 1 RCT Hydrotherapy with perturbation-based balance and strength training × 1 RCT	Total number of falls: 1x RCT reported a significant reduction with strength training (hydrotherapy), 3x RCTs reported no significant reduction in number of falls with strength training.	Low
Citation details: Song et al. (2017) Number of relevant primary studies: 3 AMSTAR 2 rating: Critically low Neurological condition: PD	To investigate the effects of Tai Chi/Qigong on motor and non-motor function, and quality of life in people with PD.	*N* = 305 (range: 34–195) Percentage of female participants: 38% Mean age range: 66–69.5 years	Tai Chi × 2 RCTs Qigong × 1 NRSI	Total number of falls: Tai Chi significantly reduced number of falls compared to control (ES − 0.403, 95% CI − 0.677 to − 0.129).	Moderate
				1x NRSI reported Qigong reduced number of falls.	Low
Citation details: Shen et al. (2016) Number of relevant primary studies: 8 AMSTAR 2 rating: Critically low Neurological condition: PD	To examine the effects of exercise on improving balance and gait ability and reducing falls among people with PD over the short-term and long-term.	*N* = 925 (range: 64–231) Percentage of female participants: 38% Age range: 61.6 (+/− 8) - 72.2 (+/− 9.2) years H&Y range: 1–4	Balance, gait, strength, other exercises × 2 RCTs Gait × 1 RCT Balance × 2 RCTs Balance, strength × 1 RCT Strength × 1 RCT Balance and gait × 2 RCTs	Falls rate: The fall rate showed a significant overall reduction over the short-term with exercise training (RaR 0.485, 95% CI 0.329 to 0.715).	Moderate
				The fall rate showed a significant overall reduction over the long-term with exercise training RaR 0.413,95% CI 0.270 to 0.630).	Moderate
				Numbers of fallers: The number of fallers did not decrease significantly over the short-term with exercise training (RR 0.939, 95% CI 0.822 to 1.072).	Moderate
				The number of fallers did not decrease significantly over the long-term with exercise training (RR 0.787, 95% CI 0.605 to 1.024).	Moderate
Citation details: Tomlinson et al. (2014) Number of relevant primary studies: 3 AMSTAR 2 rating: Moderate Neurological condition: PD	To assess the effectiveness of one physiotherapy intervention compared with a second approach in people with PD.	*N* = 469 (range: 64–210) Percentage of female participants: 36% Mean age range: 67.3–69 years Mean disease duration range: 6.7–10.4 years	Tai Chi or resistance training × 1 RCT Movement strategy training and individualised home practice session and weekly structured falls risk education and a single home visit or progressive strength training and individualised HEP and once weekly structured falls risk education and a single home visit × 1 RCT Balance training × 1 RCT	Total number of falls: 1x RCT found number of falls were significantly reduced during the intervention period in the progressive strength training group, 2x RCTs found no significant effect on number of falls.	Low
				Time to first fall: No significant difference in time to first fall between the progressive strength training and the movement strategy training arms × 1 RCT.	Not assessed
Citation details: Tomlinson et al. (2012a) Number of relevant primary studies: 7 AMSTAR 2 rating: Low Neurological condition: PD	To assess the effectiveness of physiotherapy intervention compared with no intervention or placebo in patients with PD.	*N* = 532 (range: 18–153) Percentage of female participants: 38% female across 6x RCTs (N/R × 1 RCT, male participants only × 1 RCT) Mean age range: 63.4–73.7 years (N/R × 1 RCT) Mean H&Y range: 2–3.14 (N/R × 2 RCTs) Mean disease duration range: 4.7–9.1 years (N/R × 2 RCTs)	Exercise × 3 RCT Tai Chi × 2 RCTs Cueing × 1 RCT Treadmill training × 1 RCT	Total number of falls: 1x RCT reported a significant reduction in number of falls for the Tai Chi compared with no intervention, 5x RCTs reported no significant effect of exercise-based intervention on number of falls.	Low
				There was no significant effect of cueing intervention on number of falls.	Low
Citation details: Monti et al. (2011) Number of relevant primary studies: 5 AMSTAR 2 rating: Critically low Neurological condition: PD	To research the effectiveness of physiotherapy intervention on the prevention of falls among people with PD.	*N* = 456 (range: 18–230) Percentage of female participants: N/R Mean age range: 71.8 +/− 6.4 years to 72.5 years (mean age N/R × 3 RCTs) Age range: 44–91 years (age range N/R × 4 RCTs)	Exercise-based interventions × 4 RCTs: Exercises, cueing with the integration in the ADL plus received a booklet with advice for the prevention of falls × 1 RCT. Treadmill walking, exercise to increase ROM and stretching exercises × 1 RCT Exercises to strengthen the muscles of the legs, to increase the ROM, for the equilibrium, for walking outdoor × 1 RCT Treadmill walking × 1 RCT Cueing intervention × 1 RCT	Total number of falls: 4x RCTs report a reduction in the number of fall episodes for the exercise-based intervention groups.	Low
				The cueing intervention group had a decrease in the number of falls in the ADL × 1 RCT.	Very low
Citation details: Winser et al. (2018) Number of relevant primary studies: 4 AMSTAR 2 rating: Low Neurological condition: Stroke and PD	To determine whether Tai Chi training improves balance and reduces falls incidence when compared to control conditions of either active treatment or no treatment in people with neurological diseases.	PD: *N* = 288 (range 17–195) Percentage of female participants: 36% Age: 72 +/− 8.5 years (N/R × 2 RCTs) Stroke: *N* = 145 Percentage of female participants: 47% Age: 69.9 +/− 10 years	PD: Tai Chi × 3 RCTs Stroke: Tai Chi × 1 RCT	Total number of falls (PD): There was a statistically significant effect of Tai Chi compared with active therapies on total number of falls (OR 0.47, 95% CI 0.29 to 0.77).	Moderate
				There was a statistically significant effect of Tai Chi compared with no treatment on total number of falls (OR 0.29, 95% CI 0.11 to 0.79).	Low
				Total number of falls (Stroke): There was a statistically significant effect of Tai Chi compared with active therapies on total number of falls (OR 0.21, 95% CI 0.09 to 0.48).	Low

Abbreviations: GRADE = Grading of Recommendations, Assessment, Development and Evaluations; AMSTAR 2 = A MeaSurement Tool to Assess Systematic Reviews 2; MS = Multiple Sclerosis; RCT = Randomised Controlled Trial; HEP = Home Exercise Programme; FES = Functional Electrical Stimulation; RaR = Rate Ratio; CI = Confidence Interval; RR = Risk Ratio; N/R = Not Reported; EDSS = Expanded Disability Status Scale; NRSI = Non-Randomised Study of Intervention; WEBB = Weight-bearing Exercise for Better Balance; tDCS = transcranial Direct Current Stimulation; HIFE = High-Intensity Functional Exercise; STS = Sit-To-Stand; OR = Odds Ratio; PD = Parkinson’s Disease; H&Y = Hoehn and Yahr; FOG = Freezing Of Gait; ES = Effect Size; ADL = Activities of Daily Living; ROM = Range of Motion

### Critical appraisal of primary studies

As presented in Additional file 3, a variety of different critical appraisal instruments were used to assess the methodological quality of included primary studies. The methodological quality of primary studies was varied and was noted as a limitation in the majority of included systematic reviews. A detailed summary of the critical appraisal of the primary studies is outlined in Additional file 3.

### Person delivering the intervention and intervention setting

The person delivering the intervention and intervention setting were not regularly reported in included systematic reviews. Of those presenting the person delivering the intervention, physiotherapists were most common, other disciplines included occupational therapists, yoga instructors, Tai Chi instructors, optometrists and multi-disciplinary teams. Interventions were primarily delivered in the community or the participants’ homes, but other settings including rehabilitation centres, hospitals and acute care were also reported.

### Intervention characteristics

Exercise-based interventions were the most common across all three conditions, included in a total of 15 systematic reviews. Tai Chi and treadmill walking interventions were assessed among people with PD and stroke, but not MS. Dance-based exercise interventions were only investigated among people with PD. Technology-based interventions and multicomponent interventions were reported across more than condition, with all other reported interventions investigated amongst only one. There was substantial variation across reviews with respect to the reporting of intervention details. Specific intervention characteristics including, where reported, content, dose and duration are presented for included reviews in Table 3 and Additional file 3.

### Exercise-based interventions

This umbrella review identified low to moderate quality evidence for exercise-based interventions among people with PD, with seven out of ten reviews reporting a significant effect of intervention on the recorded falls outcome [59–61, 63, 64, 67, 68]. The remaining three reviews for PD identified mixed results regarding the effectiveness of intervention across included primary studies [62, 65, 66]. The effectiveness of exercise-based interventions varied across reviews for people after stroke, with two reviews reporting significant improvements in falls outcomes [54, 68] and two reporting no evidence of effect [55, 56]. However, the two that showed effect were of higher methodological quality and provided low to moderate quality evidence for the effectiveness of exercise at improving falls outcomes for people after stroke. The evidence for exercise among people with MS also differed across reviews with one reporting no effect [51] and two reporting an improvement in falls outcomes [52, 53], but the most recently published review that had the highest methodological quality and the largest number of primary studies informing outcomes reported no evidence of effect of exercise-based interventions on falls rate or number of fallers.

### Technology-based interventions

Technology-based interventions were investigated among people with MS and stroke. The evidence regarding the effectiveness of these interventions varied across reviews for people with MS, with one review of moderate methodological quality reporting no significant effect of functional electrical stimulation (FES) [51] compared to another of critically low methodological quality that reported FES led to a reduction in total number of falls and number of fallers [52]. Technology-based interventions were investigated in one systematic review for people with stroke, providing low quality evidence for transcranial direct stimulation to reduce number of fallers [54].

### Multicomponent and multifactorial interventions

Multicomponent interventions were investigated for people with MS and PD in one systematic review each [51, 58]. No significant effect of multicomponent interventions was identified for people with MS or PD on number of fallers. However, there was moderate evidence identified for the effectiveness of a multicomponent intervention comprising of physiotherapy and falls-self management education at reducing falls rate among people with PD [58].

Two systematic reviews for people with stroke included a multifactorial intervention [54, 55], however, this intervention was primarily comprised of exercises and so was included in the analyses for exercise-based interventions in these reviews.

### Education-based interventions

Education interventions were only reported in one systematic review of moderate methodological quality among people with MS [51]. This review concluded that there was no significant effect of education-only interventions on number of fallers but this outcome was only informed by one primary study.

### Environment/assistive technologies

Environment/assistive technologies were assessed among people with stroke in two reviews ranging from critically low to moderate methodological quality [54, 56]. These reviews identified no significant effect of intervention on number of fallers or falls rate.

### Cueing interventions

Cueing interventions for people with PD were assessed in three systematic reviews of low to critically low methodological quality [57, 66, 67]. One of these reviews provided very low quality evidence for the effectiveness of cueing at reducing falls [67]. The two remaining systematic reviews reported that there was no significant effect of cueing interventions on total number of falls [57, 66].

### Interventions to improve bone mineral density

One systematic review of critically low methodological quality investigating the effect of interventions to improve bone mineral density for people with stroke reported that increased sunlight exposure had no significant effect on falls rate [56].

### Models of stroke care interventions

One systematic review of critically low methodological quality investigating the effect of different models of stroke care on falls outcomes for people with stroke concluded that home rehabilitation with multidisciplinary team outreach had no significant effect on falls rate [56].

### GRADE quality of evidence

The quality of evidence relating to total number of falls, falls rate and number of fallers for each outcome is outlined in the summary of evidence table (see Table 3) and the application of the algorithm to each of these is outlined in Additional file 4.

### Discordance between reviews

Inconsistencies in findings regarding the effectiveness of exercise-based interventions and FES were identified between reviews for people with MS. A potential reason for this difference in findings may be the different selection criteria of the two systematic reviews [50], with Hayes et al. (2019) only including RCTs [51] and Sosnoff and Sung (2015) including both RCTs and NRSIs [52].

Variations in findings regarding the efficacy of exercise-based interventions on falls rate for people with stroke were also identified [54, 56], which may be explained by the differences in studies informing outcome, with only one primary study included in both reviews.

### Secondary outcomes

There were a broad range of secondary outcomes assessed in included systematic reviews including, but not limited to, gait, quality of life, balance, cognition and fatigue. These were assessed with a number of different outcome measures. The effectiveness of falls prevention interventions at improving these outcomes varied across reviews. Of note, very few included reviews provided data regarding adverse events or cost-effectiveness of interventions. The findings regarding secondary outcomes are presented in Additional file 3.

## Discussion

This comprehensive umbrella review included 18 systematic reviews representing 73 unique primary studies investigating the effectiveness of falls prevention interventions at reducing fall events among people with MS, PD and stroke. This review identified low to moderate quality evidence for the effectiveness of exercise-based interventions at reducing falls among people with PD, both over the short-term and long-term. Reviews investigating the effectiveness of exercise-based interventions for people with MS and stroke yielded conflicting results, however, the best available evidence suggests that exercise is effective at reducing falls among people with stroke but no evidence of effect was identified for people with MS. All other types of intervention are relatively under-researched, with insufficient evidence available to draw definitive conclusions regarding their effectiveness.

The large number of systematic reviews that investigated the effectiveness of falls prevention interventions for people with MS, PD and stroke suggest that this field is well researched, particularly among people with PD and stroke. However, this umbrella review has demonstrated that this proliferation in systematic reviews is not reflective of an increase in primary studies but rather that the findings across all of these reviews are largely based on the same small number of trials. The quality and value of a systematic review is largely dependent on the number and methodological quality of included primary studies [69]. Therefore, the inclusion of the same primary studies across many systematic reviews results in the same limitations, such as heterogeneity in methods and outcomes, small sample sizes, and high risk of bias, being reported in newer systematic reviews thus not contributing to advancements in the research field and evidence-based practice. This umbrella review has identified a critical lack of high-quality trials investigating falls prevention interventions for people with MS, PD and stroke, prohibiting researchers and clinicians from drawing firm conclusions regarding their effectiveness.

The finding that exercise is effective at reducing falls among people with PD is consistent with that of Lai et al. (2019) who investigated the effectiveness of exercise-only falls prevention interventions for people with neurological diseases [33]. The inclusion of additional systematic reviews in our umbrella review, along with the application of the GRADE algorithm (showing low to moderate quality evidence), increases the certainty of this finding. However, a difference was noted between the findings of this umbrella review and that of Lai et al. (2019) with respect to the effectiveness of exercise-based interventions for people with stroke. Lai et al. (2019) included two systematic reviews for people with stroke, neither of which found effect. However, two recent systematic reviews identified as part of this umbrella review, neither of which were included in the paper by Lai et al. (2019), found exercise-based interventions significantly improved falls outcomes for people with stroke [54, 68]. Therefore, the highest quality research and best available evidence for people with stroke, as demonstrated by the AMSTAR 2 and GRADE algorithm, suggests that exercise can reduce falls for these individuals. The evidence regarding the efficacy of exercise-based interventions for people with MS was the least comprehensive, with the smallest amount of primary studies and systematic reviews informing outcomes. The systematic review of the highest methodological quality for people with MS did not find any evidence of effect for exercise-based interventions on falls outcomes but the authors noted that there was an absence of high-quality RCTs in this field meaning that the findings of this review are inconclusive [51]. An umbrella review investigating rehabilitation for people with MS identified high-quality evidence for exercise to improve mobility, muscle strength and fatigue, three common modifiable falls risk factors for people with MS [70]. Consequently, despite the lack of evidence supporting the use of exercise-based interventions for people with MS, a strong theoretical basis for their use remains. There is conclusive evidence regarding the effectiveness of exercise at reducing falls among older adults [71, 72], a much further advanced research field than falls prevention for people with neurological diseases and therefore, further high-quality research is required to determine its effectiveness for people with MS, PD and stroke.

Exercise-based interventions were the most commonly investigated intervention type for all three conditions, supporting the feasibility of a mixed-diagnosis intervention. However, a noticeable difference was identified between the three conditions in terms of the content of these exercise-based interventions. The majority of interventions for people with PD comprised of mixed training, usually involving some combination of balance, strength and functional or gait exercises. This is in contrast to interventions for people with MS and stroke which were primarily focused on one specific training, most commonly balance training or functional movement. Three of the main modifiable falls risk factors for PD, MS and stroke are strength deficits, balance dysfunction and gait impairments and, therefore, it can be hypothesised that the most effective method of reducing falls risk for these individuals would be through a mixed exercise programme, as demonstrated in falls research for older adults [71]. This umbrella review identified low to moderate quality evidence for the efficacy of Tai Chi interventions at improving falls outcomes for people with PD and stroke but this intervention does not have reviews with falls outcomes for people with MS. Another difference was identified between conditions with respect to intervention dosage. A recent review in older adults concluded that interventions consisting of balance training and functional exercises that involved a total weekly dose of three or more hours were most effective at reducing falls rate [71]. Intervention dose was not routinely reported in included reviews but where it was, only four interventions for people with MS and seven for people with stroke achieved this dosage. While formal statistical analyses could not be completed, the findings of this review would suggest that exercise-based interventions that involved mixed training and had a higher intervention dose led to improvements in falls outcomes. Therefore, future trials investigating the efficacy of mixed exercise interventions of sufficient dosage for people with MS, PD and stroke are recommended.

Falls are widely accepted as having a range of causes and, therefore, a surprising finding of this review was the lack of multicomponent or multifactorial interventions that have been investigated for people with MS, PD and stroke. The majority of interventions included in this review were developed with the aim of targeting the physiological falls risk factors, however, psychosocial, environmental and behavioural factors (that are common across these conditions) have also been shown to increase falls risk among individuals with these conditions. Given this broad range of risk factors, it can be hypothesised that multifactorial or multicomponent interventions targeting several risk factors would be more effective at reducing falls. The National Institute for Health and Care Excellence (NICE) guidelines for the assessment and treatment of falls currently recommend the use of multifactorial interventions among older adults [30]. This recommendation is supported by a recent systematic review that found multifactorial interventions reduced falls rate among older adults [73]. The nature of multifactorial interventions, differing components based on individual risk profiles, make them the most likely intervention approach to facilitate groups with mixed neurological conditions. Therefore, research to determine if this multifactorial approach is also effective for people with MS, PD and stroke should be a priority.

There was insufficient evidence identified to make definitive conclusions regarding the effectiveness of technology-based interventions, education-based interventions, environment/assistive technologies, cueing interventions, interventions to improve bone mineral density, and models of stroke care interventions at improving falls outcomes or to compare the interventions across conditions. These interventions were regularly only informed by one primary study. In addition, the quality of evidence for these interventions was primarily low to very low and so further research is warranted to determine their true effectiveness. Given the recent shift to remote treatment delivery, this should be a priority for researchers.

### Strengths and limitations

This umbrella review investigated the effectiveness of non-pharmacological falls prevention interventions for people with MS, PD and stroke and compared the similarities and differences across the three conditions. A robust methodology was used including the development of an a priori protocol, a comprehensive search strategy of databases, grey literature and reference lists, and the use of the AMSTAR 2 and GRADE to determine the strength of the evidence. There were also several limitations to this umbrella review. Firstly, the substantial heterogeneity and overall poor methodological quality of included systematic reviews precludes firm conclusions regarding the effectiveness of these interventions being drawn from the current evidence base. In addition, the language restrictions placed upon our searches may have resulted in relevant citations being missed. However, in an attempt to overcome this, authors of potentially relevant reviews that were published in another language were contacted to determine if an English version was available. Finally, the authors had planned on converting the estimates of effect presented in included reviews to one common measure of effect, however, given that the majority of reviews presented their results narratively, and the variation in interventions and outcomes assessed, this was not possible.

### Implications for practice

Exercise-based interventions are effective at reducing falls among people with PD both over the short- and long-term. The best available evidence identified in this umbrella review suggests that exercise-based interventions can reduce falls for people with stroke but no evidence of effect was identified for people with MS. However, much of the uncertainty regarding the effectiveness of these interventions can be attributed to the lack of high-quality studies informing outcomes and so a strong theoretical rationale remains for the use of exercise-based interventions to address modifiable physiological falls risk factors. There was insufficient evidence available for all other intervention types to determine their effectiveness, however, it can be hypothesised that the most effective method of reducing falls risk for individuals with MS, PD and stroke would be through an individualised multifactorial intervention with a core exercise programme and additional elements to address individual, specific needs.

### Implications for research

The authors of every systematic review included in this umbrella review recommended the development of further high-quality primary studies investigating falls prevention interventions for people with MS, PD and stroke, and this recommendation is re-emphasised by the authors of this umbrella review. A key limitation across included systematic reviews was the lack of reporting and heterogeneity of methods of falls data collection, fall definitions and falls outcomes. Particularly of note was the absence of data available across included systematic reviews with respect to injurious falls, with only one review reporting falls outcomes relating to physical injury [54]. Given that a key aim of falls prevention interventions is to reduce injurious falls, this data is of particular interest to service-planners and clinicians when deciding what programme to implement in the community. The large variation across included reviews prohibits cross-comparison of findings and pooling of data. Therefore, there is need for an international standard regarding research methods and outcomes for studies investigating falls among people with PD, MS and stroke.

## Conclusions

Given the negative consequences associated with falls for people with MS, PD and stroke, the development and implementation of theory-based and effective falls prevention interventions is a research priority. Exercise-based interventions have been found to be effective at reducing falls among people with PD, however, the evidence for exercise-based interventions for people with MS and stroke is less conclusive. In addition, conclusions regarding all other intervention types could not be drawn due to insufficient evidence. To progress research in this field, the focus should be on the development of high-quality trials investigating the effectiveness of falls prevention interventions for people with MS, PD and stroke, rather than the publication of further systematic reviews.

### Supplementary Information

**Additional file 1.** Reasons for exclusions following full-text screen.

**Additional file 2.** Matrix of evidence table demonstrating degree of overlap and citation count of systematic reviews meeting inclusion criteria.

**Additional file 3.** Characteristics of included reviews.

**Additional file 4.** Application of GRADE algorithm to falls outcomes.

## Data Availability

All data generated or analysed during this study are included in this published article and its supplementary information files.

## Appendix

### Search strategy for CINAHL

**S1:** TI (falls OR fall* OR “accidental fall”) OR AB (falls OR fall* OR “accidental fall”).

**S2:** TI (stroke OR CVA OR cerebrovascular OR apoplexy OR vascular OR MS OR “multiple sclerosis” OR demyelin* OR PD OR “parkinson’s disease” OR “parkinson disease” OR parkinson* OR neurol*) OR AB (stroke OR CVA OR cerebrovascular OR apoplexy OR vascular OR MS OR “multiple sclerosis” OR demyelin* OR PD OR “parkinson’s disease” OR “parkinson disease” OR parkinson* OR neurol*).

**S3:** (TI stroke OR CVA OR cerebrovascular OR apoplexy OR vascular OR MS OR “multiple sclerosis” OR demyelin* OR PD OR “parkinson’s disease” OR “parkinson disease” OR parkinson* OR neurol* OR AB stroke OR CVA OR cerebrovascular OR apoplexy OR vascular OR MS OR “multiple sclerosis” OR demyelin* OR PD OR “parkinson’s disease” OR “parkinson disease” OR parkinson* OR neurol*) AND (S1 AND S2).

**S4:** TI (intervention OR prevention OR rehabilitation OR treatment OR therap*) OR AB (intervention OR prevention OR rehabilitation OR treatment OR therap*).

**S5:** (TI intervention OR prevention OR rehabilitation OR treatment OR therap* OR AB intervention OR prevention OR rehabilitation OR treatment OR therap*) AND (S3 AND S4).

**S6:** TI (systematic OR review OR “meta-analysis”) OR AB (systematic OR review OR “meta-analysis”).

**S7:** (TI systematic OR review OR “meta-analysis” OR AB systematic OR review OR “meta-analysis”) AND (S5 AND S6)

## Abbreviations

PD: Parkinson’s Disease
MS: Multiple Sclerosis
RCT: Randomised Controlled Trial
PRIOR: Preferred Reporting Items for Overviews of Reviews
JBI: Joanna Briggs Institute
PRISMA: Preferred Reporting Items for Systematic Reviews and Meta-Analysis
AMSTAR 2: A MeaSurement Tool to Assess Systematic Reviews 2
GRADE: Grading of Recommendations Assessments, Development and Evaluation
FES: Functional Electrical Stimulation
NICE: National Institute for Health and Care Excellence
