# Supplementary material for: Effectiveness of interventions to prevent falls for people with multiple sclerosis, Parkinson’s disease and stroke: an umbrella review
Source: BMC Neurol. 2021 Sep 29;21:378. doi: 10.1186/s12883-021-02402-6 (PMC8480085; doi:10.1186/s12883-021-02402-6)
Supplement: Supplementary file 1 — Additional file 1. Reasons for exclusions following full-text screen. [file 12883_2021_2402_MOESM1_ESM.docx]

**Effectiveness of interventions to prevent falls for people with Multiple Sclerosis, Parkinson’s Disease and stroke: An umbrella review**

**Authors**

Nicola O’Malley^1,2^, Amanda M. Clifford^1,2^, Mairéad Conneely^1,2^, Bláthín Casey^3,4^ and Susan Coote^1,4,5^

***Author’s Affiliations:***

^1^School of Allied Health, Faculty of Education and Health Sciences, University of Limerick, Ireland.

^2^Ageing Research Centre, Health Research Institute, University of Limerick, Ireland.

^3^Department of Physical Education and Sport Sciences, Faculty of Education and Health Sciences, University of Limerick.

^4^Centre of Physical Activity for Health, Health Research Institute, University of Limerick, Limerick, Ireland.

^5^Multiple Sclerosis Society of Ireland.

**Corresponding Author**

Name: Nicola O'Malley

Postal address: School of Allied Health, Faculty of Education and Health Sciences, University of Limerick, Ireland.

Phone number: +353 61 234118

Email: [Nicola.OMalley@ul.ie](mailto:Nicola.OMalley@ul.ie)

**Supplementary File 1:** Characteristics of excluded reviews.

| **Reason for exclusion** | **Reference** |
| --- | --- |
| Ineligible population | **Barreto** PDS, Rolland Y, Vellas B, Maltais M. Association of Long-term Exercise Training With Risk of Falls, Fractures, Hospitalizations, and Mortality in Older Adults: A Systematic Review and Meta-analysis. JAMA Internal Medicine. 2019;179 3:394-405. |
| Ineligible population | **Booth** V, Hood V, Kearney F. Interventions incorporating physical and cognitive elements to reduce falls risk in cognitively impaired older adults: a systematic review. JBI Evidence Synthesis. 2016;14 5:110-135. |
| Ineligible population | **Clemson** L, Mackenzie L, Ballinger C, Close JC, Cumming RG. Environmental interventions to prevent falls in community-dwelling older people: a meta-analysis of randomized trials. Journal of Aging and Health. 2008;20 8:954-971. |
| Ineligible population | **Costello** E, Edelstein JE. Update on falls prevention for community-dwelling older adults: review of single and multifactorial intervention programs. JRRD. 2008;45 8:1135-1152. |
| Ineligible population | **Gard G.** Prevention of slip and fall accidents: risk factors, methods and suggestions for prevention. Methods and Suggestions for Prevention, Physical Therapy Reviews. 2000;5 3:175-182. |
| Ineligible population | **Hopewell** S, Copsey B, Nicolson P, Adedire B, Boniface G, Lamb S. Multifactorial interventions for preventing falls in older people living in the community: a systematic review and meta-analysis of 41 trials and almost 20000 participants. Br J Sports Med 2020;54 22:1340–1350. |
| Ineligible population | **Hughes** KJ, Salmon N, Galvin R, Basey B, Clifford AM. Interventions to improve adherence to exercise therapy for falls prevention in community-dwelling older adults: systematic review and meta-analysis. Age and Ageing. 2019;48:185-195. |
| Ineligible population | **Lewis** M, Peiris CL, Shields N. Long-term home and community-based exercise programs improve function in community-dwelling older people with cognitive impairment: a systematic review. Journal of Physiotherapy. 2017;63:23-29. |
| Ineligible population | **Liu** CJ, Latham NK. Progressive resistance strength training for improving physical function in older adults. Cochrane Database of Systematic Reviews. 2009; DOI: 10.1002/14651858.CD002759.pub2. |
| Ineligible population | **Manojlovich** M, Lee S, Lauseng D. A Systematic Review of the Unintended Consequences of Clinical Interventions to Reduce Adverse Outcomes. J Patient Saf. 2016;12 4:173-179. |
| Ineligible population | **Medical Advisory Secretariat**. Prevention of falls and fall-related injuries in community-dwelling seniors: an evidence-based analysis. Ontario Health Technology Assessment Series. 2008;8 2. |
| Ineligible population | **Meereis-Lemos** ECW, Guadagnin EC, Mota CB. Influence of strength training and multicomponent training on the functionality of older adults: systematic review and meta-analysis. Rev Bras Cineantropom Desempenho Hum. 2020;22:e60707. |
| Ineligible population | **Rezapour** A, Hosseinijebeli SS, Faradonbeh SB. Economic evaluation of E‑health interventions compared with alternative treatments in older persons’ care: A systematic review. J Edu Health Promot. 2020; DOI: 10.4103/jehp.jehp_787_20. |
| Ineligible population | **Teng** B, Gomersall SR, Hatton A, Brauer SG. Combined group and home exercise programmes in community-dwelling falls-risk older adults: Systematic review and meta-analysis. Physiother Res Int. 2020;25 3:e1839. |
| Ineligible population | **Totten** AM, White-Chu EF, Wasson N, Morgan E, Kansagara D, Davis-O’Reilly C, et al. Home-Based Primary Care Interventions. (Prepared by the Pacific Northwest Evidence-based Practice Center under Contract No. 290-2012-00014-I.). 2016; AHRQ Publication No. 15(16)-EHC036-EF. Rockville, MD: Agency for Healthcare Research and Quality. |
| Ineligible population | **Voigt-Radloff** S, Ruf G, Vogel A, van Nes F, Hüll M. Occupational therapy for elderly: Evidence mapping of randomised controlled trials from 2004–2012. Zeitschrift für Gerontologie und Geriatrie. 2015;48 1:52-72. |
| Ineligible population | **Ye** P, Liu Y, Zhang J, Peng K, Pan X, Shen Y, et al. Falls prevention interventions for community-dwelling older people living in mainland China: a narrative systematic review. BMC Health Services Research. 2020;20:808. |
| Unable to extract population specific data | **Avanecean** D, Calliste D, Contreras T, Lim Y, Fitzpatrick A. Effectiveness of patient-centered interventions on falls in the acute care setting compared to usual care: a systematic review. JBI Database of Systematic Reviews and Implementation Reports. 2017;15 12:3006-3048. |
| Unable to extract population specific data | **Demaille-Wlodyka** S, Donze C, Givron P, Gallien P, ETP Sofmer Group. Self care programs and multiple sclerosis: Physical therapeutics treatment - literature review. Annals of Physical and Rehabilitation Medicine. 2011;54:109-128. |
| Unable to extract population specific data | **Montero-Odasso** M, Speechley M. Falls in Cognitively Impaired Older Adults: Implications for Risk Assessment And Prevention. Journal of the American Geriatrics Society. 2018;66 2:367-375. |
| Unable to extract population specific data | **Tricco** AC, Thomas SM, Veronki AA, Hamid JS, Cogo E, Strifler L, et al. Quality improvement strategies to prevent falls in older adults: a systematic review and network meta-analysis. Age and Ageing. 2019;48:337-346. |
| Falls outcome not measured | **Betts** L. A review of the use of lower limb orthoses and devices in stroke. British Journal of Neuroscience. 2016;12:S15-S18. |
| Falls outcome not measured | **Bhidayasiri** R, Jitkritsadakul O, Boonrod N, Sringean J, Calne SM, Hattori N, et al. What is the evidence to support home environmental adaptation in Parkinson's disease? A call for multidisciplinary interventions. Parkinsonism Relat Disord. 2015;21 10:1127-32. |
| Falls outcome not measured | **Bishop** L, Bushnell C. Post Hospital Discharge Care for Complex Chronic Conditions: the Unique Challenges Facing Stroke Patients in their Homes. Current Cardiovascular Risk Reports. 2017;11 12: DOI: 10.1007/s12170-017-0560-7. |
| Falls outcome not measured | **Braz** NFT, Dutra LR, Medeiros PES, Scianni AA, Faria CDCDM. Eficácia do Nintendo Wii em desfechos funcionais e de saúde de indivíduos com doença de Parkinson: uma revisão sistemática. Fisioterapia e Pesquisa. 2018;25 1:100-106. |
| Falls outcome not measured | **Buechter** RB, Fechtelpeter D. Climbing for preventing and treating health problems: a systematic review of randomized controlled trials. GMS German Medical Science. 2011;9 DOI: 10.3205/000142. |
| Falls outcome not measured | **Byrd** EM, Jablonski RJ, Vance, DE. Understanding Anosognosia for Hemiplegia After Stroke. Rehabilitation Nursing. 2020;45 1:3-15. |
| Falls outcome not measured | **Byrnes** K, Wu PJ, Whillier S. Is Pilates an effective rehabilitation tool? A systematic review. Journal of Bodywork and Movement Therapies. 2017;22 1:192-202. |
| Falls outcome not measured | **Casuso-Holgado** MJ, Martin-Valero R, Carazo AF, Medrano-Sánchez EM, Cortés-Vega MD, et al. Clinical Rehabilitation. 2018;32 9:1220-1234. |
| Falls outcome not measured | **Chavoliat** S, Taylor NF, Dodd KJ. Sit-to-stand exercise programs improve sit-to-stand performance in people with physical impairments due to health conditions: a systematic review and meta-analysis. Disability and Rehabilitation. 2019; DOI: 10.1080/09638288.2018.1524518. |
| Falls outcome not measured | **Chen** BL, Guo JB, Liu MS, Li X, Zou J, Chen X, et al. Effect of Traditional Chinese Exercise on Gait and Balance for Stroke: A Systematic Review and Meta-Analysis. PLoS ONE. 2015;10 8:e0135932. |
| Falls outcome not measured | **Chung** LH, Thilarajah S, Tan D. Effectiveness of resistance training on muscle strength and physical function in people with Parkinson's disease: A systematic review and meta-analysis. Clinical Rehabilitation. 2016;30 1:11-23. |
| Falls outcome not measured | **Costa** PS, Bôas ECCV, Fonseca EP. Effectiveness of gait training in water for patients with parkinson’s disease: systematic review. Phys Res. 2018;8 4:551-557. |
| Falls outcome not measured | **Daviet** JC, Bonan I, Claire JM, Colle F, Damamme L, Froger J, et al. Therapeutic patient education for stroke survivors: Non-pharmacological management. A literature review. Annals of Physical and Rehabilitation Medicine. 2012;55 9:641-656. |
| Falls outcome not measured | **De Freitas** TB, Leite PHW, Doná F, Pompeu JE, Swarowsky A, Torriani-Pasin C. The effects of dual task gait and balance training in Parkinson's disease: a systematic review. Physiotherapy Theory and Practice. 2018; DOI: 10.1080/09593985.2018.1551455. |
| Falls outcome not measured | **Dennett** AM, Taylor NF. Machines that go "ping" may improve balance but may not improve mobility or reduce risk of falls: a systematic review. J Rehabil Med. 2015;47 1:18-30. |
| Falls outcome not measured | **Dockx** K, Bekkers EMJ, Van den Bergh V, Ginis P, Rochester L, Hausdorff JM, et al. Virtual reality for rehabilitation in Parkinson’s disease. Cochrane Database of Systematic Reviews. 2016; DOI: 10.1002/14651858.CD010760. |
| Falls outcome not measured | **English** C, Hillier SL, Lynch EA. Circuit class therapy for improving mobility after stroke. Cochrane Database of Systematic Reviews. 2017; DOI: 10.1002/14651858.CD007513.pub3. |
| Falls outcome not measured | **Ferraz** DD, Trippo K, Dominiguez A, Santos A Filho JO. Nintendo Wii training on postural balance and mobility rehabilitation of adults with Parkinson's disease: a systematic review. Fisioter Mov. 2013;30:383-393. |
| Falls outcome not measured | **French** B, Thomas LH, Coupe J, McMahon NE, Connell L, Harrison J, et al. Repetitive task training for improving functional ability after stroke. Cochrane Database of Systematic Reviews. 2016; DOI: 10.1002/14651858.CD006073.pub3. |
| Falls outcome not measured | **French** B, Thomas L, Leathley M, Sutton C, McAdam J, Forster A, et al. 2010;42:9-15. |
| Falls outcome not measured | **Ghai** S, Ghai I, Effenberg AO. Effects of dual tasks and dual-task training on postural stability: a systematic review and meta-analysis. Clin Interv Aging. 2017;12:557-577. |
| Falls outcome not measured | **Gómez-González** J, Martín-Casas P, Cano-de-la-Cuerda R. Effects of auditory cues on gait initiation and turning in patients with Parkinson’s disease. Neurología. 2019;34 6:396-407. |
| Falls outcome not measured | **Green** E, Huynh A, Broussard L, Zunker B, Matthews J,Hilton CL, et al. Systematic Review of Yoga and Balance: Effect on Adults With Neuromuscular Impairment. American Journal of Occupational Therapy. 2019;73 1:1-11. |
| Falls outcome not measured | **Grimbergen** YAM, Munneke M, Bloem BR. Falls in Parkinson’s Disease. Curr Opin Neurol. 2004;17 4:405-15. |
| Falls outcome not measured | **Guerra Padilla** M, Molina Rueda F, Alguacil Diego IM. Effecto de la ortesis de tobillo pie en el control postural tras el accidente cerebrovascular: revision sistemática. Neurología. 2014;29:423-432. |
| Falls outcome not measured | **Harris** MH, Holden MK, Cahalin MK, Fitzpatrick D, Lowe S, Canavan PK. Gait in older adults: A review of the literature with an emphasis toward achieving favorable clinical outcomes, Part II. Clinical Geriatrics. 2008;16 8:37-45. |
| Falls outcome not measured | **Hollands** KL, Pelton TA, Tyson SF, Hollands MA, van Vliet PM. Interventions for coordination of walking following stroke: systematic review. Gait & Posture. 2012;35 3:349-359. |
| Falls outcome not measured | **Hollands** K, van Vliet P, Pelton T. Interventions for improving coordination of axial segments and lower limbs during walking following stroke: Systematic Review. JBI Library of Systematic Reviews. 2011;10 22:1260-1362. |
| Falls outcome not measured | **Jahnke** R, Larkey L, Rogers C, Etnier J, Lin F. A Comprehensive Review of Health Benefits of Qigong and Tai Chi. Am J Health Promot. 2010;24 6:e1-e25. |
| Falls outcome not measured | **Kang** N, Lee RD, Lee JH, Hwang MH. Functional Balance and Postural Control Improvements in Patients With Stroke After Noninvasive Brain Stimulation: A Meta-analysis. Archives of Physical Medicine & Rehabilitation. 2020; 101 1:141-153. |
| Falls outcome not measured | **Li** Z, Han XG, Sheng J, Ma SJ. Virtual reality for improving balance in patients after stroke: A systematic review and meta-analysis. Clinical Rehabilitation. 2016;30 5:432-420. |
| Falls outcome not measured | **Lim** I, van Wegen E, de Goede C, Deutekon M, Nieuwboer A, Willems A, et al. Effects of external rhythmical cueing on gait in patients with Parkinson's disease: a systematic review. Clinical Rehabilitation. 2005;19 7:695-713. |
| Falls outcome not measured | **Lima** LO, Scianni A, Rodrigues-de-Paula F. Progressive resistance exercise improves strength and physical performance in people with mild to moderate Parkinson’s disease: a systematic review. Journal of Physiotherapy. 2013;59:7-13. |
| Falls outcome not measured | **Maetzler** W, Nieuwhof F, Hasmann SE, Bloem BR. Emerging therapies for gait disability and balance impairment: promises and pitfalls. Mov Disord. 2013;28 11:1576-86. |
| Falls outcome not measured | **Mandelbaum** R, Lo AC. Examining Dance as an Intervention in Parkinson's Disease: A Systematic Review. AM J Dance Ther. 2014;36 2:160-175. |
| Falls outcome not measured | **Mehrholz** J, Thomas S, Elsner B. Treadmill training and body weight support for walking after stroke. Cochrane Database of Systematic Reviews. 2017; DOI: 10.1002/14651858.CD002840. |
| Falls outcome not measured | **Mehrholz** J, Kugler J, Storch A, Pohl M, Hirsch K, Elsner B. Treadmill training for patients with Parkinson’s disease. Cochrane Database of Systematic Reviews. 2015; DOI: 10.1002/14651858.CD007830.pub4. |
| Falls outcome not measured | **Mendes** LA, Lima INDF, Souza T, do Nascimento GC, Resqueti VR, Fregonezi GAF. Motor neuroprosthesis for promoting recovery of function after stroke. Cochrane Database of Systematic Reviews. 2020; DOI: 10.1002/14651858.CD012991.pub2. |
| Falls outcome not measured | **Mille** ML, Creath RA, Prettyman MG, Hilliard MJ, Martinez KM, MacKinnon CD, et al. Posture and Locomotion Coupling: A Target for Rehabilitation Interventions in Persons with Parkinson's Disease. Parkinson’s Disease. 2012; DOI: 10.1155/2012/754186. |
| Falls outcome not measured | **Mohamed Suhaimy** MSB, Okubo Y, Hoang PD, Lord SR. Reactive Balance Adaptability and Retention in People With Multiple Sclerosis: A Systematic Review and Meta-Analysis. 2020;34 8:675-685. |
| Falls outcome not measured | **Morris** ME, Ellis TD, Jazayeri D, Heng H, Thomson A, Balasundaram AP, et al. Boxing for Parkinson’s Disease: Has Implementation Accelerated Beyond Current Evidence? Front Neurol. 2019;10 DOI: 10.3389/fneur.2019.01222. |
| Falls outcome not measured | **Morris** ME, Martin Cl, Schenkman ML. Striding Out With Parkinson Disease: Evidence-Based Physical Therapy for Gait Disorders. Physical Therapy. 2020;90 2:280-288. |
| Falls outcome not measured | **Muroi** D, Ohtera S, Kataoka Y, Banno M, Tsujimoto Y, Tsujimoto H, et al. Obstacle avoidance training for individuals with stroke: a systematic review and meta-analysis. BMJ Open. 2019;9 12:e028873. |
| Falls outcome not measured | **Paton** J, Hatton AL, Rome K, Kent B. Effects of foot and ankle devices on balance, gait and falls in adults with sensory perception loss: a systematic review. JBI Database of Systematic Reviews & Implementation Reports. 2016;14 12:127-162. |
| Falls outcome not measured | **Snook** EM, Motl RW. Effect of Exercise Training on Walking Mobility in Multiple Sclerosis: A Meta-Analysis. Neurorehabilitation and Neural Repair. 2009;23 2:108-116. |
| Falls outcome not measured | **Tally** Z, Boetefuer L, Kauk C, Perez G, Schrand L, Hoder J. The efficacy of treadmill training on balance dysfunction in individuals with chronic stroke: a systematic review. Topics in Stroke Rehabilitation. 2017;24 7:539-546. |
| Falls outcome not measured | **Taylor** E, Taylor-Piliae RE. The effects of Tai Chi on physical and psychosocial function among persons with multiple sclerosis: A systematic review. Complementary Therapies in Medicine. 2017;31:100-108. |
| Falls outcome not measured | **Wang** C, Collet JP, Lau J. The Effect of Tai Chi on Health Outcomes in Patients With Chronic Conditions. Arch Intern Med. 2004;164 5:493-501. |
| Falls outcome not measured | **Webster** D, Celik O. Systematic review of Kinect applications in elderly care and stroke rehabilitation. Journal of Neuroengineering and Rehabilitation. 2014;11:108. |
| Falls outcome not measured | **Wilkins** S, Jung B, Wishart L, Edwards M, Norton SG. The effectiveness of community-based occupational therapy education and functional training programs for older adults: a critical literature review. Canadian Journal of Occupational Therapy. 2003;70 4:214-225. |
| Falls outcome not measured | **Winser** SJ, Paul LF, Magnus LKL, Yan S, Shenug TP, Sing YM, et al. Economic Evaluation of Exercise-Based Fall Prevention Programs for People with Parkinson's Disease: A Systematic Review. Journal of Alternative & Complementary Medicine. 2019;25 12:1225-1237. |
| Falls outcome not measured | **Yang** Y, Li XY, Gong L, Zhu YL, Hao YL. Tai Chi for Improvement of Motor Function, Balance and Gait in Parkinson’s Disease: A Systematic Review and Meta-Analysis. PLoS ONE. 2014;9 7:e102942. |
| Falls outcome not measured | **Zanotto** T, Bergamin M, Roman F, Sieverdes JC, Gobbo S, Zaccaria M, et al. Effect of exercise on dual-task and balance on elderly in multiple disease conditions. Current aging science. 2014;7 2:115-136. |
| Unable to extract falls data | **Lauzé** M, Daneault JF, Duval C. The Effects of Physical Activity in Parkinson’s Disease: A Review. Journal of Parkinson’s Disease. 2016;6:685-698. |
| Ineligible intervention | **Muzerengi** S, Herd C, Rick C, Clarke CE. A systematic review of interventions to reduce hospitalisation in Parkinson’s disease. Parkinsonism and Related Disorders. 2016;24:3-7. |
| No primary studies investigating effect of intervention | **Keus** SH, Munneke M, Nijkrake MJ, Kwakkel G, Bloem BR. Physical therapy in Parkinson's disease: Evolution and future challenges. Movement Disorders. 2009;24 1:1-14. |
| No primary studies investigating effect of intervention | **Opara** J, Blaszczyk J, Dyskiewicz A. Prevention of falls in Parkinson Disease. Rehabilitacja Medyczna. 2005;9 1:25-28. |
| No primary studies investigating effect of intervention | **Roth** EJ, Diaz S. Falls during stroke rehabilitation: A review of the literature. Topics in Stroke Rehabilitation. 1995;2 1:82-90. |
| Not available in English language | **Bakker** M, Munneke M, Keus SH, Bloem BR. Postural instability and falls in patients with Parkinson's disease. 2004;114 3:63-6. |
| Not available in English language | **Dos Santos** VV, Leite MAA, Silveira R, Antoniolli R, Nascimento OJM, de Freitas MRG. Fisioterapia na doença de Parkinson: Uma breve revisão. Revista Brasileira de Neurologia. 2010;46 2:17-25. |
| Not available in English language | **Guijarro** EV, García MTF. Effect of dancing on Parkinson's disease patients. Fisioterapia. 2012;34 5:216-224. |
| Not available in English language | **Klemenov** AV. Backward walking and its application in geriatric patients. Advances in Gerontology. 2018;31 3:428-432. |
| Not available in English language | **Laguna-Parras** JM, Carrscosa-Corral RR, Zafra López F, Carrcosa-García MI, Martínez FML, Esteban JAA, et al. Efectividad de las intervenciones para la prevención de caídas en ancianos: revisión sistemática. Gerokonos. 2010;21 3:97-107. |
| Not available in English language | **Li** ZY, Lu FP, Chan DC. Risk factors, evaluation and prevention of falls in older adults. Journal of Internal Medicine of Taiwan. 2014;25 3:137-142. |
| Not available in English language | **Miletic** M, Kokic IS, Vuletic V. The effect of exercise programs on fall prevention for people with Parkinson's disease. Hrvatska Revija Za Rehabilitacijska Istrazivanja. 2013; 49 2:172-179. |
| Not available in English language | **Múniz-Hellín** E, Cano-de-la-Cuerda R, Miangolarra-Page JC. Visual cues as a therapeutic tool in Parkinson's disease. A systematic review. 2013;48 4:190-197. |
| Not available in English language | **Yilidrim** P. The effects and clinical implementations of Tai Chi exercise. Turkiye Fiziksel Tip ve Rehabilitasyon Dergisi. 2014;60:S36-S42. |
| Protocol | **Abou** L, Rice L, Du Y, Alluri A. Effectiveness of interventions in reducing fear of falling in individuals with neurological disorders: a systematic review. PROSPERO. 2019; CRD42019135628. Available from: https://www.crd.york.ac.uk/prospero/display_record.php?RecordID=135628. |
| Protocol | **Almeida** S, Braga-Neto P, Sobreira E, Bonfadini J, Sobreira-Neto M, Nascimento S, et al. Effect of power training in Parkinson's disease patients on physical functional performance: systematic review and meta-analysis of randomized controlled trials or quasi-randomized trials. PROSPERO. 2019; CRD42019139446. Available from: https://www.crd.york.ac.uk/prospero/display_record.php?ID=CRD42019139446. |
| Protocol | **Büchter** RB, Kienle L, Pieper D, Prediger B. Update of a systematic review of randomised controlled trials on the clinical evidence of therapeutic climbing interventions. PROSPERO. 2019; CRD42019128691. Available from: https://www.crd.york.ac.uk/prospero/display_record.php?ID=CRD42019128691. |
| Protocol | **Canning** CG, Allen NE, Bloem BR, Keus SH, Munneke M, Nieuwboer A, et al. Interventions for prevention of falls in people with Parkinson's disease: A protocol for a systematic review. Movement Disorders. 2014;29: S229. |
| Protocol | **Caseroti** P, Piras F, Piras F, Olsen PØ, Albertsen LL, Tanous D. Is combined physical and cognitive training more effective than either alone for improving gait, falls, and self-reported disability in older adults? A systematic review. PROSPERO. 2019; CRD42019131657. Available from: https://www.crd.york.ac.uk/prospero/display_record.php?ID=CRD42019131657. |
| Protocol | **Chen** PC, Chuang CH, Leong CP, Bai CH, Tu YK, Chiang LC. Comparing different types of exercises for fall prevention in older people living in the community: a systematic review and network meta-analysis of randomized controlled trials. PROSPERO. 2015; CRD42015029809. Available from: https://www.crd.york.ac.uk/prospero/display_record.php?ID=CRD42015029809. |
| Protocol | **Cheng** FY, Liu HH, Yeh NC, Wu YF, Lee HC, Yang YR, et al. Effect of tai chi to reduce falls and improve balance performance in Parkinson's disease: a systematic review and meta-analysis. PROSPERO. 2018; CRD42018073565. Available from: https://www.crd.york.ac.uk/prospero/display_record.php?ID=CRD42018073565. |
| Protocol | **Cooper** K, Alexander L, Swinton P, Pavlova A, Kirkpatrick P, Stephen A. The effectiveness of falls prevention technology on falls outcomes in hospitalized patients. PROSPERO. 2019; CRD42019128789. Available from: https://www.crd.york.ac.uk/prospero/display_record.php?ID=CRD42019128789. |
| Protocol | **Dennett** A, Taylor N. Do machines that go 'ping' (electronic devices designed to improve balance using feedback) improve balance, mobility and reduce falls? PROSPERO. 2013; CRD42013005726. Available from: https://www.crd.york.ac.uk/prospero/display_record.php?ID=CRD42013005726. |
| Protocol | **Domingues-Montari** S, Stuckey. Telehealth interventions in the home to reduce the incidence of falls among older adults: a systematic review. PROSPERO. 2018; CRD42018084737. Available from: https://www.crd.york.ac.uk/prospero/display_record.php?ID=CRD42018084737. |
| Protocol | **Giacomino** K, Sattelmayer M, Hilfiker R. Living systematic review with a network meta-analysis on interventions for patients with unilateral neglect after stroke with continuously updated results on a free website. PROSPERO. 2018; CRD42018110993. Available from: https://www.crd.york.ac.uk/prospero/display_record.php?ID=CRD42018110993. |
| Protocol | **Goh** L, Canning C, Allen N Song J, Clemson L. Allied health interventions for freezing of gait in Parkinson’s disease: a systematic review and meta-analysis. PROSPERO. 2018; CRD42018116820. Available from: https://www.crd.york.ac.uk/prospero/display_record.php?ID=CRD42018116820. |
| Protocol | **Gomersall** J, Tufanaru C, White S. The cost effectiveness of exercise for preventing falls in older people living in the community: A systematic review. JBI Evidence Synthesis. 2012;10 57:3949-3959. |
| Protocol | **Green** E, Broussard L, Huynh A, Zunker B, Mathews J, Aranha K, et al. Systematic review of yoga and balance: effect on risk of falls. PROSPERO. 2016; CRD42016051134. Available from: https://www.crd.york.ac.uk/prospero/display_record.php?ID=CRD42016051134. |
| Protocol | **Hewitt** J, Refshauge K, Nightingale J, Hiller C, Henwood T, Tam E, et al. Exercise for falls prevention in residential aged care and its effects on quality of life and mobility: a systematic review and meta-analysis. PROSPERO. 2017; CRD42017069846. Available from: https://www.crd.york.ac.uk/prospero/display_record.php?ID=CRD42017069846. |
| Protocol | **Hofheinz** M, Mibs M, Elsner B. Dual task training for improving balance and gait in people with stroke [Cochrane Protocol]. PROSPERO. 2017; CRD42017055003. Available from: https://www.crd.york.ac.uk/prospero/display_record.php?ID=CRD42017055003. |
| Protocol | **Hua** W, Minghui Q, Xudong G, Meifang S. Robot-assisted gait therapy (Lokomat) for stroke: systematic review and meta analysis. PROSPERO. 2016; CRD42016046051. Available from: https://www.crd.york.ac.uk/prospero/display_record.php?ID=CRD42016046051. |
| Protocol | **Lee** MS, Kim TH, Shin BC, Ernst E. Mind-body movement therapies for Parkinson's disease [Cochrane Protocol]. PROSPERO. 2015; CRD42015019597. Available from: https://www.crd.york.ac.uk/prospero/display_record.php?ID=CRD42015019597. |
| Protocol | **Lee** M, Lee NJ, Seo HJ, Jang H. Interventions involving patients and families to improve patient safety: a systematic review. PROSPERO. 2018; CRD42018096162. Available from: https://www.crd.york.ac.uk/prospero/display_record.php?ID=CRD42018096162. |
| Protocol | **Li** FP, Leung KLM, Yuen MS, Szeto D, Tsui PS, Winser SJ. Cost-effectiveness of falls prevention programs for people with Parkinson’s disease: a systematic review. PROSPERO. 2018; CRD42018105296. Available from: https://www.crd.york.ac.uk/prospero/display_record.php?ID=CRD42018105296. |
| Protocol | **Li** J, Jin R, He M, Zhong D, Zheng H. Tai Chi for post-stroke patients with balance dysfunction: a protocol of systematic review. PROSPERO. 2018; CRD42018092218. Available from: https://www.crd.york.ac.uk/prospero/display_record.php?ID=CRD42018092218. |
| Protocol | **Li** J, Zhong D, Ye J, He M, Liu X, Zheng H et al. Rehabilitation for balance impairment in patients after stroke: a protocol of a systematic review and network meta-analysis. BMJ Open. 2019;9 7:e026844. |
| Protocol | **Lu** Y, Abou L, Rice L. Effects of physical therapy interventions for reducing falls in people with multiple sclerosis: a systematic review. PROSPERO. 2020; CRD42020150297. Available from: https://www.crd.york.ac.uk/prospero/display_record.php?ID=CRD42020150297. |
| Protocol | **Min** BY, Okubu Y, Brodie M, Lord S, Canning C, Hoang P. Effects of reactive and volitional step training on falls risk and incidence among people with neurological diseases: a systematic review and possible meta-analyses. PROSPERO. 2019; CRD42019127095. Available from: https://www.crd.york.ac.uk/prospero/display_record.php?ID=CRD42019127095. |
| Protocol | **Morales** NM, Holgado MJC, Bujalance L. Physical exercise required for the reduction of fall incidence in ageing: a systematic review. PROSPERO. 2018; CRD42018082356. Available from: https://www.crd.york.ac.uk/prospero/display_record.php?ID=CRD42018082356. |
| Protocol | **Muroi** D, Ohtera S, Banno M, Tsujimoto H, Tsujimoto Y, Kataoka Y, et al. A systematic review of obstacle avoidance exercises in patients with stroke. PROSPERO. 2017; CRD42017060691. Available from: https://www.crd.york.ac.uk/prospero/display_record.php?ID=CRD42017060691. |
| Protocol | **Ni** X, Liu S, Lu F, Guo X, Shi X. Tai chi for Parkinson's disease: a systematic review and meta-analysis. PROSPERO. 2013; CRD42013004989. Available from: https://www.crd.york.ac.uk/prospero/display_record.php?ID=CRD42013004989. |
| Protocol | **Nicks** R, Robertson B, Waddell C, Lannin N. A systematic review of the effectiveness of occupational therapy prescribed home modifications. PROSPERO. 2017; CRD42017015394. Available from: https://www.crd.york.ac.uk/prospero/display_record.php?ID=CRD42017015394. |
| Protocol | **Northcote** M. The effect of Tai Chi and Qigong to improve falls, balance and gait in Parkinson’s disease patients: a systematic review and meta-analysis. PROSPERO. 2019; CRD42019121224. Available from: https://www.crd.york.ac.uk/prospero/display_record.php?ID=CRD42019121224. |
| Protocol | **Nsobundu** C, Alonge O, Jani S, Foster M, Smith M, Ory M. The effect of yoga on falls and/or fear of falling related outcomes in adults 50 years and older. PROSPERO. 2019; CRD42019135747. Available from: https://www.crd.york.ac.uk/prospero/display_record.php?ID=CRD42019135747. |
| Protocol | **Owen** C, Roberts H, Ibrahim K. What is the effectiveness of falls-based self-management interventions for people with Parkinson's disease in improving psychological and health-related outcomes? PROSPERO. 2017; CRD42017052585. Available from: https://www.crd.york.ac.uk/prospero/display_record.php?ID=CRD42017052585. |
| Protocol | **Park** M, Giap TTT. Impact of patient and family involvement interventions on patient safety: a meta-analysis. PROSPERO. 2019; CRD42019131183. Available from: https://www.crd.york.ac.uk/prospero/display_record.php?ID=CRD42019131183. |
| Protocol | **Paton** J, Collings R, Glasser S, Kent B. The effects of foot and ankle devices on balance, gait and falls in adults with sensory perception loss: a systematic review protocol. JBI Evidence Synthesis. 2014;12 11:74-91. |
| Protocol | **Robinson** A, Snowdon D, Dennett A. Does task specific training improve mobility in progressive neurological conditions? PROSPERO. 2016; CRD42016047334. Available from: https://www.crd.york.ac.uk/prospero/display_record.php?ID=CRD42016047334. |
| Protocol | **Saracoglu** I, Arik MI, Kiloatar H. The effectiveness of Pilates exercises on balance in patients with multiple sclerosis: a systematic review and meta-analysis. PROSPERO. 2019; CRD42019145924. Available from: https://www.crd.york.ac.uk/prospero/display_record.php?ID=CRD42019145924. |
| Protocol | **Shrivastava** S, Prabhu R, Kirubakaran R, Thomasraj J, Sundaram B. Ankle foot orthosis for walking in stroke rehabilitation [Cochrane Protocol]. PROSPERO. 2015; CRD42015019602. Available from: https://www.crd.york.ac.uk/prospero/display_record.php?ID=CRD42015019602. |
| Protocol | **Silva** FC. Effects of hydrotherapy in balancing patients with Parkinson's disease: a systematic review of randomized clinical trials. PROSPERO. 2014; CRD42014007229. Available from: https://www.crd.york.ac.uk/prospero/display_record.php?ID=CRD42014007229. |
| Protocol | **Winser** S, Kannan P, Krishnamurthy K, Tsang W. Tai Chi for improving balance and gait in neurological disorders: systematic review and meta-analysis. PROSPERO. 2016; CRD42016043086. Available from: https://www.crd.york.ac.uk/prospero/display_record.php?ID=CRD42016043086. |
| Protocol | **Yoward** L, Bailey T, Doherty P, Edwards K, Purton J. A systematic review investigating the effectiveness of self-management interventions on balance, falling and walking for people who have multiple sclerosis. PROSPERO. 2016; CRD42016036067. Available from: https://www.crd.york.ac.uk/prospero/display_record.php?ID=CRD42016036067. |
| Protocol | **Yun** SJ, Shin HL, Kang MG, Oh BM, Seo HG. A systematic review and meta-analysis of the effect of robot-assisted gait training on gait and balance in patients with Parkinson disease. PROSPERO. 2018; CRD42018110009. Available from: https://www.crd.york.ac.uk/prospero/display_record.php?ID=CRD42018110009. |
| Protocol | **Ziebart** C, Babos P, Nazari G, MacDermid J, Dewan N. Assessing the quality of the literature and the efficacy of falls hazards programs: a meta-analysis and systematic review. PROSPERO. 2019; CRD42019133515. Available from: https://www.crd.york.ac.uk/prospero/display_record.php?ID=CRD42019133515. |
| Conference abstract | **Coehlo** M, Destri K, Fabbri M, Schrage A, Ferreira J, Consortium BC. Efficacy and safety of therapeutic interventions to treat motor symptoms in late stage Parkinson's disease: A systematic review. Movement Disorders. 2017;32:974-975. |
| Conference abstract | **Cranmer** S, Mester M, Palladino E, Hakim RM. Aquatic Therapy Compared With Conventional Land-Based Therapy to Improve Balance and Mobility in Persons With Parkinson's Disease: A Systematic Review. Journal of Aquatic Physical Therapy. 2018;26 1:39-40. |
| Conference abstract | **Dunlap** E, Lambeck J, Gobert D. Ai Chi for Balance, Gait Speed, Pain or Functional Outcomes in Adults: A Systematic Review. Journal of Aquatic Physical Therapy. 2018;26 2:28. |
| Conference abstract | **Keus** S. How to manage freezing and falling. Movement Disorders. 2010;25:S599. |
| Conference abstract | **Owen** C, Ibrahim K, Stack E, Dennison L, Roberts HC. What is the evidence of self-management interventions for people with parkinson's disease who fall: A systematic review. Age andAgeing. 2018;47:ii19. |
| Conference abstract | **Song** R, Grabowska W, Osypiuk K, Diaz GV, Bonato P, Park M, et al. Tai Chi's impact on motor and non-motor outcomes in Parkinson disease: A systematic review and meta-analysis. BMC Complementary and Alternative Medicine. 2017;17 DOI: 10.1186/s12906-017-1783-3. |
| Conference abstract | **Winser** S, Kannan P, Krishnamurthy K, Tsang W. Tai chi for balance and falls incidence in neurological disorders: A systematic review and meta-analysis. Archives of Physical Medicine and Rehabilitation. 2017;98 10:e134. |
| Conference abstract | **Winser** S, Lee SH, Law HS, Leung HY, Bello UM, Kannan P. Cost-effectiveness of physiotherapy interventions in neurological rehabilitation: a systematic review of economic evaluation. Physiotherapy (United Kingdom). 2019;105:e130-e131. |
| Did not meet systematic review criteria | **Abbruzzese** G, Marchese R, Avanzino L, Pelosin E. Rehabilitation for Parkinson’s disease: Current outlook and future challenges. Parkinsonism and Related Disorders. 2016;22:S60-S64. |
| Did not meet systematic review criteria | **Bloem** BR, Hausdirff JM, Visser JE, Giladi N. Falls and freezing of gait in Parkinson's disease: a review of two interconnected, episodic phenomena. Movement Disorders. 2004;19 8:871-884. |
| Did not meet systematic review criteria | **Boonstra** TA, van der Kooji H, Munneke M, Bloem BR. Gait disorders and balance disturbances in Parkinson's disease: clinical update and pathophysiology. Current Opinion in Neurology. 2008;21 4:461-471. |
| Did not meet systematic review criteria | **Coehlo** M, Ferreira J, Rosa M, Sampaio C. Treatment options for non-motor symptoms in late-stage Parkinson's disease. Expert Opinion on Pharmacotherapy. 2008;9 4:523-535. |
| Did not meet systematic review criteria | **Cucca** A, Biagioni MC, Fleisher JE, Agarwal S, Son A, Kumar P, et al. Freezing of gait in Parkinson’s disease: from pathophysiology to emerging therapies. Neurodegener Dis Manag. 2016;6 5:431-46. |
| Did not meet systematic review criteria | **Devlin** K, Alshaikh JT, Pantelyat A. Music Therapy and Music-Based Interventions for Movement Disorders. Current Neurology and Neuroscience Reports. 2019;19:83. |
| Did not meet systematic review criteria | **Fasano** A, Canning CG, Hausdorff JM, Lord S, Rochester L. Falls in Parkinson's disease: A complex and evolving picture. Movement Disroders. 32 11;1524-1536. |
| Did not meet systematic review criteria | **Feng** YS, Yang SD, Tan ZX, Wang MM, Xing Y, Dong F, et al. The benefits and mechanisms of exercise training for Parkinson’s disease. Life Sciences. 2020;245:117345. |
| Did not meet systematic review criteria | **Foster** ER, Bedeker M, Tickle-Degnen L. Systematic Review of the Effectiveness of Occupational Therapy-Related Interventions for People with Parkinson’s Disease. The American Journal of Occupational Therapy. 2014;68 1:39-49. |
| Did not meet systematic review criteria | **Gerards** MHG, McCrum C, Mansfield A, Meijer K. Perturbation-based balance training for falls reduction among older adults: Current evidence and implications for clinical practice. Geriatr Gerontol Int. 2017;17 12:2294-2303. |
| Did not meet systematic review criteria | **Goodwin** LS, Lan L. Evaluation and delivery of ambulatory rehabilitation for people with Parkinson’s disease. Reviews in Clinical Gerontology. 2014;24 2:122-138. |
| Did not meet systematic review criteria | **Hulbert** S, Rochester L, Nieuwboer A, Goodwin V, Fitton C, Chivres-Seymour K, et al. "Staying safe" – a narrative review of falls prevention in people with Parkinson's – "PDSAFE". 2019;41 21:2596-2605. |
| Did not meet systematic review criteria | **Mak** MKY, Wong-Yu ISK. Exercise for Parkinson’s disease. International Review of Neurobiology. 2019;147:1-44. |
| Did not meet systematic review criteria | **Müller** MLTM, Marusic U, van Emde Boas M, Weiss D, Bohnen NI. Treatment options for postural instability and gait difficulties in Parkinson’s disease. Expert Review of Neurotherapeutics. 2019;19 12:1229-1251. |
| Did not meet systematic review criteria | **Muthukrishnan** N, Abbas JJ, Shill HA, Krishnamurthi N. Cueing Paradigms to Improve Gait and Posture in Parkinson's Disease: A Narrative Review. Sensors. 2019;19 24:5468. |
| Did not meet systematic review criteria | **Nijkrake** MJ, Keus SHJ, Klaf JG, Sturkenboom IHWM, Munneke M, Kappelle AC, et al. Allied health care interventions and complementary therapies in Parkinson’s disease. Parkinsonism and Related Disorders. 2007;12:S488-S494. |
| Did not meet systematic review criteria | **Paul** SS, Dibble LE, Peterson DS. Motor learning in people with Parkinson’s disease: Implications for fall prevention across the disease spectrum. Gait & Posture. 2018;61;311-319. |
| Did not meet systematic review criteria | **Robin** ML, Stevens-Haas C, Havrilla E, Rosenstein A, Toffey B, Devi T, et al. Complementary Therapies for Parkinson's Disease: What's Promoted, Rationale, Potential Risks and Benefits. Movement Disorders Clinical Practice. 2015;2 3:205-212. |
| Did not meet systematic review criteria | **Rajan** P. Martial arts practice in community-based rehabilitation: A review. International Journal of Therapy and Rehabilitation. 2015;22 1: 31-34. |
| Did not meet systematic review criteria | **Taylor** MJD, Griffin M. The use of gaming technology for rehabilitation in people with multiple sclerosis. Multiple Sclerosis Journal. 2015;21 4:355-371. |
| Did not meet systematic review criteria | **Weerdesteyn** V, de Niet M, van Duijnhoven HJR, Geurts ACH. Falls in individuals with stroke. JRRD. 2008;45 8:1195-1214. |
| Old version of an included review | **Verheyden** GSAF, Weerdesteyn V, Pickering RM, Kunkel D, Lennon S, Geurts ACH, et al. Interventions for preventing falls in people after stroke. Cochrane Database of Systematic Reviews. 2015; DOI: 10.1002/14651858.CD008728.pub2. |
| Overlap | **Allen** NE, Sherrington C, Paul SS, Canning CG. Balance and falls in Parkinson's disease: A meta-analysis of the effect of exercise and motor training. Movement Disorders. 2011;26 9:1605-1615. |
| Overlap | **Alves da Rocha** P, McClell J, Morris M. Complementary physical therapies for movement disorders in parkinson's disease: a systematic review. European Journal of Physical and Rehabilitation Medicine. 2015;51 6:693-704. |
| Overlap | **Arnold** CM, Sran MM, Harrison EL. Exercise for fall risk reduction in community-dwelling older adults: a systematic review. Physiotherapy Canada. 2008;60 4:358-372. |
| Overlap | **Bowen** A, Hazelton C, Pollock A, Lincoln NB. Cognitive rehabilitation for spatial neglect following stroke. Cochrane Database of Systematic Reviews. 2013; DOI: 10.1002/14651858.cd003586.pub3. |
| Overlap | **Canning** CG, Paul SS, Nieuwboer A. Prevention of falls in Parkinson's disease: a review of fall risk factors and the role of physical interventions. Neurodegenerative Disease Management. 2014;4 3:203-221. |
| Overlap | **Carroll** LM, Morris ME, O’Connor WT, Clifford AM. Is Aquatic Therapy Optimally Prescribed for Parkinson's Disease? A Systematic Review and Meta-Analysis. Journal of Parkinson’s Disease. 2020;10:59-76. |
| Overlap | **Costantino** C, Petraglia F, Sabetta LL, Giumelli R. Effects of Single or Multiple Sessions of Whole Body Vibration in Stroke: Is There Any Evidence to Support the Clinical Use in Rehabilitation? Rehabilitation Research & Practice. 2018; DOI: 10.1155/2018/8491859. |
| Overlap | **Dibble** LE, Addison O, Papa E. The effects of exercise on balance in persons with Parkinson's disease: a systematic review across the disability spectrum. Journal of Neurologic Physical Therapy. 2009;33 1:14-26. |
| Overlap | **Fleischmann** C. The Parkinson's Patient: Use of Dynamic Neuromuscular Stabilization for Postural Alignment and Fall Risk Reduction. PhD Thesis. Azusa Pacific University, School of Behavioral and Applied Sciences; 2017. |
| Overlap | **García-Hermoso** A, Ramlrez-Vélez, de Asteasu, MLS, Martínez-Velilla N, Zambom-Ferraresi F, Valenzuela PL, et al. Safety and Effectiveness of Long-Term Exercise Interventions in Older Adults: A Systematic Review and Meta-analysis of Randomized Controlled Trials. Sports Medicine. 2020; DOI: 10.1007/s40279-020-01259-y. |
| Overlap | **Goodwin** VA, Abbott RA, Whear R, Bethel A, Ukuomunne O, Thompson-Coon J, et al. BMC Geriatrics. 2014;14:15. |
| Overlap | **Goodwin** VA, Richards SH, Taylor RS, Taylor AH, Campbell JL. The effectiveness of exercise interventions for people with Parkinson's disease: a systematic review and meta-analysis. Movement Disorders. 2008;23 5:631-640. |
| Overlap | **Gunn** H, Markevics S, Haas B, Marsden J, Freeman J. Systematic Review: The Effectiveness of Interventions to Reduce Falls and Improve Balance in Adults With Multiple Sclerosis. Archives of Physical Medicine & Rehabilitation. 2015;96 10:1898-1912. |
| Overlap | **Hill** KD, Hunter SW, Batchelor FA, Cavalheri V, Burton E. Individualized home-based exercise programs for older people to reduce falls and improve physical performance: A systematic review and meta-analysis. Maturitas. 2015;82 1:72-84. |
| Overlap | **Kwok** JYY, Chow CK, Lai CHY. Effects of mind–body exercises on the physiological and psychosocial well-being of individuals with Parkinson’s disease: A systematic review and meta-analysis. Complementary Therapies in Medicine. 2016;29:121-131. |
| Overlap | **Lee** MS, Lam P, Ernst E. Effectiveness of tai chi for Parkinson’s disease: A critical review. Parkinsonism and Related Disoders. 2008;14:589-594. |
| Overlap | **Ling-Rong** L, Meizhen H, Lam FMH, Pang MYC. Effects of Whole-Body Vibration Therapy on Body Functions and Structures, Activity, and Participation Poststroke: A Systematic Review. Physical Therapy. 2014;94 9:1232-1251. |
| Overlap | **Liu** HH, Yeh NC, Wu YF, Yang YR, Wang RY, Cheng FY. Effects of Tai Chi Exercise on Reducing Falls and Improving Balance Performance in Parkinson's Disease: A Meta-Analysis. Parkinsons Dis. 2019; DOI: 10.1155/2019/9626934. |
| Overlap | **Lötzke** D, Ostermann T, Büssing A. Argentine tango in Parkinson disease – a  systematic review and meta-analysis. BMC Neurology. 2015;15:226. |
| Overlap | **Lyu** D, Lyu X, Zhang Y, Ren Y, Yang F, Zhou L, et al. Tai Chi for Stroke Rehabilitation: A Systematic Review and Meta-Analysis of Randomized Controlled Trials. Front Physiol. 2018; 9:983 DOI: 10.3389/fphys.2018.00983. |
| Overlap | **Mansfield** A, Wong JS, Bryce J, Knorr S, Patterson KK. Does Perturbation-Based Balance Training Prevent Falls? Systematic Review and Meta-Analysis of Preliminary Randomized Controlled Trials. Physical Therapy. 2015;95 5:700-709. |
| Overlap | **Ni** X, Liu S, Lu F, Fuchang S, Xiaogeng GX. Efficacy and Safety of Tai Chi for Parkinson's Disease: A Systematic Review and Meta-Analysis of Randomized Controlled Trials. PLoS ONE. 2014;9 6:e99377. |
| Overlap | **Pollock** A, Hazelton C, Henderson CA, Angilley J, Dhillon B, Langhorne P, et al. Interventions for visual field defects in patients with stroke. Cochrane Database for Systematic Reviews. 2011; DOI: 10.1002/14651858.CD008388.pub2. |
| Overlap | **Rae-Grant** AD, Turner AP, Sloan A, Miller D, Hunziker J, Haselkorn JK. Self-management in neurological disorders: Systematic review of the literature and potential interventions in multiple sclerosis care. Journal of Rehabilitation Research & Development. 2011; 48 9:1087-1099. |
| Overlap | **Robinson** AG, Dennett AM, Snowdon DA. Treadmill training may be an effective form of task-specific training for improving mobility in people with Parkinson's disease and multiple sclerosis: a systematic review and meta-analysis. Physiotherapy. 2019;105 2:174-186. |
| Overlap | **Sherrington** C, Michaleff ZA, Fairhall N, Paul SS, Tiedemann A, Whitney J, et al. Exercise to prevent falls in older adults: an updated systematic review and meta-analysis. BJSM. 2017;51 24:1750-1758. |
| Overlap | **Toh** SFM. A Systematic Review on the Effectiveness of Tai Chi Exercise in Individuals with Parkinson’s Disease from 2003 to 2013. Hong Kong Journal of Occupational Therapy. 2013;23:69-81. |
| Overlap | **Tomlinson** CL, Patel S, Meek C, Herd CP, Clarke CE, Stowe R, et al. Physiotherapy intervention in Parkinson’s disease: Systematic review and meta-analysis. BMJ. 2012;345 7872:e5004. |
| Overlap | **Van Peppen** RPS, Kwakkel G, Wood-Dauphinee S, Hendriks HJM, Van der Wees PHJ, Dekker J. The impact of physical therapy on functional outcomes after stroke: what's the evidence? Clinical Rehabilitation. 2004; 18:833-862. |
| Overlap | **Winkel** A, Ekdahl C, Gard G. Early discharge to therapy-based rehabilitation at home in patients with stroke: a systematic review. Physical Therapy Reviews. 2008;13 3:167-187. |
| Overlap | **Wu** S, Chen J, Wang S, Jiang M, Wang X, Wen Y. Effect of Tai Chi Exercise on Balance Function of Stroke Patients: A Meta-Analysis. Med Sci Monit Basic Res. 2018;24:210-215. |
| Overlap | **Yitayeh** A, Teshome A. The effectiveness of physiotherapy treatment on balance dysfunction and postural instability in persons with Parkinson's disease: a systematic review and meta-analysis. BMC Sports Science, Medicine & Rehabilitation. 2016;8 17: DOI: 10.1186/s13102-016-0042-0. |
| Overlap | **Zhou** H, Yin T, Gao Q, Yang XC. A Meta-Analysis on the Efficacy of Tai Chi in Patients with Parkinson’s Disease between 2008 and 2014. Evidence-Based Complementary and Alternative Medicine. 2015; DOI: 10.1155/2015/593263. |
