# Supplementary material for: Effectiveness of interventions to prevent falls for people with multiple sclerosis, Parkinson’s disease and stroke: an umbrella review
Source: BMC Neurol. 2021 Sep 29;21:378. doi: 10.1186/s12883-021-02402-6 (PMC8480085; doi:10.1186/s12883-021-02402-6)
Supplement: Supplementary file 3 — Additional file 3. Characteristics of included reviews. [file 12883_2021_2402_MOESM3_ESM.docx]

**Effectiveness of interventions to prevent falls for people with Multiple Sclerosis, Parkinson’s Disease and stroke: An umbrella review**

**Authors**

Nicola O’Malley^1,2^, Amanda M. Clifford^1,2^, Mairéad Conneely^1,2^, Bláthín Casey^3,4^ and Susan Coote^1,4,5^

***Author’s Affiliations:***

^1^School of Allied Health, Faculty of Education and Health Sciences, University of Limerick, Ireland.

^2^Ageing Research Centre, Health Research Institute, University of Limerick, Ireland.

^3^Department of Physical Education and Sport Sciences, Faculty of Education and Health Sciences, University of Limerick.

^4^Centre of Physical Activity for Health, Health Research Institute, University of Limerick, Limerick, Ireland.

^5^Multiple Sclerosis Society of Ireland.

**Corresponding Author**

Name: Nicola O'Malley

Postal address: School of Allied Health, Faculty of Education and Health Sciences, University of Limerick, Ireland.

Phone number: +353 61 234118

Email: [Nicola.OMalley@ul.ie](mailto:Nicola.OMalley@ul.ie)

**Supplementary File 3:** Characteristics and findings of included systematic reviews.

| **Citation** | **Sosnuff & Sung (2015)** | **Booth et al. (2014)** |
| --- | --- | --- |
| **Neurological condition** | MS | MS |
| **Aim of systematic review** | To review the effects of falls prevention interventions on falls incidence among people with MS and determine characteristics of these programmes that might optimise the reduction of falls. | To evaluate whether virtual reality interventions, including interactive gaming systems, are effective at improving balance in adults with impaired balance. |
| **Search methods** | 4 databases (MEDLINE, Web of Science, CINAHL, SCOPUS)  Databases searched in January and February 2015.  English language only. | 9 databases (MEDLINE, CINAHL, EMBASE, AMED, CENTRAL, PsychINFO, PsychBITE, Otseeker, Ei Compendex, Inspec).  3 trial registries (CENTRAL, The Current Controlled Trials electronic database, The National Institute of Health Clinical Trials Database).  Databases searched up until November 2012.  No language restrictions. |
| **Number of relevant primary studies** | 9 primary studies:  3x RCTs  1x Non-RCT  1x Randomised two-period cross-over x1  1x Matched control trial x1  1x Multicentre, single-blinded, single-group, pretest-posttest trial x1  1x Single-blind, RCT x1  1x Randomised crossover trial | 1 primary study:  1x Multi-centre RCT |
| **Date range of relevant primary studies** | 2007-2015 | 2012 |
| **Country of origin of relevant primary studies** | N/R | Sweden |
| **Participants** | n = 504 (range: 28 - 111 participants)  Percentage of female participants: N/R  EDSS median range: 3.0-6.0 (Not reported x3 studies – 1x RCT, 2x NRSIs)  Mean age range: 46 - 63 years  Mixed types of MS x4 studies (3x RCTs, 1x NRSI)  SPMS only x2 RCTs  RRMS only x1 RCT  Did not outline type of MS included x2 NRSIs | n = 80  Percentage of female participants: N/R  Age: N/R  Did not outline type of MS |
| **Setting** | N/R | Hospital- or community-based patients |
| **Intervention (Type, Frequency, Intensity)** | Exercise-based x7 interventions:  Motor-sensory rehabilitation or motor rehabilitation x1 RCT  Group physiotherapy or 1-to-1 physiotherapy or yoga x1 NRSI  Wii balance board system training x1 RCT  Exergame training on an unstable platform or single-task exercises on the unstable surface x1 NRSI  Balance exercise targeting core stability, dual tasking and sensory strategies x1 NRSI  Home-based exercise or education or exercise and education x1 RCT  Sensory integration rehabilitation x1 RCT  Number of sessions range: 9 - 15 sessions (N/R by 3 studies: 2x RCTs, 1x NRSI)  Intervention duration range: 3 - 24 weeks  Technology-based x 2 interventions:  FES x1 RCT  FES for 12 weeks and exercise with FES for 12 weeks x1 RCT  Number of sessions: N/R x2 RCTs  Intervention duration range: 18 - 24 weeks | Nintendo WiiFit balance exercise programme  Session duration: 30 minutes  Session frequency: 2 sessions/week  Intervention duration: 6-7 weeks (12 sessions) |
| **Person delivering intervention** | Physiotherapist x1 NRSI  N/R x8 studies (4x RCTs, 4x NRSIs) | N/R |
| **Comparator** | Exercise-based interventions:  Conventional therapy x1 RCT  Conventional balance training x2 studies (1x RCT, 1x NRSI)  No treatment x3 studies 2x RCTs, 1x NRSI)  No control x1 NRSI  Technology-based interventions:  Conventional physiotherapy programme x 1RCT  Exercise for 12 weeks and exercise with FES for 12 weeks x1 RCT | No exercise |
| **Critical appraisal of included primary studies** | Customised checklist created based on the format of the Cochrane Methodological Quality Assessment Tool  Scores: 0 - not meeting the criterion, mentioned but unclear, or not mentioned; 1 - partially meeting the criterion; 3 - completely meeting the criterion  Total Score: 0-22  Score range for included studies: 5-16 | JBI Critical Appraisal Instrument  5/9 = Reasonable quality |
| **Type of analysis for falls outcomes** | Narrative synthesis | Narrative synthesis |
| **Follow-up period for relevant primary studies** | Exercise-based interventions:  Range: Immediately post-intervention - 6 months  Technology-based interventions:  Immediately post-intervention x2 RCTs | 2 months |
| **Falls outcomes** | Exercise-based interventions:  *Total number of falls:*  3x studies reported a significant reduction in the number of falls in the intervention groups (1x RCT, 2x NRSIs).  *Number of recurrent fallers:*  1x RCT reported a significantly lower number of recurrent fallers in the intervention groups compared to the control group.  *Number of fallers:*  2x RCTs reported that the number of fallers was lower following exercise-based intervention.  1x NRSI reported no difference in number of fallers between groups.  *Mean number of falls:*  1x NRSI reported that the intervention group had a lower mean number of falls than the control group.  Technology-based interventions:  *Total number of falls:*  1x RCT reported that the FES group had fewer falls than the conventional physiotherapy group.  1x RCT reported that groups using FES alone or in combination with physiotherapy demonstrated a reduction in falls. | *Total number of falls:*  1x RCT identified that those in the intervention group experienced less falls (n = 10) compared with the control group (n = 14) during the study. |
| **Secondary outcomes** | Exercise-based interventions:  *Balance:*  3x studies reported significant improvements in BBS for the exercise group (2x RCTs, 1x NRSI).  1x RCT reported significant improvement in SOT for the exercise group.  1x NRSI reported a significant improvement in FSST for the exercise group.  1x RCT reported no significant improvement in ABC scale for the exercise group.  1x RCT reported no significant improvement in the DHI for the exercise group.  *Mobility:*  1x NRSI reported significant improvements in TUG for the exercise group.  1x RCT reported no significant improvement in DGI for the exercise group.  *Physiological Falls Risk:*  1x RCT reported a 20% reduction in PPA score for the exercise groups.  *Fatigue:*  1x RCT reported a significant reduction in FSS for the exercise group  Technology-based interventions:  *Mobility:*  1x RCT reported an improvement in the Rivermead Observational Gait Analysis for the groups receiving FES alone or with physiotherapy. | *TUG:*  1x RCT found no significant difference in TUG when virtual reality was compared to no exercise.  *Adverse events:*  1x RCT reported dizziness as an adverse event during the intervention. |
| **Conclusions** | A detailed review of the literature suggests that fall incidence in people with MS may be reduced with exercise-based interventions and/or FES.  However, significant methodological issues preclude clear conclusions. | Evidence from current literature can neither support nor reject the inclusion of virtual reality training in clinical practice. |
| **Comments** | Data Collection: Falls recorded prospectively with a diary x7 studies (5x RCTs, 2x NRSIs), Falls recorded using retrospective recall x2 studies (1x RCT, 1x NRSI).  Fall definition: Any event that led to unexpected contact with support surface x1 RCT, An unexpected event in which the patient inadvertently came to rest on the ground or lower level and not due to a major intrinsic event x1 RCT, An unexpected contact of any part of the body with the ground x2 studies (1x RCT, 1x NRSI), An unexpected contact of any part of the body with the ground or lower level due to loss of balance x1 NRSI, An event in which the participant unintentionally came to rest on the ground or lower level x1 RCT, Losing one's balance so that the person came to rest on a lower surface x1 RCT, No definition provided x2 studies (1x RCT, 1x NRSI).  Faller classifications: Recurrent faller - more than one fall x1 RCT, Faller - individual who experienced a fall in the previous 3 months x1 RCT, Non faller - person who did not fall x1 RCT. | Falls were a primary outcome in this review but only one included study assessed this outcome.  Not reported if difference in number of falls between groups was significant.  Fall definition: N/R.  Methods of falls data collection: N/R . |

| **Citation** | **Hayes et al. (2019)** | **Denissen et al. (2019)** |
| --- | --- | --- |
| **Neurological condition** | MS | Stroke |
| **Aim of systematic review** | To evaluate the effectiveness of interventions to reduce falls in people with MS, specifically to compare falls prevention interventions to controls and to compare different types of falls prevention interventions. | To evaluate the effectiveness of interventions aimed at preventing falls in people after stroke.  Primary - to determine the effect of interventions on the rate of falls and the number of fallers.  Secondary- to determine the effects of interventions aimed at preventing falls on the number of fall-related fractures, the number of fall-related hospital admissions, near-fall events, economic evaluation, quality of life and adverse effects of the interventions. |
| **Search methods** | 6 databases (MEDLINE, Embase, CINAHL, LILACS, PsycINFO, PEDro).  4 trial registries (Trials Register of the Cochrane Multiple Sclerosis and Rare Diseases of the CNS Group, ClinicalTrials.gov, WHO International Clinical Trials Registry Platform, CENTRAL).  Databases searched up until December 2018.  No language restrictions. | 5 databases (MEDLINE, CINAHL, PsycINFO, AMED, PEDro).  7 trial registries (Trial Register of the Cochrane Stroke Group,  Trial Register of the Cochrane Bone, Joint and Trauma Group, CENTRAL, ClinicalTrials.gov, International Clinical Trials Registry Platform Search Portal, ISRCTN Registry, Stroke Trials Registry).  Databases searched up until September 2018.  No language restrictions |
| **Number of relevant primary studies** | 13 primary studies:  2x Multi-centre single-blinded RCTs  3x RCTs  1x Multi-centre single-blinded block RCT  2x Single-blinded block RCTs  1x Single-blinded stratified RCT  2x Single-blinded randomised cross-over trials  1x Single-blinded randomised controlled pilot trial  1x Single-blinded RCT | 14 primary studies:  14x RCTs |
| **Date range of relevant primary studies** | 2001-2017 | 2002-2018 |
| **Country of origin of relevant primary studies** | N/R | Australia x5 RCTs  Canada x2 RCTs  UK x2 RCTs  Brazil x1 RCT  Italy x1 RCT  Hong Kong x1 RCT  Sweden x1 RCT  USA x1 RCT |
| **Participants** | n = 839 (range: 12 - 177 participants)  Mean age = 52 years (range: 36 - 62 years)  Percentage of female participants ranged from 59-98%  Participants were diagnosed using the McDonald criteria x3 RCTs  Did not report the criteria used to diagnose MS, but reported participants were diagnosed with clinically definite MS x8 RCTs  Did not outline criteria used to confirm MS diagnosis x 2 RCTs  Mixed types of MS x9 RCTs  SPMS only x2 RCTs  RRMS only x1 RCT  Did not outline type of MS included x1 RCT  Participants in the majority of RCTs included people with mild to moderate severity of MS  Some RCTs recruited people being treated in hospital clinics, while the majority included people living in the community  None of the RCTs excluded people based on their falls history | n = 1358 (range: 34-170, median: 91)  All studies included both men and women; mean percentage of female participants; 40% (range: 29-65%)  Mean age range: 57 (+/-11) - 79 (+/-8) years  Mean age <60 years x1 RCT  Mean age 60-69 years x7 RCTs  Mean age ≥ 70 years x6 RCTs  Phase of stroke: chronic phase of stroke x4 RCTs, early and late subacute and chronic phases of stroke x1 RCT, late subacute and chronic phases of stroke x1 RCT, N/R x8 RCTs  Note:  Hyperacute phase = 24 hours post-stroke  Acute phase = 1-7 days post-stroke  Early subacute phase = 1 week - 3 months  Late subacute phase = 3-6 months  Chronic phase > 6 months |
| **Setting** | Community setting | Community or outpatient rehabilitation setting x10 RCTs  Institutional or hospital-based setting x4 RCTs |
| **Intervention (Type, Frequency, Intensity)** | Group-based exercise session (core stability exercise, dual-tasking and sensory strategies, individualised and progressed) or individualised HEP x1 RCT  One-to-one motor and sensory rehabilitation (strategies that challenged the motor and sensory system to maintain equilibrium with biofeedback) or motor rehabilitation (strategies that challenged the motor system to maintain equilibrium with feedback) x1 RCT  Balance treatment (to improve control of posture, moving centre of mass and body segments during static, dynamic and transitional tasks) x1 RCT  Group-based exercise circuit class (6 strength and balance exercises) or one-to-one physiotherapy (focus was exercise to improve balance and strength) or yoga classes (focus on yoga postures, stretching, breathing, meditation and relaxation exercises) x1 RCT  FES (wear oddstock drop foot stimulator daily for mobility) x1 RCT  Supervised sensory integration balance training x1 RCT  Interactive exergames (requiring stepping in multiple directions to cues) x1 RCT  Group-based exercise (functional mobility) x1 RCT  Wii Fit Plus balance games x1 RCT  Progressive HEP (to improve balance, walking, lower limb and trunk strength, and spasticity) x1 RCT  Exercise (HEP - balance, lower limb and trunk strength, and stretching) or education (group education involving self-management and self-efficacy enhancement techniques) or combined exercise and education x1 RCT  Activity Through Movement (class based on the principles of this approach to developing functional movement awareness progressively through practice of tasks) x1 RCT  FES (wear oddstock drop foot stimulator, addition of gluteal stimulation after 6 weeks and core stability exercises after 12 weeks) x1 RCT  Session duration range: 30 minutes - 4 hours  Session frequency range: 1-5 times/week  Intervention duration range: 3 weeks - 12 weeks (note: duration of intervention for x11 RCTs ranged from 6-12 weeks) | Exercise-based interventions x8 RCTs:  Treadmill training without body weight support (structured to increase step length, speed, balance, fitness, and automaticity) plus overground walking x1 RCT  Treadmill walking with harness for support plus conventional stroke rehabilitation x1 RCT  WEBB programme (circuit-style group exercise class involving task-related training with progressive balance and strengthening exercises as well as walking and stair climbing) plus HEP plus advice to increase walking x1 RCT  Physiotherapy treatment by an established community physiotherapy service as part of their usual work (participants treated with a problem-solving approach) x1 RCT  Whole-body vibration (comprised dynamic movements - weight shifting, squats, single leg squats, forward lunge) x1 RCT  External perturbation training (up to 60 pushes or pulls from supervising physiotherapist) x1 RCT  Exercise programme challenging dynamic balance and emphasising agility and multisensory approach in between (tasks included standing in various postures, walking with various challenges, STS movement, rapid knee raises in standing and standing perturbations - eyes closed and foam surfaces were incorporated for many of the tasks) x1 RCT  Tai Chi group (Yang-style 24-posture short form) or SilverSneakers group (fitness programme for older adults that offers different types of group-based exercise classes - aerobics, strength and ROM, water aerobics, yoga) x1 RCT  Session duration range: 30 - 60 minutes (N/R x2 RCTs)  Session frequency range: 1 - 3 sessions/ week (N/R x3 RCTs)  Intervention duration range: 6 weeks - 12 months  Environment/assistive technology x3 RCTs:  Predischarge home assessment visit (with an OT to identify and address any potential problems in the home environment) x1 RCT  Examination by an optometrist with prescription for a pair of single lens distance glasses x1 RCT  Walking training using the I-Walker plus exercises on hand recovery, tone control and improvement of global ability x1 RCT  I-Walker session frequency: 5 sessions/week  I-Walker intervention duration: 4 weeks  Other interventions/procedures x1 RCT:  Active repeated tDCS (anodal or bilateral or cathodal) plus physical rehabilitation programme x1 RCT  Session duration: N/R  Session frequency: 5 consecutive days/week  Intervention duration: 2 weeks  Multifactorial intervention x1 RCT:  Multifactorial, individually-tailored falls prevention programme (individualised HEP based on the Otago Exercise Program, falls risk minimisation strategies, written and verbal education about falls risk factors and risk minimisation, injury risk minimisation strategies and a falls prevention booklet) plus usual care after discharge (PT, OT, GP follow-up) x1 RCT  Intervention duration: 12 months  Multiple intervention x1 RCT:  HIFE programme (individualised exercise programmes including physical activity and functional performance, aimed at improving lower limb strength, balance and gait ability; implementing the functional exercises into real-life sessions; educational sessions with discussions about the increased risk of complications after stroke, such as falls) plus individualised HEP x1 RCT  Session duration: 45 minutes exercise, 45 minutes implementing exercises into real-life situations, 1 hour education  Session frequency: exercise 6 sessions/week, education 1 session/week  Intervention duration: 5 weeks followed by 3 months of HEP |
| **Person delivering intervention** | PT x4 RCTs  Experienced therapist x1 RCT  Experienced physical therapist x1 RCT  PT or yoga instructor x1 RCT  Guild Certified Feldenkrais practitioners x1 RCT  N/R x5 RCTs | PT x4 RCTs  Optometrist x1 RCT  OT x1 RCT  N/R x8 RCTs |
| **Comparator** | Waitlist controls x5 RCTs  Conventional therapy not directly targeted at balance improvements x1 RCT  Treatment that reduce limitations of body function and activity levels, with maximum 10 minutes of balance x1 RCT  Usual care x2 RCTs  HEP (individualised to improve gait and strength) x1 RCT  Supervised mobilisation, stretching and strengthening x1 RCT  Weekly call with structured questions about balance and mobility x1 RCT  Group education (topics of acupuncture, exercise, social support and dealing with MS) x1 RCT  3x RCTs with active comparator report same dose as for intervention | Exercise-based interventions:  No intervention x2 RCTs  Assisted overground walking plus conventional stroke rehabilitation x1 RCT  Exercise class designed to improve upper limb function, manage upper limb contracture with task-related strength and co-ordination training, and improve cognition with matching, sorting and sequencing tasks) plus HEP x1 RCT  Dynamic exercises (weight shifting, squats, single leg squats, lunges) on a platform with no vibration x1 RCT  Keep Moving with Stroke Programme (an exercise programme to enhance balance and mobility) x1 RCT  Stretching/weight-shifting x1 RCT  Provision of written materials and resources for participating in community-based physical activity suitable for older adults, which they could contact on their own, and weekly phonecalls to inquire of their health status x1 RCT  Environment/assistive technology studies:  Predischarge home assessment with an OT in the hospital (potential problems were discussed in general terms) x1 RCT  Examination by an optometrist x1 RCT  Overground ambulation exercises on the parallel bars plus exercises on hand recovery, tone control and improvement of global ability x1 RCT  Other interventions/procedures:  Sham transcranial direct current stimulation plus physical rehabilitation programme x1 RCT  Multifactorial interventions:  Falls prevention booklet plus usual care after discharge (PT, OT and GP follow-up) x1 RCT  Multiple interventions:  Educational sessions with group discussions about a variety of topics (fatigue, depression, dysphagia, etc) but with no special focus on the risk of falls x1 RCT |
| **Critical appraisal of included primary studies** | Cochrane Risk of Bias Tool  The risk of bias in the included RCTs was generally mixed.  Many of the included RCTs were judged to have an unclear risk of bias, mainly owing to inadequate reporting of methods used.  The majority of RCTs had low risk of bias in the domains for random sequence generation, blinding of outcome assessment and incomplete outcome data.  All included RCTs received unclear risk of bias for blinding of participants and personnel.  7x RCTs received high risk of bias for selective reporting.  9x RCTs demonstrated high risk of bias for various methodological shortcomings. | Cochrane Risk of Bias Tool with addition of one item: reliable ascertainment of fall/fallers outcome where 'low risk of bias' means ascertainment of outcome via active registration, e.g. falls diary; 'high risk of bias' if ascertainment relied on participants' recall over a longer period of time (more than one month); and 'unclear risk of bias' if ascertainment relied on participants' recall over a short period of time (one month or less) or if method of ascertainment was not described.  For five out of six of the items for 'risk of bias' assessment, the majority of included studies scored as having low risk of bias.  Only for blinding of outcome assessment did the majority of included studies score as having high risk of bias (x12 RCTs).  1x RCT scored low risk of bias in all six items. |
| **Type of analysis for falls outcomes** | Random- and fixed-effects meta-analyses | Random-effects meta-analyses |
| **Follow-up period for relevant primary studies** | Range: 1 - 6 months (3x RCTs included follow-up periods)  Immediately post-intervention x13 RCTs | Range: Immediately post-intervention - 12 months  4x RCTs registered falls data during the intervention period only  3x RCTs registered falls during the follow-up period only  7x RCTs registered falls during the intervention and follow-up period  Fall registration time ranged from 1 - 13 months (mean (SD): 8.07 (4.12) months) |
| **Falls outcomes** | Exercise versus control:  *Falls rate:*  RaR = 0.68  95% CI = [0.43, 1.06]  I^2^ = 58.61%  Test for overall effect: Z = 1.7  p = 0.09  5 RCTs informing outcome, n = 399  GRADE: Very low  There was no significant effect of exercise compared to control on falls rate post-intervention.  *Number of fallers:*  RR = 0.85  95% CI = [0.51, 1.43]  I^2^ = 44.87%  Test for overall effect: Z = 0.61  p = 0.79  5 RCTs informing outcome, n = 355  GRADE: Low  There was no significant effect of treatment on the number of fallers post-intervention.  *Number of fallers:*  RR = 1.16  95% CI = [0.78, 1.73]  1 RCT informing outcome, n = 177  There was no evidence of effect of exercise on number of fallers at 3-month follow-up.  *Number of fallers:*  RR = 1.02  95% CI = [0.69, 1.52]  1 RCT informing outcome, n = 177  There was no evidence of effect of exercise on number of fallers at 6-month follow-up.  Education versus control:  *Number of fallers:*  RR = 0.83  95% CI = [0.40, 1.76]  1 RCT informing outcome, n = 18  There was no evidence of effect in favour of either group post-intervention.  Exercise plus education versus control:  *Number of fallers:*  RR = 0.30  95% CI = [0.04, 2.20]  1 RCT informing outcome, n = 18  There was no evidence of effect in favour of either group post-intervention.  *Individual exercise versus control:*  Falls rate:  RaR = 4.5  95% CI = [0.99, 19.48]  1 RCT informing outcome, n = 64  There was evidence of an effect in favour of the control group post-intervention.  *Number of fallers:*  RR = 2.11  95% = [0.51, 8.74]  1 RCT informing outcome, n = 64  There was no evidence of effect in favour of either group post-intervention.  Yoga versus control:  *Falls rate:*  RaR = 4.67  95% CI = [0.99, 20.99]  1 RCT informing outcome, n = 28  There was no evidence of effect in favour of either group post-intervention.  FES versus exercise:  *Falls rate:*  RaR = 0.91  95% CI = [0.78, 1.06]  I^2^ = 53.76%  Test for overall effect: Z = 1.22  p = 0.22  2 RCTs informing outcome, n = 89  There was no evidence of effect in favour of either group post-intervention.  Exercise versus education:  *Falls rate:*  RaR = 0.71  95% CI = [0.39, 1.28]  1 RCT informing outcome, n = 12  There was no evidence of effect in favour of either group post-intervention.  *Number of fallers:*  RR = 0.49  95% CI = [0.16, 1.52]  1 RCT informing outcome, n = 20  There was no evidence of effect in favour of either group post-intervention.  Exercise versus exercise plus education:  *Number of fallers:*  RR = 0.73  95% CI = [0.20, 2.71]  1 RCT informing outcome, n = 19  There was no evidence of effect in favour of either group post-intervention.  Education versus exercise plus education:  *Number of fallers:*  RR = 2.08  95% CI = [0.30, 14.55]  1 RCT informing outcome, n = 17  There was no evidence of effect in favour of either group post-intervention.  Sensory integration balance training versus conventional rehabilitation:  *Falls rate:*  RaR = 0.10  95% CI = [0.01, 0.67]  1 RCT informing outcome, n = 80  There was evidence of an effect in favour of the sensory integration balance training group post-intervention.  Motor and sensory balance rehabilitation versus motor balance rehabilitation:  *Falls Rate:*  RaR = 6.00  95% CI = [0.38, 95.93]  1 RCT informing outcome, n = 34  There was no evidence of effect in favour of either group post-intervention.  *Number of fallers:*  RR = 0.48  95% CI = [0.03, 6.96]  1 RCT informing outcome, n = 34  There was no evidence of effect in favour of either group post-intervention.  Motor and sensory balance rehabilitation versus conventional rehabilitation:  *Falls rate:*  RaR = 3.00  95% CI = [0.31, 28.84]  1 RCT informing outcome, n = 38  There was no evidence of effect in favour of either group post-intervention.  *Number of fallers:*  RR = 1.05  95% CI = [0.49, 2.25]  1 RCT informing outcome, n = 119  There was no evidence of effect in favour of either group post-intervention.  *Number of fallers:*  RR = 9.46  95% CI = [1.31, 68.38]  1 RCT informing outcome, n = 119  There was evidence of an effect in favour of conventional rehabilitation at the 2-month follow-up.  Motor balance rehabilitation versus conventional non balance rehabilitation:  *Falls rate:*  RaR = 0.50  95% CI = [0.05, 4.81]  1 RCT informing outcome, n = 24  There was no evidence of effect in favour of either group post-intervention.  *Number of fallers:*  RR = 1.20  95% CI = [0.07, 21.72]  1 RCT informing outcome, n = 24  There was no evidence of effect in favour of either group post-intervention.  Group exercise versus yoga:  *Falls rate:*  RaR = 0.75  95% CI = [0.34, 1.66]  1 RCT informing outcome, n = 82  There was no evidence of effect in favour of either group post-intervention.  *Number of fallers:*  RR = 0.87  95% CI = [0.21, 3.56]  1 RCT informing outcome, n = 82  There was no evidence of effect in favour of either group post-intervention.  Group exercise versus individual exercise:  *Falls rate:*  RaR = 1.00  95% CI = [0.54, 1.85]  1 RCT informing outcome, n = 111  There was no evidence of effect in favour of either group post-intervention.  *Number of fallers:*  RR = 0.70  95% CI = [0.27, 1.82]  1 RCT informing outcome, n = 111  There was no evidence of effect in favour of either group post-intervention.  Individual exercise versus yoga:  *Falls rate:*  RaR = 0.75  95% CI = [ 0.32, 1.74]  1 RCT informing outcome, n = 61  There was no evidence of effect in favour of either group post-intervention.  *Number of fallers:*  RR = 1.19  95% CI = [0.37, 3.77]  1 RCT informing outcome, n = 61  There was no evidence of effect in favour of either group post-intervention. | Exercise-based interventions:  *Fall rate:*  RaR = 0.72  95% CI = [0.54, 0.94]  I^2^ = 42.87%  8 RCTs informed this outcome, n = 765  Test for overall effect: Z = 2.37  p = 0.02  GRADE: Low  When pooled, there was a significant reduction in falls rate for the experimental group.    *Number of fallers:*  RR = 1.03  95% CI = [0.90, 1.19]  I^2^ = 0%  10 RCTs informed this outcome, n = 969  Test for overall effect: Z = 0.43  p = 0.67  GRADE: Very low  When pooled, there was no significant effect of exercise on number of fallers.  *Number of people sustaining fall-related fractures:*  1x RCT reported that a participant had a stroke, fractured his shoulder and died in hospital.  2x RCTs reported that no falls resulted in a fracture.  1x RCT reported that a participant in the intervention group sustained a hip fracture, but on a task that was included in both groups.  *Falls requiring medical attention:*  1x RCT reported that no falls required medical attention.  1x RCT reported three cases where participants sought medical attention for a fall.  1x RCT reported ten cases where participants sought medical attention for a fall.  Social environment interventions:  *Fall rate:*  RaR = 0.85  95% CI = [0.43, 1.69]  1 RCT informed this outcome, n = 85  Test for overall effect: Z = 0.45  p = 0.65  GRADE: Very low  There was no significant reduction in falls rate when comparing a home visit to a predischarge assessment in the hospital setting.  *Number of fallers:*  RR = 1.48  95% CI = [0.71, 3.09]  1 RCT informed this outcome, n = 85  Test for overall effect: Z = 1.04  p = 0.3  GRADE: Very low  There was no significant difference in number of fallers between the home visit or hospital assessment groups.  Aids for communication, information and signalling:  *Falls rate:*  RR = 1.08  95% CI = [0.52, 2.25]  1 RCT informing this outcome, n = 43  Test for overall effect: Z = 0.2  p = 0.84  GRADE: Very low  There was no significant reduction in falls rate when single lens distance vision glasses replaced multifocal glasses.  *Number of fallers:*  RR = 0.74  95% CI = [0.47, 1.18]  1 RCT informing this outcome, n = 43  Test for overall effect: Z = 1.25  p = 0.21  GRADE: Very low  There was no significant reduction in number of fallers when single lens distance vision glasses replaced multifocal glasses.  *Number of people sustaining fall-related fractures:*  1x RCT reported that nobody in the intervention group sustained a fracture, compared to one person in the control group.  *Number of people with fall-related hospital admissions:*  1x RCT reported that one person in the intervention group was admitted once, compared to one person in the control group who was admitted three times.  Aids for personal mobility:  *Falls rate:*  RaR = 0.56  95% CI = [0.19, 1.66]  1 RCT informing this outcome, n = 42  Test for overall effect: Z = 1.05  p = 0.29  GRADE: Very low  There was no significant reduction in falls rate for the I-walker group compared to the control group.  *Number of fallers:*  RR = 0.44  95% CI = [0.16, 1.22]  1 RCT informing this outcome, n = 42  Test for overall effect: Z = 1.57  p = 0.12  GRADE: Very low  There was no significant reduction in number of fallers for the I-walker group compared to the control group.  tDCS:  *Number of fallers:*  RR = 0.30  95% CI = [0.14, 0.63]  1 RCT informing this outcome, n = 60  Test for overall effect: Z = 3.17  p < 0.001  GRADE: Low  There was a significant reduction in the number of fallers in the active transcranial direct current stimulation group compared to the control group.  Furthermore, there was a significant reduction in number of fallers for all individual montages of active tDCS compared to the sham tDCS group. |
| **Secondary outcomes** | *Attrition rate:*  N/R x8 RCTs.  Dropouts ranged from 6-20% across the 5 RCTs for which this data were reported.  Exercise versus control:  *Adverse Events:*  No evidence of effect of the intervention x3 RCTs.  *Physiological Falls Risk:*  No evidence of effect in favour of either group on PPA x2 RCTs.  *Balance:*  There was evidence of an effect in favour of exercise with a SMD of 0.50 (95% CI 0.09 to 0.92) x5 RCTs (4 RCTs used BBS, 1 RCT used FSST).  No evidence of effect in favour of intervention on ABC Scale x1 RCT.  No evidence of effect in favour of intervention on standing postural sway x1 RCT.  *Psychological Measures:*  There was evidence of an effect in favour of the control group on MSSE Scale with a MD of -7.58 (95% CI -12.57 to -2.59) x1 RCT.  No evidence of effect in favour of intervention on MSIS-29 (psychological sub-component) x3 RCTs.  No evidence of effect in favour of intervention on FES-I x1 RCT.  *Cognition:*  There was evidence of an effect in favour of exercise on Stroop Testing Step Measure with a MD of 16.40 (95% CI 5.34 to 27.46) x1 RCT.  No evidence of effect in favour of exercise on TUG-C x1 RCT.  No evidence of effect in favour of exercise on SDMT x1 RCT.  No evidence of effect in favour of exercise on TMT Measure x1 RCT.  *Self-Reported Mobility:*  There was evidence of an effect favouring exercise on MSWS-12 with a MD of 16.30 (95% CI 9.34 to 23.26) x3 RCTs.  *Long Walking Measures of Mobility:*  No evidence of effect in favour of either group x4 RCTs (3 RCTs used 6MWT, 1 RCT used 2MWT).  *Short Walking Measure of Mobility:*  There was evidence of an effect in favour of exercise with a SMD of 0.28 (95% CI 0.07 to 0.50) x5 RCTs (T25FW and 10MWT).  No evidence of effect in favour of either group on TUG x3 RCTs.  *Functional Outcomes:*  No evidence of effect in favour of exercise on Barthel ADL Scale x1 RCT.  *Fatigue:*  No evidence of effect in favour of intervention x2 RCTs (1 RCT used MFIS, 1 RCT used FSMC).  Education versus control:  *Adverse Events:*  No significant effect of education on number of adverse events x1 RCT.  Physiological falls risk:  No evidence of effect in favour of intervention on PPA x1 RCT.  Exercise and education versus control:  *Adverse Events:*  No significant effect of exercise and education on number of adverse events x1 RCT.  *Physiological Falls Risk:*  No evidence of effect in favour of intervention on PPA x1 RCT.  Yoga versus control:  *Balance:*  No evidence of effect in favour of intervention on BBS x1 RCT.  *Psychological Measures:*  No evidence of effect in favour of intervention on MSIS-29 (psychological sub-component) x1 RCT.  *Fatigue:*  No evidence of effect in favour of intervention on MFIS x1 RCT.  Individual exercise versus control:  *Balance:*  There was evidence of an effect in favour of individual exercise on BBS with a MD of 12.40 (95% CI 6.33 to 18.47) x1 RCT.  *Psychological Measures:*  No evidence of effect in favour of intervention on MSIS-29 (psychological sub-component) x1 RCT.  *Fatigue:*  No evidence of effect in favour of intervention on MFIS x1 RCT.  FES versus exercise:  *Adverse Events:*  No significant effect of FES or exercise on number of people reporting adverse events x1 RCT.  *Psychological Measures:*  No evidence of effect in favour of either group on MSIS-29 x1 RCT.  *Mobility:*  No evidence of effect in favour of either group on 10MWT x1 RCT.  Exercise versus education:  *Adverse Events:*  No significant effect of exercise or education on number of people reporting adverse events x1 RCT.  *Physiological Falls Risk:*  No significant effect in favour of either group on PPA x1 RCT.  *Balance:*  No significant effect in favour of either group on the Computerised Balance Assessment x1 RCT.  No evidence of effect in favour of either group on ABC Scale x1 RCT.  *Psychological Measures:*  No significant effect in favour of either group on MSSE Scale x1 RCT.  Exercise plus education versus exercise:  *Adverse Events:*  No significant effect of exercise plus education or exercise on number of people reporting adverse events x1 RCT.  *Physiological Falls Risk:*  No significant effect of either group on PPA x1 RCT.  Exercise plus education versus education:  *Adverse Events:*  No significant effect of exercise plus education or education on number of people reporting adverse events x1 RCT.  *Physiological Falls Risk:*  No significant effect of either group on PPA x1 RCT.  Sensory integration balance training versus conventional rehabilitation:  *Quality of Life:*  No evidence of effect in favour of either group post-intervention on MSQOL-54 (mental component) x1 RCT.  No evidence of effect in favour of either group post-intervention on MSQOL-54 (physical component) x1 RCT.  There was evidence of an effect in favour of sensory integration balance training at 1-month follow-up on MSQOL-54 (physical component) with a MD of 5.02 (95% CI 0.2 to 9.82) x1 RCT.  *Balance:*  There was evidence of an effect in favour of sensory integration balance training on BBS with a MD of 4.98 (95% CI 2.88 to 7.08) x1 RCT, effect was maintained at 1-month follow-up.  There was evidence of an effect in favour of sensory integration balance training on ABC Scale with a MD of 8.97 (95% CI 0.94 to 17.00) x1 RCT, effect was maintained at 1-month follow-up.  *Fatigue:*  No evidence of effect in favour of either group on FSS x1 RCT.  Motor and sensory balance rehabilitation versus motor balance rehabilitation:  *Balance:*  No evidence of effect in favour of either group on BBS x1 RCT.  No evidence of effect in favour of either group on ABC Scale x1 RCT.  Motor and sensory balance rehabilitation versus conventional rehabilitation:  *Balance:*  No evidence of effect in favour of either group on BBS x1 RCT.  No evidence of effect in favour of either group on ABC Scale x2 RCTs.  *Mobility:*  No evidence of effect in favour of either group on DGI x2 RCTs.  No evidence of effect in favour of either group on TUG x1 RCT.  Motor balance rehabilitation versus conventional non balance rehabilitation:  *Balance:*  There was evidence of an effect in favour of motor balance rehabilitation on BBS with a MD of 6.75 (95% CI 1.09 to 12.41) x1 RCT.  No evidence of effect in favour of either group on ABC Scale x1 RCT.  *Mobility:*  No evidence of effect in favour of either group on DGI x1 RCT.  Group exercise versus yoga:  *Balance:*  There was evidence of an effect in favour of group exercise on BBS with a MD of 6.60 (95% CI 0.49 to 12.71) x1 RCT.  *Psychological Measures:*  No evidence of effect in favour of either group on MSIS-29 x1 RCT.  *Fatigue:*  No evidence of effect in favour of either group on MFIS x1 RCT.  Group exercise versus individual exercise:  *Balance:*  No evidence of effect in favour of either group on BBS x1 RCT.  *Psychological Measures:*  No evidence of effect in favour of either group on MSIS-29 x1 RCT.  *Fatigue:*  No evidence of effect in favour of either group on MFIS x1 RCT.  Individual exercise versus yoga:  *Balance:*  No evidence of effect in favour of either group on BBS x1 RCT.  *Psychological Measures:*  No evidence of effect in favour of either group on MSIS-29 x1 RCT. | Exercise-based interventions:  *QoL:*  14 different outcome measures of QoL were used across the included reviews, with the majority reporting no significant effect between the exercise and control groups.  3x RCTs used the AAP: 2x RCTs identified no significant effect favouring either group, 1x RCT identified a significant effect for the subscale 'service to others' favouring intervention and a significant effect for the subscale 'social activities' favouring control.  3x RCTs used the SF-12/SF-36: 2x RCTs identified no significant effect favouring either group, 1x RCT identified a significant effect for the subscales 'mental dimension' and 'mental health' favouring intervention at 3-month follow-up.  3x RCTs used the ABC Scale: No significant effect identified in favour of either group.  2x RCTs used the falls efficacy scale (Swedish/International): 1x RCT identified no evidence of effect in favour of either group, 1x RCT identified a significant effect in favour of intervention at 3-month follow-up.  2x RCTs used FAI: No significant effect identified in favour of either group.  1x RCT used SIPSO: Significant effect in favour of control identified at 6-,8-,10- and 12- month follow-ups.  1x RCT used EuroQol EQ-5D-3L: No significant effect identified in favour of either group.  1x RCT used Walking Self-Efficacy Scale: No significant effect identified in favour of either group.  1x RCT used HADS: No significant effect identified in favour of either group.  1x RCT used GHQ-28: No significant effect identified in favour of either group.  1x RCT used PASIPD: No significant effect identified in favour of either group.  1x RCT used NHP: No significant effect identified in favour of either group.  1x RCT used the CES-D: No significant effect identified in favour of either group.  1x RCT used PSQI: No significant effect identified in favour of either group.  *Adverse events:*  1x RCT reported that no falls or other adverse events occurred during the exercise class, home exercise programme or assessments but one participant withdrew stating that the exercises exacerbated an incontinence issue.  1x RCT reported that no severe adverse events occurred but three participants experience dizziness during whole-body vibration therapy and four had lower-limb soreness and fatigue (2 of these participants were in the intervention group), all symptoms gradually subsided after the first few sessions.  1x RCT reported involving perturbation training reported 48 adverse events: fatigue with training (n=4), joint pain during or soon after training (n=25), delayed onset muscle soreness (n=13), seizure during training (n=1), abnormally elevated heart rate and low blood pressure during training (n=1), and falls related to the study procedure (n=4).  Social environment interventions:  *QoL:*  1x RCT used SADQ-10: Mood significantly differed in favour of the home-visit group.  1x RCT used EQ-5D: No significant difference in favour of either group.  Aids for communication, information and signalling:  *QoL:*  1x RCT used SF-12 physical and mental components: No significant effect in favour of either group.  1x RCT used FES-I: No significant effect in favour of either group.  tDCS:  *Adverse events:*  1x RCT reported no adverse events. |
| **Conclusions** | The evidence regarding the effectiveness of non-pharmacological falls prevention interventions for people with MS is sparse and uncertain.  This review identified some evidence that exercise interventions resulted in improvements in balance and mobility; however, these were measured as secondary outcomes and this should be interpreted with caution.  There is a need for further high-quality research in this field, with standardisation of methods, outcomes and reporting required. | The evidence regarding the effectiveness of non-pharmacological falls prevention interventions for people with MS is sparse and uncertain.  This review identified low to very low quality evidence suggesting that exercises, either as a single intervention or part of a multi-component intervention, and including ambulation, perturbation/vibration based, balance/strength oriented or Tai-Chi, reduce falls rate but not the number of fallers among people post-stroke.  There was insufficient evidence to draw conclusions regarding treatment effects in a specific phase post-stroke.  There is a need for further high-quality research in this field, with standardisation of methods, outcomes and reporting required. |
| **Comments** | Reported that a range of definitions used for a fall in included studies; however, these definitions were not provided in review.  Prospective falls diaries used to collect falls data x8 RCTs.  Retrospective methods of collecting self-report of falls data x3 RCTs.  Did not outline how falls data was collected x2 RCTs.  No study reported data on cost-effectiveness. | Methods of falls data collection: 8x RCTs recorded falls prospectively using written records, 2x RCTs recorded falls using retrospective recall, 2x RCTs recorded falls using interviews (not reported if retrospective recall or participants were keeping a record), 2x RCTs reported insufficient detail regarding falls evaluation.  No studies included individuals in the hyperacute phase after stroke.  Fall definitions not provided but is reported that a standardised definition is required.  Faller classifications: N/R. |

| **Citation** | **Pollock et al. (2014)** | **Batchelor et al. (2010)** |
| --- | --- | --- |
| **Aim of systematic review** | Primary objectives:  To determine the effect of interventions that alter the starting posture on ability to STS independently.  To determine the effect of rehabilitation interventions on ability to STS independently.  Secondary objectives:  To determine the effects of interventions aimed at improving ability to STS, on time taken to STS, symmetry of weight distribution during STS, peak vertical ground reaction forces during STS, lateral movement of centre of pressure during STS and incidence of falls. | To systematically evaluate the effects of any interventions on falls in people after stroke. |
| **Search methods** | 12 databases (MEDLINE, EMBASE, CINAHL, AMED, British Nursing Index, REHABDATA, OTseeker, PEDro, CSP Research Database, OT Search, Dissertation abstracts, UK Clinical Research Network Portfolio Database).  8 trial registries (Current Controlled Trials, EU Clinical Trials Register, Stroke Trials Registry, WHO International Clinical Trials Registry Platform, Cochrane Stroke Trials Register, CENTRAL, ClinicalTrials.gov, National Research Register).  Databases searched up to June 2013.  No language restrictions. | 8 databases (Allied and Complementary Medicine, EMBASE, MEDLINE, PsycINFO, CINAHL, Ageline, PEDro, Cochrane Library).  2 searches of public bodies and guidelines (ProFANE, NICE).  Database search conducted in March 2009.  English language only. |
| **Number of relevant primary studies** | 3 primary studies:  1x RCT  1x Multicentre RCT  1x Randomised exploratory trial | 10x primary studies:  7x RCTs (note: 4x primary studies were based on one RCT with different follow-up periods) |
| **Date range of relevant primary studies** | 2001 - 2012 | 1990-2008 |
| **Country of origin of relevant primary studies** | Australia x1 RCT  UK x1 RCT  Taiwan x1 RCT | Canada x1 RCT  Australia x1 RCT  Taiwan x1 RCT  UK x1 RCT  USA x1 RCT  Japan x1 RCT  Sweden x1 RCT |
| **Participants** | n = 276 (range: 54 - 156)  Percentage of female participants: N/R  Mean age range (intervention group): 60 (+/-7) - 72 (+/-10.4) years  Mean time since stroke range (intervention group): 21 (+/-8) - 171 [range: 55-287] days  1x RCT included only participants who were already able to STS independently  1x RCT included only participants who were able to walk independently | n = 723 (range: 39 - 258)  Percentage of female participants: N/R x6 RCTs, 46% x1 RCT  Age = N/R x5 RCTs (range: 18 - 90 years x1 RCT, mean: 74.7 years x1 RCT)  Time since stroke range: <24 hours to >2 years post-stroke |
| **Setting** | Rehabilitation centre x1 RCT  Hospital-based rehabilitation setting x1 RCT  Home-based x1 RCT | Acute care x1 RCT  Inpatient rehabilitation x2 RCT  Community x2 RCTs  Residential/Institutional care x1 RCT  N/R x1 RCT |
| **Intervention (Type, Frequency, Intensity)** | Repetitive STS training (STS task performed as symmetrically as possible using visual and auditory feedback) plus standing postural symmetry training (stand in front of a height adjustable work table with a mirror and weight-bearing sensors to provide visual and auditory feedback, moving a weight loaded box while maintaining symmetry) plus conventional stroke rehabilitation (neuromuscular facilitation, FES and mat exercises) x1 RCT  Falls prevention programme (Multifactorial, individually tailored falls prevention programme consisting of individualised HEP based on Otago Exercise Programme, fall risk minimisation strategies, education for participant and carer about identified falls risk factors and risk minimisation and injury risk minimisation strategies) plus usual care by treating healthcare professionals (PT, OT and GP) x1 RCT  Exercise training including endurance and resistance exercises (cycle ergometry, raising and lowering an exercise ball, shuttle walking, standing chest press, stairs climbing and descending, upper back strengthening, triceps extension, pole lifting exercise, repetitive STS practice) x1 RCT  Session duration range: 30-50 min (not reported x1 RCT)  Session frequency range: 3-5 sessions/week  Duration of intervention range: 3 weeks-12 months | Group STS practice plus usual care x1 RCT  Very early mobilisation emphasising upright and out of bed plus usual care x1 RCT  Standing symmetry training and STS training (with biofeedback) plus usual care x1 RCT  Community physiotherapy sessions (gait re-education exercises, functional exercises, balance exercises) x1 RCT  Fresnel prisms applied to affected hemi-field worn for daytime activities plus usual rehabilitation including visual retraining x1 RCT  Sunlight exposure outdoors x1 RCT  Home rehabilitation (MDT outreach service with PT, OT, SLT and SW, and assignment of a case manager) x1 RCTs (note: included in 4 studies)  Duration of session range: 15 - 50 minutes/session  Frequency of intervention range: 3-12 sessions/week  Duration of intervention range: first 14 days after stroke or until discharge (whichever happened sooner) - 12 months  Note - interventions can be broadly classified as follows:  Physical activity interventions (including balance training, exercise, strength training) x4 RCTS  Environmental modification/improving knowledge intervention x1 RCT  Models of stroke care intervention x1 RCTs  Intervention to influence bone mineral density x1 RCT |
| **Person delivering intervention** | Not reported x2 RCTs  PT x1 RCT | PT x1 RCT  MDT (PT, OT, SLT, SW and case worker) x1 RCT  Not reported x5 RCTs |
| **Comparator** | Conventional stroke rehabilitation plus additional therapeutic exercises x1 RCT  Usual care plus provision of falls prevention booklet x1 RCT  Seated relaxation exercises focused on attention control x1 RCT | Recreational therapy plus usual care x1 RCT  Usual care (conventional stroke rehabilitation including neuromuscular facilitation, FES, mat exercises, other exercises) x1 RCT  Usual care (OT/nursing/PT) x1 RCT  No additional treatment x1 RCT  Usual rehabilitation including visual retraining x1 RCT  No additional sunlight exposure x1 RCT  Routine rehabilitation x1 RCT (note: included in 4 studies) |
| **Critical appraisal of included primary studies** | Cochrane Risk of Bias Tool  1x RCT low risk of bias for all methodological parameters assessed  1x RCT insufficient data to be certain of the risk of bias for several parameters  1x RCT low risk of bias for majority of parameters, unclear risk of bias for one parameter | PEDro  Range: 4-8  7 of the relevant primary studies scored ≤6, indicating a reasonably high level of bias  The main biases were lack of blinding of participants and therapists to the intervention, lack of allocation concealment, and lack of intention-to-treat analysis.  Note: not possible to blind participants and therapists to exercise interventions. |
| **Type of analysis for falls outcomes** | Sensitivity analysis of a fixed-effects meta-analysis | Meta-analysis |
| **Follow-up period for relevant primary studies** | Range: Immediately post-intervention - 12 months | Range: Immediately post-intervention - 5 years |
| **Falls outcomes** | *Number of fallers:*  OR = 0.81  95% CI = [0.35, 1.87]  3 RCTs informing outcome, n = 276  There was no evidence of an effect of intervention on the number of fallers compared to control. | Exercise versus usual care:  *Falls rate:*  RaR = 1.22  95% CI = [0.76, 1.98]  p value: N/R  I^2^: N/R  2 RCTs informing outcome, n = 119  There was no significant effect of exercise on fall rate.  *Number of fallers:*  RR = 0.77  95% CI = [0.24, 2.43]  p value: N/R  I^2^: N/R  2 RCTs informing outcome, n = 224  There was no significant effect of exercise on proportion of fallers.  Intervention versus control:  *Fall rate:*  No significant effect of the intervention on fall rate was found x6 RCTs  *Number of fallers:*  No significant effect of the intervention on number of fallers was found x6 RCTs. |
| **Secondary outcomes** | *Number of STS in specified time:*  SMD = 0.05  95% CI = [-0.29, 0.40]  Test for overall effect: Z = 0.31  p = 0.76  1 study informing outcome, n = 132  No significant effect of the intervention when compared with control on number of STS in specified time.  *Functional ability:*  SMD = -0.24  95% CI = [-0.52, 0.04]  I^2^ = 0%  Test for overall effect: Z = 1.66  p = 0.097  2 RCTs informing outcome, n = 196  GRADE: low  No significant effect of the intervention when compared with control on functional ability.  *Attrition rate:*  No dropouts reported x1 RCT.  144 (92%) had at least 1 month of falls data available for analysis, 12 additional participants (5 control, 7 intervention) did not complete the final assessment x1 RCT.  Number of dropouts N/R x1 RCT. | No secondary outcomes reported. |
| **Conclusions** | There was insufficient evidence to reach conclusions relating to the effect of interventions to improve sit-to-stand on falls outcomes among people post-stroke. | No significant effects for non-pharmacological interventions were found either individually or in pooled results for falls rate or number of fallers.  There is a need for commonality in the way falls are defined, measured, reported and analysed.  There is very little high-quality evidence on successful approaches to reducing falls among people post-stroke.  This review highlights the need for RCTs specifically aimed at reducing falls in stroke. |
| **Comments** | Fall definition: N/R.  Methods of falls data collection: N/R. | No studies evaluating multifactorial falls prevention intervention for people post-stroke were identified.  Falls data collection: no study prospectively recorded falls with a falls diary, of the community-based studies retrospective recall over varying time frames ranging from 3-12 months were used, for studies based in institutions falls data were collected by observation and report or from incident reports or hospital files in 2 studies, for four studies the method of falls data collection was not reported.  Outcomes: there were differences in outcomes used; the unit of analysis included number of falls, falls rate, number of fallers, and number of recurrent fallers.  Fall definition: one study included a specific definition for a fall (patient's knees, buttocks, or trunk unintentionally contacting the floor), eight studies provided no explicit definition, and one study did not define falls specifically but excluded falls that were the result of environmental factors (Such as slipping or tripping).  Faller classifications: N/R x2 studies, faller ≥ 2 falls in 6 months x1 study, faller ≥ 1 fall x1 study, recurrent/frequent faller > 1 fall x2 studies. |

| **Citation** | **Rutz et al. (2020)** | **Owen et al. (2019)** |
| --- | --- | --- |
| **Aim of systematic review** | To investigate the evidence for physical interventions for freezing of gait and gait impairments in PD and establish recommendations for clinical practice. | To identify and review falls self-management interventions for people with PD and, where possible, assess their efficacy for improving patient and caregiver outcomes, quality of life and psychological outcomes. |
| **Search methods** | 4 databases searched (Embase, PEDro, CINAHL, PubMed).  Databases searched from 1996 - April 2018.  English or French language only. | 6 databases searched (MEDLINE, EMBASE, CINAHL, AMED, PSYCHInfo, Science Citation Index Expanded).  Grey literature search (www.opengrey.eu).  Clinical trial registries searched (no specific trial registry stated).  Database search from 1986 to June 2018.  No language restrictions: |
| **Number of relevant primary studies** | 1 primary study:  1x RCT | 3 primary studies:  2x RCT  1x 3-arm RCT |
| **Date range of relevant primary studies** | 2015 | 2005-2017 |
| **Country of origin of relevant primary studies** | N/R | Australia x3 RCTs |
| **Participants** | n = 21  Percentage of female participants: N/R  Age: N/R  H&Y Range: II - III  All participants had freezing of gait | n = 574 (133-231)  Mean age range: 67.9 (+/- 9.6) - 71.4 (+/- 8.1) years  Majority participants H&Y stage ≤ 2 (indicating reduced falls risk) x2 RCTs  Range of participants that had fallen in year preceding intervention: 55-78%  Mean MMSE range: 28.2 (+/- 1.8) - 28.7 (+/- 1.4)  Mean years since diagnosis: 6.7 (+/- 5.6) - 8.3 (+/- 6) years (N/R x1 RCT) |
| **Setting** | N/R | Hospital-based x2 RCTs  Home-based x1 RCTs |
| **Intervention (Type, Frequency, Intensity)** | HEP with rhythmic auditory cueing (metronome) and functional walking exercises  Session duration: 30-60 minutes  Intervention duration: 6 months | Physiotherapy plus education x3 RCTs:  Physiotherapy (progressive balance and lower limb strengthening exercises and cueing strategies - exercise classes and physical therapist home visits) plus education (received booklet "Don't fall for it, falls can be prevented") x1 study  Physiotherapy (movement strategy training or progressive resistance strength training) plus education (elements of booklet "Don't fall for it, falls can be prevented" explained to each participant) x1 RCT  Physiotherapy (progressive strength training and movement strategy training) plus education (individualised falls education based on the booklet "Don't fall for it, falls can be prevented") x1 RCT  Session duration and frequency range for physiotherapy: 2 hours/week to 40-60 minutes 3 times/week  Session frequency for education: delivered once weekly x2 RCTs  Frequency of home visits: 2-4 x1 RCT  Intervention duration range: 6 weeks - 6 months |
| **Person delivering intervention** | N/R | Physical therapist led x3 RCTs |
| **Comparator** | 6 month waitlist control | Received booklet "Don't fall for it, falls can be prevented" x1 RCT  Life skills program x2 RCTs |
| **Critical appraisal of included primary studies** | PEDro Scale  Unable to extract PEDro score  EFNS  EFNS Classification II | PEDro  Range: 6-7  Adequate quality (≥5) x3 RCTs |
| **Type of analysis of falls outcomes** | Narrative synthesis | Narrative synthesis |
| **Follow-up period for relevant primary studies** | 6 months | 12 months x3 RCTs |
| **Falls outcomes** | *Total number of falls:*  The intervention did not significantly reduce falls. | *Falls rate:*  Statistically significant reduction in rate of falls over 12 months for both intervention groups receiving physiotherapy plus self-management versus the control group receiving no physiotherapy or self-management x1 RCT (2 comparisons).  Sub-analysis x1 RCT found a 69% reduction in falls in those with less advanced PD who received physiotherapy and self-management versus self-management alone.  *Number of fallers:*  No difference in number of fallers x3 RCTs.  *Number of recurrent fallers:*  No difference in number of recurrent fallers x2 RCTs. |
| **Secondary outcomes** | Secondary outcomes not extracted as falls were a secondary outcome in this review. | *QoL:*  Inconsistent findings regarding intervention effects on QoL, 3xRCTs (consisting of 4 comparisons): 2 comparisons found statistically significant improvements in QoL for intervention groups compared to control groups, 2 comparisons found no statistically significant difference between intervention and control groups.  *Physical Activity Outcomes:*  3xRCTs (consisting of 4 comparisons) found no statistically significant improvements in physical activity outcomes.  1xRCT found a significant improvement in SPPB and STS post-intervention but not in total time spent walking or number of walking periods.  *UPDRS:*  1x RCT (2 comparisons) found significant improvements in the ADL component of UPDRS but not the motor component of UPDRS for the intervention groups.  1xRCT found no change in UPDRS with intervention versus without.  *FES-I:*  1xRCT found significant improvement in FES-I for intervention compared to control. |
| **Conclusions** | Insufficient evidence to draw firm conclusions regarding the effectiveness of auditory cueing at reducing falls among people with PD. | The scarcity of published literature, plus the nature of the trials to date, make it impossible to draw any firm conclusions regarding the overall efficacy of falls self-management interventions for people with PD, or to identify the key components of these interventions.  Findings not overly representative of people with PD who falls - studies typically recruited those with lower H&Y scores and all studies excluded those with cognitive impairments. |
| **Comments** | Fall definition: N/R.  Methods of falls data collection: N/R. | Fall definition: N/R.  Faller classifications: N/R.  Methods of falls data collection: N/R. |

| **Citation** | **Rodrigues-Krause et al. (2019)** | **Winser et al. (2019)** |
| --- | --- | --- |
| **Aim of systematic review** | To review dance as a form of intervention to promote functional and metabolic health in older adults. | To identify evidence evaluating the cost-effectiveness of physiotherapy treatment techniques for people with neurological disorders.  To provide future implications for research on cost-effectiveness evaluation for physiotherapy treatment techniques in neurological rehabilitation. |
| **Search methods** | 4 databases (MEDLINE, Cochrane Wiley database, PEDro, LILACS).  1 trial registry (Clinical Trials.gov).  Databases searched from November 1980 to March 2016.  English, Spanish or Portuguese language only. | 6 databases searched (Allied and Complimentary Medicine, Ovid, Medline, Embase, Health Technology Assessment, NHS Economic Evaluation Database, CINAHL).  Database search from date of inception to July 2018.  English language only. |
| **Number of relevant primary studies** | 1 primary study:  1x Quasi-experimental nonRCT | 1x primary study:  1x RCT |
| **Date range of relevant primary studies** | 2013 | 2016 |
| **Country of origin of relevant primary studies** | N/R | New Zealand/Australia x1 RCT |
| **Participants** | n = 33  Percentage of female participants: 39%  Intervention group mean age = 68.4 +/- 7.7 years  Control group mean age = 74.4 +/- 6.5 years | n = 231  Percentage of female participants: 41.6%  Mean age = 70.7 years |
| **Setting** | N/R | Interventions delivered by physiotherapist or under the supervision of a PT. |
| **Intervention (Type, Frequency, Intensity)** | Tango dance  Session duration: 90 minutes  Intervention duration: 10-12 weeks | Group based exercise classes with home visits from physical therapist and provision of standard fall prevention booklet x1 RCT  Session frequency: classes 1 session/month and 2-4 home visits  Intervention duration: 6 months |
| **Person delivering intervention** | N/R | Directly supervised by physical therapist |
| **Comparator** | Education lessons | Usual care from medical practitioner and/or community services and standard falls prevention booklet |
| **Critical appraisal of included primary studies** | PRISMA Recommendations  Tool not applied to non-RCTs | PEDro and CHEC-list  PEDro = 6 (high)  CHEC-list = 14 (moderate) |
| **Type of analysis for falls outcomes** | Narrative synthesis | Narrative synthesis |
| **Follow-up period for relevant primary studies** | N/R | 6 months |
| **Falls outcomes** | *Total number of falls:*  Tango was 1.42 times more likely than education to provide decreased or no change in follow-up falls incidence during follow-up period compared to the year prior to the intervention.  Among participants who had improved balance, 66% of tango group and 50% of education group, experienced decreased or no change in fall incidence.  *Number of fallers:*  The number of people falling versus not falling was not significantly different between groups. | *Mean number of falls:*  Lower mean number of falls in intervention group (4.106 falls) than control group (7.053 falls). |
| **Secondary outcomes** | Secondary outcomes not extracted as falls were a secondary outcome in this review. | Secondary outcomes not extracted as falls were a secondary outcome in this review. |
| **Conclusions** | No firm conclusion regarding effectiveness of dance on falls among people with PD can be drawn from this review. | Physiotherapy treatment is potentially cost-effective per fall prevented relative to control intervention. |
| **Comments** | Outcomes informed by one non-RCT that has no RoB assessment.  Fall definition: N/R.  Methods of falls data collection: N/R. | Not reported if difference in mean number of falls between groups was significant.  Fall definition: N/R.  Methods of falls data collection: N/R. |

| **Citation** | **Mak et al. (2017)** | **Ramazinna et al. (2017)** |
| --- | --- | --- |
| **Aim of systematic review** | To investigate the long-term effects of exercise and physical therapy in people with PD. | To review the effects of resistance training, performed against a resistance different from the body weight, in PD patients.  The primary objective of this review was to assess the effectiveness of resistance training on muscle strength improvement.  The secondary objective was to determine the effects of resistance training on improving physical performance and quality of life of PD patients. |
| **Search methods** | 5 databases (CINAHL, MEDLINE, PubMed, Academic Search Premier via EbscoHost, Cochrane Library).  Databases searched from up to 15 May 2017.  No language restrictions. | 4 databases searched (PubMed, PEDro, Scopus, Web of Science).  1 trial registry (Cochrane Central Register of Controlled Trials).  Database searched up until February 2016.  English language only. |
| **Number of relevant primary studies** | 8 primary studies:  RCTs x6 (Note: 2x included studies are based on the same RCT investigating balance training, 2x included studies are based on the same RCT investigating Tai Chi) | 5 primary studies:  4xRCTs (Note: 2x included studies based on the same RCT) |
| **Date range of relevant primary studies** | 2012-2016 | 2012-2015 |
| **Country of origin of relevant primary studies** | N/R | N/R |
| **Participants** | n = 790 (range: 23 - 195)  Percentage of female participants: N/R  Age range: 40 - 89 years  H&Y stage range: 1 – 4  Medication status during assessment: On x5 RCTs, N/R x1 RCT | n = 154 (range: 29 – 51)  Percentage of female participants: N/R  Age: N/R  H&Y range: 1.5 - 3 |
| **Setting** | N/R | N/R |
| **Intervention (Type, Frequency, Intensity)** | Balance Training x4 RCTs:  Minimally supervised, progressive strengthening, balance, cueing for FOG and fall prevention advice x1 RCT  Mobility and balance training with movement strategies, fall prevention advice or progressive resistance training and fall prevention advice x1 RCT  Technology-assisted balance and mobility training x1 RCT (Note: included in 2 studies)  Balance and mobility training x1 RCT  Total training hours per week range: 2-4 hours  Total training hours range: 32-78 hours  Intervention duration range: 8-26 weeks  Complementary Exercises (Tai Chi) x2 RCTs:  Tai Chi x1 RCT  Tai Chi OR progressive strength training x1 RCT (Note: included in two studies)  Total training hours per week range: 2-3 hours  Total training hours range: 36-52 hours  Intervention duration range: 12-24 weeks | Strength training x4 RCTs:  Using pneumatic resistance equipment x1 RCT  Using dynamometers and leg-press machines, in addition to rowing exercises, repetitive step on a 6-inch curb, and walking with an ankle weight x1 RCT  Using dynamometers and leg-press machines, in addition to rowing exercises, repetitive step on a 6-inch curb, and walking with an ankle weight plus home training consisting of stepping and walking with ankle weights x1 RCT (Note: included in two studies)  Hydrotherapy with perturbation-based balance and strength training x1 RCT  Session duration range: 45 - 60 minutes  Session frequency range: 2 - 5 sessions/week  Intervention duration range: 4 - 12 weeks |
| **Person delivering intervention** | N/R | N/R |
| **Comparator** | Balance Training:  Usual care x1 RCT  Life-skills information x1 RCT  Strengthening exercises x1 RCT (Note: included in 2 studies)  No training x1 RCT  Complementary Exercises:  Usual care x1 RCT  Stretching x1 RCT (Note: included in two studies) | N/R x1 RCT  Repetitive step training and visual cues x1 RCT  Balance training (technology-assisted) x1 RCT (Note: included in 2 studies)  Physiotherapy x1 RCT |
| **Critical appraisal of included primary studies** | Cochrane Risk of Bias Tool  High-quality (three or more bias domains with low risk) x8 studies | PEDro  Only high-quality studies (≥6) were included in this SR  Range 7-8  2x studies scored 7  2x studies scored 8  Note: all five relevant primary studies scored 'no' for 'blind subjects' and 'blind therapists' |
| **Type of analysis for falls outcomes** | Narrative synthesis | Narrative synthesis |
| **Follow-up period for relevant primary studies** | Balance Training:  Range: Immediately post-intervention - 52 weeks  Complementary Exercises:  Range: 13 - 26 weeks | Range: Immediately post-intervention - 12 months |
| **Falls outcomes** | Balance Training:  *Fall Rate:*  Significant reduction in falls rate (-37%) reported immediately after training completion x1 RCT.  Significant reduction in falls rate (-81%) reported at 3-month follow-up x1 RCT.  Significant reduction in falls rate (-61.5%) reported at one year follow-up x1 RCT.  Fall rate was reduced (by 69%) only in participants with mild PD x1 RCT (suggesting that a minimally supervised falls prevention intervention could be beneficial in this subgroup).  85% reduction in falls rate in progressive resistance training group x1 RCT (suggested due to functional nature of their training and the inclusion of fall education).  Complementary Exercises (Tai Chi):  *Fall Rate:*  Significant reduction in falls rate (-53%) reported at 13-week follow-up x1 RCT.  Significant reduction in falls rate for Tai Chi group (-69%) and Progressive Resistance Training group (-60%) reported at 26-week follow-up x1 RCT. | *Total number of falls:*  1x RCT reported a significant reduction in number of falls for both the hydrotherapy (strength training) and physiotherapy groups, however, there was a greater reduction in falls in the hydrotherapy group with a significant between-group difference identified.  3x RCTs reported no significant reduction in number of falls with strength training.  1x RCT reported a significant reduction in falls with balance training (control). |
| **Secondary outcomes** | Balance Training:  *Balance:*  Significant improvement in miniBEST score x1 RCT.  Significant improvement in latency of postural response x1 RCT.  Significant improvement in single-leg-stance performance x1 RCT.  Significant improvement in balance confidence x2 RCTs.  *Disease Severity:*  Significant improvement in UPDRS-II and UPDRS-III scores during 12-months of follow-up x1 RCT.  *Gait Performance/Mobility:*  Significant improvement in stride length x1 RCT.  Significant improvement in 5XSTS x1 RCT.  Significant improvement in SPPB x1 RCT.  *Attrition rate:*  Dropouts ranged from 0-31% in the balance training interventions.  Complementary Exercises (Tai Chi):  *Balance:*  Significant improvement in BBS x1 RCT.  Significant improvement in max excursion x1 RCT.  Significant improvement in functional reach distance x1 RCT.  *Disease Severity:*  Significant improvement in UPDRS-II x1 RCT.  Significant improvement in UPDRS-III x1 RCT.  *Muscle Strength:*  Significant improvement in knee muscle strength x1 RCT.  *Mobility:*  Significant improvement in gait speed x1 RCT.  Significant improvement in stride length x1 RCT.  *Attrition rate:*  Dropouts ranged from 5-10% in the Tai Chi interventions. | Secondary outcomes not extracted as falls were a secondary outcome in this review. |
| **Conclusions** | Balance training for people with PD appears effective at reducing falls, in addition to improving balance and mobility, for up to one year after intervention completion.  Tai Chi for people with PD appears effective at reducing falls, in addition to improving balance, for up to six months after intervention completion.  Balance training for people with PD had the longest carry-over effects. | Heterogeneity between interventions and outcomes preclude firm conclusions.  Further research is needed to investigate the effects of strength training for people with PD. |
| **Comments** | Significant limitation of this SR is the inability to extract falls outcomes from RCTs that had a non-significant effect.  RCTs that had no significant effect classified under balance performance and falls - unclear for each RCT if it was a balance outcome or falls outcome or both that had non-significant outcome.  13 x RCTs reported has having non-significant effect on balance performance and falls (1 x Multimodal Physical Therapy; 3 x Progressive Resistance Therapy; 2 x Aerobic Training; 2 x Cued Exercise; 3 x Balance Training; 2 x Tai Chi).  Additionally, 9 x RCTs were reported as having a significant effect on balance performance, unclear if there was a non-significant effect on falls outcomes or if falls outcomes were just not reported (1 x Gait Training; 2 x Balance Training; 1 x Tai Chi; 5 x Dance).  Fall definition: N/R.  Methods of falls data collection: N/R.  Most commonly recruited participants for included studies had mild to moderate PD (H&Y stage 2-3). | Fall definition: N/R.  Methods of falls data collection: N/R. |

| **Citation** | **Song et al. (2017)** | **Shen et al. (2016)** |
| --- | --- | --- |
| **Aim of systematic review** | To investigate the effects of Tai Chi/Qigong on motor and non-motor function, and quality of life in people with PD. | To examine the effects of exercise on improving balance and gait ability and reducing falls among people with PD over the short-term and long-term. |
| **Search methods** | 7 databases (PubMed, CINAHL, Web of Science, ProQuest Central, Science Direct, Scopus, Cochrane Library).  Databases searched up until 30^th^ August 2016.  English language only. | 5 databases (CINAHL, MEDLINE, PubMed, Academic Search Premier via EBSCO, Cochrane Library).  Databases searched up until 8^th^ May 2015.  No language restrictions. |
| **Number of relevant primary studies** | 3 primary studies:  2x RCTs  1x One-group pretest-posttest | 8 primary studies:  8x RCTs (9 interventions) |
| **Date range of relevant primary studies** | 2014-2015 | 2007-2015 |
| **Country of origin of relevant primary studies** | USA x2 studies (1x RCT, 1x NRSI)  China x1 RCT | N/R |
| **Participants** | n = 305 (range: 34-195)  Percentage of female participants: 38%  Mean age range: 66 - 69.5 years  Medication status during assessment: On x3 studies (2x RCTs, 1x NRSI) | n = 925 (range: 64-231)  Percentage of female participants: 38%  Age range: 61.6 (+/-8) - 72.2 (+/-9.2) years  H&Y range: 1-4  Fall risk: higher x4 RCTs, lower x4 RCTs (marked as a higher risk of falling if inclusion criteria included history of falling in the past 12 months, experiencing FOG in the past month, gait disturbance, postural instability with balance and mobility problems, reduced lower limb strength or fall-related balance confidence)  Medication status during assessment: On x8 RCTs |
| **Setting** | N/R | Facility, community and home-based interventions |
| **Intervention (Type, Frequency, Intensity)** | Tai Chi x2 RCTs  Duration of session: 1 hour (x2 RCTs)  Session frequency range: 2-3 sessions/week  Intervention duration range: 12 – 24 weeks  Qigong x1 NRSI  Duration of session: 1 hour  Session frequency: 1 session/week  Intervention duration: 12 weeks | Balance, gait, strength, other exercises x2 RCTs (other exercises are exercises not specifically targeted at enhancing balance and gait performance, such as active joint mobility and muscle stretching exercises)  Gait x1 RCT  Balance x2 RCTs  Balance, strength x1 RCT  Strength x1 RCT  Balance, gait x2 RCTs  Training location: home x 3RCTs, facility x4 RCTs, facility and home x1 RCTs  Training supervision: ≤ half x2 RCTs, > half x6 RCTs  Training hours per week range: 1- 4 hours  Total training hours range: 4.5 - 48 hours  Intervention duration range: 3 - 36 weeks |
| **Person delivering intervention** | N/R | N/R |
| **Comparator** | Resistance training or stretching x1 RCT  No treatment x1 RCT | No training x7 RCTs  Other exercises x1 RCT (other exercises are exercises not specifically targeted at enhancing balance and gait performance, such as active joint mobility and muscle stretching exercises) |
| **Critical appraisal of included primary studies** | 10-Item Cochrane Collaboration Tool (RCTs only)  Scoring: methodologically strong (0-1 risk); methodologically moderate (2-3 risk); methodologically weak (4 risk)  Score range: 1-2 (2 RCTs) | Cochrane Risk of Bias Tool  Primary studies were of moderate to high quality (low risk of bias in 3 or more domains) |
| **Type of analysis of falls outcomes** | Fixed-effects meta-analysis | Random- and fixed-effects meta-analyses |
| **Follow-up period for relevant primary studies** | Range: 6 – 9 months | Range: Immediately post-intervention - 52 weeks |
| **Falls outcomes** | *Total number of falls:*  ES = -0.403  95% CI = [-0.677, -0.129]  p = 0.004  I^2^ = 0%  2 RCTs informing outcome, n = 271  Tai Chi significantly reduced fall episodes compared to control group in PD.  1x NRSI reported that the PD patients experienced fewer falls at the end versus the beginning of a 6-month Qigong programme. | **Short-term fall rate:*  RaR = 0.485  95% CI = [0.329, 0.715]  p < 0.001  I^2^ = 62%  4 RCTs informing outcome (5 comparisons), n = 605  The fall rate showed a significant overall reduction over the short-term.  ***Long-term fall rate:*  RaR = 0.413  95% CI = [0.270, 0.630]  p < 0.001  I^2^ = 61%  5 RCTs informing outcome (6 comparisons), n = 451  The fall rate showed a significant overall reduction over the long-term.  **Short-term number of fallers:*  Risk ratio = 0.939  95% CI = [0.822, 1.072]  p = 0.349  I^2^ = 0%  4 RCTs informing outcome (5 comparisons), n = 707  The number of fallers did not decrease significantly over the short-term.  ***Long-term number of fallers:*  Risk ratio = 0.787  95% CI = [0.605, 1.024]  p = 0.075  I^2^ = 48%  3 RCTs informing outcome, n = 345  The number of fallers did not decrease significantly over the long-term.  *Short-term = the immediate effects after treatment completion  **Long-term = the carryover effects at follow-up intervals |
| **Secondary outcomes** | *UPDRS III:*  1xRCT found significant effect for Tai Chi intervention compared to control for UPDRS III (ES -0.539, 95% CI -0.992 to -0.086, p = 0.020).  *Balance:*  1xRCT found no significant effect for Tai Chi intervention compared to control for balance (ES 0.425, 95% CI -0.025 to 0.876, p = 0.064).  *TUG:*  1xRCT found significant effect for Tai Chi intervention compared to control for TUG (ES -0.506, 95% CI -0.958 to -0.053, p = 0.029).  *Cost-effectiveness:*  1xRCT found that Tai Chi is a cost effective approach for fall prevention in PD. | *Short-term effects on postural stability:*  Statistical significance in favour of intervention for BBS x2 RCTs.  No significant difference in BBS between groups x3 RCTs (4 comparisons).  No significant difference in comfortable walking velocity between groups x1 RCT.  No significant difference in TUG between groups x2 RCTs.  *Long-term effects on postural stability:*  Statistical significance in favour of intervention for BBS x2 RCTs.  No significant difference in BBS between groups x1 RCT.  No significant difference in comfortable walking velocity between groups x1 RCT.  Statistical significance in favour of intervention for TUG x1 RCT (2 comparisons).  No significant difference in TUG between groups x1 RCT. |
| **Conclusions** | Limited evidence suggests Tai Chi and Qigong are effective at reducing falls among people with PD. | Exercise training can decrease fall rates among people with PD over both the short- and long-terms. |
| **Comments** | Fall definition: N/R  Methods of falls data collection: N/R. | Faller classification used in review: person who fell during the study period.  Faller classifications used in primary studies: N/R.  Fall definition: N/R.  Methods of falls data collection: N/R. |

| **Citation** | **Tomlinson et al. (2014)** | **Tomlinson et al. (2012a)** |
| --- | --- | --- |
| **Aim of systematic review** | To assess the effectiveness of one physiotherapy intervention compared with a second approach in people with PD. | To assess the effectiveness of physiotherapy intervention compared with no intervention or placebo in patients with PD. |
| **Search methods** | 13 databases (Cochrane Movement Disorders Specialised Register, The Cochrane Library, Medline, CINAHL, ISI-SCI, AMED, REHABDATA, REHADAT, PEDro, GEROLIT, LILACS, MedCarib, IMEMR).  7 trial registries (CENTRAL, CentreWatch Clinical Trials listing service, metaRegister of Controlled Trials, ClinicalTrials.gov, RePORT, NIDRR, NRR).  6 grey literature databases (Conference Proceedings Citation Index, DISSABS, Conference Papers Index, Index to Theses, EThOS, ProQuest).  Abstract books and conference proceedings (The XIII International Congress on Parkinson's disease, The International Congress of Parkinson's Disease and Movement Disorders, World Congress on Parkinson's Disease and Related Disorders, The American Academy of Neurology 51st annual meeting).  Databases searched up until 2012.  No language restrictions. | 13 databases (Cochrane Movement Disorders Specialised Register, The Cochrane Library, Medline, CINAHL, ISI-SCI, AMED, REHABDATA, REHADAT, PEDro, GEROLIT, LILACS, MedCarib, IMEMR).  7 trial registries (CENTRAL, CentreWatch Clinical Trials listing service, metaRegister of Controlled Trials, ClinicalTrials.gov, RePORT, NIDRR, NRR).  6 grey literature databases (Conference Proceedings Citation Index, DISSABS, Conference Papers Index, Index to Theses, EThOS, ProQuest).  Abstract books and conference proceedings (The XIII International Congress on Parkinson's disease, The International Congress of Parkinson's Disease and Movement Disorders, World Congress on Parkinson's Disease and Related Disorders, The American Academy of Neurology 51st annual meeting).  Databases searched up until 2010.  No language restrictions. |
| **Number of relevant primary studies** | 3 primary studies:  3x RCTs (parallel group design) | 7 primary studies:  7x RCTs (2x had cross-over design and 5x had parallel design) |
| **Date range of relevant primary studies** | 2010-2012 | 2002-2010 |
| **Country of origin of relevant primary studies** | USA x1 RCT  Australia x1 RCT  Italy x1 RCT | N/R |
| **Participants** | n = 469 (range: 64 - 210)  Percentage of female participants: 36%  Mean age range: 67.3 - 69 years  Mean disease duration: 6.7 - 10.4 years  Inclusion criteria: H&Y 1-4 x2 RCTs, H&Y 3-4 x1 RCT  Exclusion criteria: MMSE < 24 x2 RCTs, MMSE < 23 x1 RCT | n = 532 (range: 18 - 153)  Percentage of female participants: 38% female across 6x RCTs (N/R x1 RCT, male participants only x1 RCT)  Mean age range: 63.4 - 73.7 years (N/R x1 RCT)  Mean H&Y range: 2 - 3.14 (N/R x2 RCTs)  Mean disease duration range: 4.7 - 9.1 years (N/R x2 RCTs)  Medication status during assessment: On x3 RCTs, Midway between drug doses x1 RCT, No constraints on time of assessment x1 RCT, N/R x2 RCTs |
| **Setting** | Physiotherapy interventions aiming to maximise functional ability and minimise secondary complications.  Inclusive in the definition of physiotherapy intervention (including those not directly delivered by a physiotherapist) with trials of general physiotherapy, exercise, treadmill training, cueing, dance and martial arts being included.  Outpatients intervention x2 RCTs, setting not reported x1 RCT | Physiotherapy interventions aiming to maximise functional ability and minimise secondary complications.  Inclusive in the definition of physiotherapy intervention (including those not directly delivered by a physiotherapist) with trials of general physiotherapy, exercise, treadmill training, cueing, dance and martial arts being included.  Outpatients x6 RCTs, Home-based x1 RCT |
| **Intervention (Type, Frequency, Intensity)** | Tai Chi (eight form routine designed to tax balance and gait through movements such as weight-shifting, controlled displacement of the centre of mass over the base of support, ankle sways, and anterior-posterior and lateral stepping) or resistance training (focused on strengthening muscles that are important for posture, balance and gait, through progressive resistance with weighted vests or ankle weights, on exercises including forward and side steps, squats, forward and side lunges, and heel and toe raises) x1 RCT  Movement strategy training and individualised home practice session and weekly structured falls risk education and a single home visit to check compliance (task specific practice of functional actions such as rolling over, standing up, walking, crossing obstacles and turning, with strategies such as visual and auditory cues, mental rehearsal and movement planning, conscious attention during the task, and breaking the task into a sequence of smaller components) or progressive strength training and individualised HEP and once weekly structured falls risk education and a single home visit to check compliance (strengthening exercises for quadriceps, hip and trunk extensor muscles, hip abductors, calf, and ankle dorsiflexors, completed performing functional tasks where possible and weight added to progress) x1 RCT  Balance training (exercises aimed at improving feedforward and feedback postural reactions, three groups of exercises including: self-destabilisation of the centre of body mass; externally induced destabilisation of the centre of body mass; and emphasis of coordination between leg and arm movements during walking as well as locomotor dexterity over an obstacle course and other potentially destabilising activities) x1 RCT  Session duration range: 50 minutes - 2 hours  Session frequency range: 2 - 3 sessions/week  Intervention duration range: 7 - 24 weeks | Exercise (personalised home exercises comprised of muscle strengthening, range of motion, balance training, and walking, and strategy programme consisting of falls prevention strategies and compensation - physiotherapists performed house visits with exercises performed while they were there) x1 RCT  Exercise (supervised group strength and balance training plus unsupervised home exercises) x1 RCT  Martial arts (Tai Chi classes) x2 RCTs  Exercise (collaborated with fitness instructors to design an individualised, progressive exercise programme) x1 RCT  Cueing (cueing device provided three rhythmical cueing modalities: auditory; visual; and somatosensory, participants tried all three and trained with their preferred modality on tasks aimed to improve step length and walking speed, prevent freezing episodes and improve balance) x1 RCT  Treadmill (using a harness for safety, participants walked at fastest speed forward, backward and sideways, and then performed step training which consisted of turning the treadmill on suddenly to perturb the participant's standing balance; backward, forward, and left and right perturbations were performed) x1 RCT  Session duration range: 30 - 60 minutes (not reported x2 RCTs)  Session frequency range: 1 - 7 sessions/week (not reported x 1 RCT)  Intervention duration range: 3 - 12 weeks  Total intervention hours: 4.5 - 42 hours (not reported x2 RCTs) |
| **Person delivering intervention** | N/R x2 RCTs  Trained therapist or nurse x1 RCT | PT x1 RCT  Fitness instructor x1 RCT  N/R x5 RCTs |
| **Comparator** | Stretching (variety of seated and standing stretches of the upper body and lower extremities) x1 RCT  Life-skills control group and a once weekly home session of reflection activities and relaxation practice (led by OTs, PTs, Speech pathologists or social workers and will include content such as relaxation, games, communication activities and guided discussion on topics such as the impact of PD) x1 RCT  General exercises (exercises not specifically designed to improve postural reactions: active joint mobilisation, muscle stretching and motor coordination exercises) x1 RCT | Usual care, contact with PD nurse x1 RCT  Usual care x2  Baseline exercise programme that was not progressed x1 RCT  No training/intervention/treatment x3 RCTs |
| **Critical appraisal of included primary studies** | Risk of bias assessed on the following domains: eligibility criteria, randomisation method, concealment of allocation, similarity at baseline, intention-to-treat analysis, withdrawals described, co-interventions constant, comparable arms, blinded assessors)  1x RCT had high risk of bias on concealment of allocation and unclear risk of bias on three other domains  1x RCT had unclear risk of bias on four domains and low risk of bias on the others  1x RCT had high risk of bias on concealment of allocation and withdrawals described and unclear risk of bias on four other domains | Risk of bias assessed on the following domains: eligibility criteria, method of randomisation and blinding, concealment of allocation, similarity at baseline, intention-to-treat analysis, description of withdrawals, co-interventions constant, credible placebo, blinded assessors  Quality of included reviews was varied.  1x RCT scored unclear risk of bias for two domains and low risk of bias for the others  1x RCT scored high risk of bias for blinded assessors and unclear risk of bias for three other domains  1x RCT scored unclear risk of bias for five domains and low risk of bias for the others  1x RCT scored unclear risk of bias for one domain and low risk of bias for all others  1x RCT scored high risk of bias for allocation concealment and low risk of bias for all other domains  1x RCT scored unclear risk of bias for four domains and low risk of bias for all other domains  1x RCT scored unclear risk of bias for six domains and low risk of bias for the other two |
| **Type of analysis of falls outcomes** | Narrative synthesis | Narrative synthesis |
| **Follow-up period for relevant primary studies** | Range: 1 - 12 months | Range: Immediately post-intervention to 30 weeks. |
| **Falls outcomes** | *Total number of falls:*  No difference between Tai Chi group and resistance training group on number of falls x1 RCT.  Number of falls during the intervention phase (8 weeks) was significantly lower in the progressive strength training arm compared to the movement strategy training arm (n = 10 versus n = 24, p = 0.006), with the frequency of falls varying markedly.  No significant difference in number of falls between the balance training arm and the general exercises arm x1 RCT  *Time to first fall:*  No significant difference in time to first fall between the progressive strength training arm and the movement strategy training arm. | Exercise-based interventions:  *Number of falls:*  1x RCT reported a significant reduction in number of falls for the Tai Chi intervention group compared with no intervention.  5x RCTs reported no significant effect of intervention on number of falls, however there was a general trend towards reduction in number of falls with physiotherapy.  Cueing interventions:  *Number of falls:*  There was no significant effect of intervention on number of falls. |
| **Secondary outcomes** | *Mobility:*  No difference between Tai Chi group and resistance training group on gait speed x1 RCT.  *UPDRS Motor Subscale:*  No significant effect identified between groups x2 RCTs.  *Adverse events:*  No event significant enough to cause concern over safety of the intervention x2 RCTs.  *Attrition rate:*  Dropouts ranged from 7-14% across interventions. | Exercise-based interventions:  *Attrition rate:*  Dropouts ranged from 0-10% in the exercise interventions.  Cueing interventions:  *Attrition rate:*  Dropouts were less than 1% in the cueing intervention. |
| **Conclusions** | Given the heterogeneity between studies and the methodological flaws of included primary studies, there is insufficient evidence to support the use of one physiotherapy intervention over another. | There was a general trend towards a reduction in falls with intervention, however, this was not significant in the majority of included studies.  Data on falls was poorly reported and there was substantial variation between studies on how falls were measured and so could not be meta-analysed.  Further research on the effectiveness of physiotherapy at reducing falls among people with PD is required before firm conclusions can be drawn. |
| **Comments** | Fall definition: N/R.  Methods of falls data collection N/R x1 RCT.  Participants kept written record of falls x2 RCTs.  Number of fallers, number of multiple fallers, number of injurious falls and falls rate were listed as outcomes in x1 RCT but this data was N/R in the review. | Fall definition: N/R.  Methods of falls data collection: falls diary x3 RCTs, fall frequency form x1 RCT, report of falls x1 RCT, N/R x2 RCTs. |

| **Citation** | **Monti et al. (2011)** | **Winser et al. (2018)** |
| --- | --- | --- |
| **Aim of systematic review** | To research the effectiveness of physiotherapy intervention on the prevention of falls among people with PD. | To determine whether Tai Chi training improves balance and reduces falls incidence when compared to control conditions of either active treatment or no treatment in people with neurological diseases. |
| **Search methods** | 3 databases (PubMed, CINAHL, The Cochrane Collaboration).  Databases searched from 2000 to 2010.  No language restrictions. | 6 databases (AMED, Embase, Web of Science, SCOPUS, EBSCO, Medline).  Databases searched from date of inception to 28 February 2018.  No language restrictions. |
| **Number of relevant primary studies** | 5 primary studies:  5x RCTs | 4 primary studies:  4x RCTs |
| **Date range of relevant primary studies** | 2005-2009 | 2011-2014 |
| **Country of origin of relevant primary studies** | Australia x1 RCT  Turkey x1 RCT  Ireland x1 RCT  UK x1 RCT  USA x1 RCT | Hong Kong and China x1 RCT  USA x3 RCTs |
| **Participants** | n = 456 (range: 18-230)  Percentage of female participants: N/R  Mean age range: 71.8 +/- 6.4 years to 72.5 years (mean age N/R x3 RCTs)  Age range: 44-91 years (age range N/R x4 RCTs)  3x RCTs required participants to be autonomous in walking.  3x RCTs required participants to have a history of falls. | PD:  n = 288 (range 17 - 195)  Percentage of female participants: 36%  Age: 72 +/- 8.5 years (N/R x2 RCTs)  Stroke:  n = 145  Percentage of female participants: 47%  Age: 69.9 +/- 10 years |
| **Setting** | Physiotherapy treatments only included.  Setting: N/R | N/R |
| **Intervention (Type, Frequency, Intensity)** | Exercise-based interventions x4 RCTs:  Exercises for postural control to strengthen the muscles of the legs, exercises for balance, cueing (auditory, cognitive, somatic sensorial) with the integration in the ADL plus received a booklet with advice for the prevention of falls (participants receiving intervention divided into two groups, the first was support group based where there were group exercises with the physiotherapist and exercises at home autonomously, the second was home based mode where exercises were performed at home autonomously with periodic control by the physiotherapist) x1 RCT  Walking on a treadmill, exercise to increase ROM and stretching exercises x1 RCT  Exercises to strengthen the muscles of the legs, to increase the ROM for the superior and inferior areas, for the equilibrium, for walking outdoor x1 RCT  Walking on the treadmill in the four directions and at a faster speed than the one used on usual ground x1 RCT  Session duration range: 40-60 minutes (2x RCTs N/R)  Session frequency range: 2-7 sessions/week (1x RCT N/R)  Intervention duration range: 6 weeks - 12 months (1x RCT N/R)  Cueing interventions x1 RCT:  Cueing intervention x1 RCT (Note: unable to extract specific details regarding intervention due to error in Table 2 in systematic review)  Session duration: as much as possible during the day  Session frequency: as much as possible during the day  Intervention duration range: 3 weeks | PD:  24-form of Yang style Tai Chi x1 RCT  Yang style short form of Tai Chi x1 RCT  Tai Chi, six movements with eight-form routine x1 RCT  Session duration: 60 minutes x3 RCTs  Session frequency range: 2-3 sessions/week  Intervention duration range: 12-24 weeks  Stroke:  24-form of Yang style Tai Chi x1 RCT  Session duration: 60 minutes  Session frequency: 3 sessions/week  Intervention duration: 24 weeks |
| **Person delivering intervention** | PT x5 RCTs | PD:  Trained Tai Chi instructor x3 RCTs  Stroke:  Trained Tai Chi instructor x1 RCT |
| **Comparator** | Exercise-based interventions:  Standard medical care plus received a booklet with advice for the prevention of falls x1 RCT  Standard pharmacological therapy x2 RCTs  Standard treatment and treatments with a nurse specialised in PD x1 RCTs  Cueing interventions:  Unable to extract details regarding control due to error in Table 2 in systematic review x1 RCT | PD:  No treatment x2 RCTs  Control 1 - resistance training, Control 2 - stretching x1 RCT  Session duration: 60 minutes  Session frequency: 2 sessions/week  Control duration: 24 weeks  Stroke:  Control 1 - group based aerobic exercises, Control 2 - usual care x1 RCT  Session duration: 60 minutes  Session frequency: 3 sessions/week  Control duration: 12 weeks |
| **Critical appraisal of included primary studies** | PEDro  Range of included studies: 5-9 | PEDro  High x4 RCTs (6 or more) |
| **Type of analysis of falls outcomes** | Narrative synthesis | Fixed-effects meta-analysis |
| **Follow-up period for relevant primary studies** | Exercise-based interventions:  Range: Immediately post-intervention to 6 months  Cueing interventions:  3 months | PD:  Range: 12-36 weeks  Stroke:  12 weeks |
| **Falls outcomes** | Exercise-based interventions:  *Number of falls:*  4x RCTs report a reduction in the number of fall episodes for the intervention groups.  The intervention group had 30% less episodes of falls compared to the control group (Incident RR = 0.70) x1 RCT.  The intervention group had a significant decrease in number of falls x1 RCT.  The intervention group had a significant decrease in number of falls after 8 weeks and after 6 months x1 RCT.  The intervention group had a 38% decrease in the number of falls x1 RCT.  Cueing interventions:  *Number of falls:*  The intervention group had a decrease in the number of falls in the ADL x1 RCT. | PD:  *Total number of falls - Tai Chi versus active therapies*:  OR = 0.47  95% CI = [0.29, 0.77]  p = 0.003  I^2^ = 39%  2 RCTs informing outcome (with 3 comparisons), n = 212  GRADE: High  There was a statistically significant effect of Tai Chi compared with active therapies on total number of falls.  *Total number of falls - Tai Chi versus no treatment:*  OR = 0.29  95% CI = [0.11, 0.79]  p = 0.02  1 RCT informing outcome, n = 76  GRADE: High  There was a statistically significant effect of Tai Chi compared with no treatment on total number of falls.  Stroke:  *Total number of falls - Tai Chi versus active therapies:*  OR = 0.21  95% CI = [0.09, 0.48]  p = 0.0003  I^2^ = 0%  1 RCT informing outcome (2 comparisons), n = 145  GRADE: High  There was a statistically significant effect of Tai Chi compared with active therapies on total number of falls. |
| **Secondary outcomes** | Exercise-based interventions:  *Falls risk:*  The intervention group had a 26% decrease of risk of falls x1 RCT.  *Near falls:*  The intervention group had a significant decrease in near falls x1 RCT.  *Balance Function:*  The intervention group had a 23% greater improvement in balance than the control group x1 RCT.  The intervention group had a significant improvement in performance of the FRT x1 RCT.  *Fear of Falling:*  The intervention group had a significant decrease in fear of falling x1 RCT.  *Psychological Measures:*  The intervention group had a significant increase in the rate of FES-I x1 RCT.  *Mobility:*  The intervention group had a significant increase in gait speed on the treadmill and in the covered distance x1 RCT.  The intervention group had an increase in gait speed and in length of step, and both the intervention and control groups had an increase in the pace of the step and increase in speed in the 5-step tests x1 RCT.  *QoL:*  The intervention group had a sinificant improvement in QoL x1 RCT.  *Strength:*  The intervention group had a 11% greater increase in muscular power of the extensors of the knee compared to the control group x1 RCT.  Cueing interventions:  *Mobility:*  The intervention group had improvements in gait speed, in the length and the pace of the steps x1 RCT. | PD:  *Attrition rate:*  Less than 15% dropouts reported x3 RCTs.  Stroke:  *Attrition rate:*  Less than 15% dropouts reported x1 RCT. |
| **Conclusions** | The findings of this review support the effectiveness of physiotherapy interventions at reducing falls among people with PD.  The lack of methodologically strict large experimental trials precludes drawing firm conclusions.  No evidence was found to support the use of one intervention over the other. | This systematic review found high methodological quality and high GRADE evidence for the effectiveness of Tai Chi to reduce falls incidence among people with PD, however the evidence is limited.  Data from one individual study found Tai Chi was beneficial for reducing falls incidence among people with stroke. |
| **Comments** | Note: number of falls reported as an outcome measure in two other studies included in this review (1x RCT, 1x pilot study) but the findings of these falls outcomes are not reported (unclear if it is because the findings were insignificant.  Fall definition: N/R  Methods of falls data collection: falls diary x3 RCTs, N/R x2 RCTs | Fall definition: N/R.  Methods of falls data collections: N/R. |

**Abbreviations:**

MS = Multiple Sclerosis

RCT = Randomised Controlled Trial

N/R = Not Reported

EDSS = Expanded Disability Status Scale

NRSI = Non-Randomised Study of Intervention

SPMS = Secondary Progressive Multiple Sclerosis

FES = Functional Electrical Stimulation

JBI = Joanna Briggs Institute

BBS = Berg Balance Scale

SOT = Sensory Organisation Test

FSST = Four Square Step Test

ABC = Activities-Specific Balance Confidence

DHI = Dizziness Handicap Inventory

TUG = Timed Up and Go

DGI = Dynamic Gait Index

PPA = Physiological Profile Assessment

FSS = Fatigue Severity Scale

HEP = Home Exercise Programme

WEBB = Weight-bearing Exercise for Better Balance

STS = Sit-To-Stand

ROM = Range Of Motion

PT = Pysiotherapist

OT = Occupational Therapist

tDCS = Transcranial Direct Current Stimulation

GP = General Practitioner

HIFE = High-Intensity Functional Exercise

SD = Standard Deviation

RaR = Rate Ratio

CI = Confidence Interval

GRADE = Grading of Recommendations, Assessment, Development and Evaluations

RR = Risk Ratio

QoL = Quality of Life

AAP = Adelaide Activities Profile

SF-12 = 12-Item Short Form Survey

SF-36 = 36-Item Short Form Survey

FAI = Frenchay Activities Index

SIPSO = Subjective Index of Physical and Social Outcome

HADS = Hospital Anxiety and Depression Scale

GHQ-28 = General Health Questionnaire-28

PASIPD = Physical Activity Scale for Individuals with Physical Disabilities

NHP = Nottingham Health Profile

CES-D = Center for Epidemiological Studies Depression Scale

PSQI = Pittsburgh Sleep Quality Index

MSSE = Multiple Sclerosis Self-Efficacy

MSIS-29 = Multiple Sclerosis Impact Scale

SMD = Standardised Mean Difference

MD = Mean Difference

FES-I = Falls Efficacy Scale-International

TUG-C = Timed Up and Go-Cognitive

SDMT = Symbol Digit Modalities Test

TMT – Trail Making Test

MSWS-12 = 12-item Multiple Sclerosis Walking Scale

6MWT = 6 Minute Walk Test

2MWT = 2 Minute Walk Test

T25FW = Timed 25-Foot Walk

10MWT = 10 Minute Walk Test

ADL = Activities of Daily Living

MFIS = Modified Fatigue Impact Scale

FSMC = Fatigue Scale for Motor and Cognition

SADQ-10 = Stroke Aphasic Depression Questionnaire

MSQOL-54 = Multiple Sclerosis Quality of Life-54

MDT = Multi-Disciplinary Team

SLT = Speech and Language Therapist

SW = Social Worker

EFNS = European Federation of Neurological Societies

SPPB = Short Physical Performance Battery

UPDRS = Unified Parkinson's Disease Rating Scale

PRISMA = Preferred Reporting Items for Systematic Reviews and Meta-Analysis

CHEC = Consensus on Health Economic Crtieria

miniBEST = Mini Balance Evaluation Systems Test

5XSTS = 5 Times Sit-To-Stand

ES = Effect Size

MMSE = Mini–Mental State Examination

FRT = Functional Reach Test
